# Supplementary material for: Computational design of ultra-robust strain sensors for soft robot perception and autonomy
Source: Nat Commun. 2024 Feb 22;15:1636. doi: 10.1038/s41467-024-45786-y (PMC10883982; doi:10.1038/s41467-024-45786-y)
Supplement: Supplementary file 1 — Supplementary Information [file 41467_2024_45786_MOESM1_ESM.pdf]

Supporting Information

Computational design of ultra-robust strain sensors for soft robot perception and autonomy

Haitao Yang,‡[a,b] Shuo Ding,‡[c,d] Jiahao Wang,‡[b] Shuo Sun,[e] Ruphan Swaminathan,[f] Serene Wen Ling Ng,[b] Xinglong Pan,[b] Ghim Wei Ho[b]*

[a] Institute of Flexible Electronics (IFE) & Frontiers Science Center for Flexible Electronics, Northwestern Polytechnical University, Xi'an, Shaanxi, 710072 China.

[b] Department of Electrical and Computer Engineering, National University of Singapore, Singapore, 4 Engineering Drive 3, Singapore 117583, Singapore.

[c] College of Mechanical and Electrical Engineering, Nanjing University of Aeronautics and Astronautics, Nanjing, 210016, China.

[d] Department of Biomedical Engineering, National University of Singapore, Singapore, 4 Engineering Drive 3, Singapore 117583, Singapore.

[e] Department of Mechanical Engineering, National University of Singapore, Singapore, 9 Engineering Drive 1, Singapore 117575, Singapore.

[f] Department of Computer Science, Columbia University, New York 10027, USA.

‡ These authors contribute equally to this work.

*Email: elehgw@nus.edu.sg (G.W.H.)

This PDF file includes:

Supplementary Figs. 1 to **66**

Supplementary Tables 1 to **9**

Supplementary Notes 1 to **14**

**Supplementary Fig. 1**	Characterization of SWNT.	7
**Supplementary Fig. 2**	Characterization of the SWNT layer at a 0.5 mg mass loading.	8
**Supplementary Fig. 3**	Fabrication process of a PCAM sensor.	9
**Supplementary Fig. 4**	Three interdigital crack patterns consist of interlocking comb-shaped crack arrays.	10
**Supplementary Fig. 5**	A programmed interdigital crack pattern on the SWNT-coated PS device.	11
**Supplementary Fig. 6**	Changes of the shrinkage ratio of the PS shrink film under different heating time.	12
**Supplementary Fig. 7**	Structure evolutions of a PCAM sensor under uniaxial strain loading.	13
**Supplementary Fig. 8**	Performance comparison between crack pattern and trace pattern-based sensors.	14
**Supplementary Fig. 9**	Micro-crumple structure of PCAM sensor with different φ values	15
**Supplementary Fig. 10**	Sensing profiles of a PCAM sensor (ρ=1,200 µm mm^{-2}; φ=40%) under repeated uniaxial strain loading from 20% to 70%.	16
**Supplementary Fig. 11**	Sensing stability of a PCAM sensor (ρ=1,200 µm mm^{-2}; φ=40%) under bending and twisting.	17
**Supplementary Fig. 12**	Hysteresis profiles of a PCAM sensor (ρ=1,200 µm mm^{-2}; φ=40%) under uniaxial strain loading from 20% to 70%.	18
**Supplementary Fig. 13**	Sensing profile and SEM images of a PCAM sensor (ρ=1,200 µm mm^{-2}; φ=40%) under a stretching, twisting, stretching, bending, stretching mechanical loading.	19
**Supplementary Fig. 14**	Sensing profile and SEM images of a planar sensor under a stretching, twisting, stretching, bending, stretching mechanical loading.	20
**Supplementary Fig. 15**	Sensing profiles of a PCAM sensor under intermittent cyclic loading (1-10 cyclic rounds).	21
**Supplementary Fig. 16**	Sensing profiles of a PCAM sensor under intermittent cyclic loading (11-20 cyclic rounds).	22
**Supplementary Fig. 17**	Sensing profiles of a planar sensor under intermittent cyclic loading.	23
**Supplementary Fig. 18**	Sensing profiles of a crumpled sensor under intermittent cyclic loading.	24
**Supplementary Fig. 19**	Sensing profile of a PCAM sensor (ρ=1,200 µm mm^{-2}; φ=40%) under a static stretching state (30% strains) over 30 minutes.	25

**Supplementary Fig. 20**	Setups to test sensor stability under dynamic stretching frequencies.	26
**Supplementary Fig. 21**	Sensor stability under 23 Hz stretching frequency.	27
**Supplementary Fig. 22**	Digital photo and sensing profile of sensor-integrated origami robot when it was meeting an obstacle.	28
**Supplementary Fig. 23**	Digital photo and sensing profile of sensor-integrated origami robot when it was crawling from desktop to a rough paper.	29
**Supplementary Fig. 24**	Digital photo and sensing profile of sensor-integrated origami robot when it was crawling from desktop to a watery surface.	30
**Supplementary Fig. 25**	Fabrication and sensor integration of a pneumatic robot.	31
**Supplementary Fig. 26**	Digital photos and sensing profiles of a PCAM sensor-integrated pneumatic soft robot when it was moving forward, turning left, and turning right.	32
**Supplementary Fig. 27**	Digital photos and sensing profiles of a PCAM sensor-integrated pneumatic soft robot when it was meeting an obstacle.	33
**Supplementary Fig. 28**	Digital photos and sensing profiles of a PCAM sensor-integrated pneumatic soft robot when it was crawling on different friction surfaces, including desktop, sand road, and mud road.	34
**Supplementary Fig. 29**	Digital photo of a pneumatic soft robot in an artificial terrain.	35
**Supplementary Fig. 30**	Actuation system design of the pneumatic soft robot.	36
**Supplementary Fig. 31**	Position tracking of the pneumatic soft under trajectory 1, which was repeated 5 times.	37
**Supplementary Fig. 32**	Position tracking of the pneumatic soft under trajectory 2, which was repeated 5 times.	38
**Supplementary Fig. 33**	Sensing profiles of a PCAM sensor-integrated pneumatic soft robot under trajectory 1, which was repeated 5 times.	39
**Supplementary Fig. 34**	Sensing profiles of a PCAM sensor-integrated pneumatic soft robot under trajectory 2, which was repeated 5 times.	40
**Supplementary Fig. 35**	Sensing performance of a PCAM sensor-integrated pneumatic soft robot under trajectory 1, which was repeated 5 times.	41
**Supplementary Fig. 36**	Sensing performance of a PCAM sensor-integrated pneumatic soft robot under trajectory 2, which was repeated 5 times.	42
**Supplementary Fig. 37**	Sensing profiles of a planar and crumpled sensor-integrated pneumatic soft robot under trajectory 1, which was repeated 5 times.	43

**Supplementary Fig. 38**	Sensing profiles of a planar and crumpled sensor-integrated pneumatic soft robot under trajectory 2, which was repeated 5 times.	44
**Supplementary Fig. 39**	Fabrication of the tetrapod microrobot.	45
**Supplementary Fig. 40**	Sensing profile of a tetrapod microrobot under repeated magnet actuation.	46
**Supplementary Fig. 41**	Origami robot with four on-body sensors.	47
**Supplementary Fig. 42**	Actuation system design of the origami robot.	48
**Supplementary Fig. 43**	Position tracking of the origami robot under repeated 5 times navigation.	49
**Supplementary Fig. 44**	Robot location/trajectory tracking by a camera system.	50
**Supplementary Fig. 45**	Performance of the trajectory prediction model.	51
**Supplementary Fig. 46**	Prediction performance of an extra ANN model by using only robot actuation information as the training data.	52
**Supplementary Fig. 47**	One PCAM sensor was attached at the central of robot head to realize the sensing capability of terrain altitude changes.	53
**Supplementary Fig. 48**	Cartesian coordinates and boundary conditions of FEA simulation.	54
**Supplementary Fig. 49**	Characterization of arithmetical mean height (R_a).	55
**Supplementary Fig. 50**	Crease pattern and digital photo of origami units.	56
**Supplementary Fig. 51**	Mechanical stability of the Ecoflex-coated origami unit.	57
**Supplementary Fig. 52**	Working window of “Tracker” software.	58
**Supplementary Fig. 53**	Kangaroo setup to simulate the origami structure's motions and deformations.	59
**Supplementary Fig. 54**	Working mechanism of ANN model.	60
**Supplementary Fig. 55**	Digital photo of SWNT layer with 0.4 mg mass loading.	61
**Supplementary Fig. 56**	Mass loading optimization of a PCAM sensor.	62
**Supplementary Fig. 57**	Relative signal variation of PCAM sensors based on three sensor replicas.	63

**Supplementary Fig. 58**	The etching depth of SWNT layer under the laser beam power of 0.06 mW.	64
**Supplementary Fig. 59**	Noise strain tolerance of PCAM sensor.	65
**Supplementary Fig. 60**	Sensing profiles of sensor-integrated origami robot when it was turning around.	66
**Supplementary Fig. 61**	Simulation of origami deformation during turning.	67
**Supplementary Fig. 62**	Digital photo and sensing profile of the origami robot from our previous work under a 35-cm trajectory.	68
**Supplementary Fig. 63**	Simulated stress distribution maps of PCAM sensors.	69
**Supplementary Fig. 64**	MSE of trajectory prediction model based on the training data from PCAM sensor, crumpled sensor, and planar sensor.	70
**Supplementary Fig. 65**	Prediction results of trajectory prediction model trained by data based on PCAM sensor, crumpled sensor, and planar sensor, respectively.	71
**Supplementary Fig. 66**	Sensor position optimization on origami robot.	72
**Supplementary Table 1**	Data summary of Fig. 6D.	72
**Supplementary Table 2**	The state-of-the-art soft crawling robot perception by mechanical sensors.	73
**Supplementary Table 3**	Details of stickiness factors during FEA modelling tasks.	74
**Supplementary Table 4**	Details of sensor structure parameters during FEA modelling tasks of different PCAM sensors.	74
**Supplementary Table 5**	Comparison among the features of origami robot, pneumatic robot, and microrobot in this work.	74
**Supplementary Table 6**	Comparison among the sensing characteristics of PCAM sensor and our previous work.	74-75
**Supplementary Table 7**	Comparison among the GF of PCAM sensor and some recent self-healing strain sensors.	75
**Supplementary Table 8**	Comparison between this work and other recent works that coupled strain sensors and ML algorithm to identify different hand gestures or body pose.	75
**Supplementary Table 9**	Performance of robotic trajectory prediction model with different k_fold values.	76
**Supplementary Note 1**	Optimization of SWNT mass loading.	77
**Supplementary Note 2**	Sensor reproducibility with high crack density.	77

**Supplementary Note 3**	PCAM sensor with GF > 200.	78
**Supplementary Note 4**	Discussion to future reduce the sensor hysteresis.	78
**Supplementary Note 5**	Noise strain tolerance of PCAM sensor.	78
**Supplementary Note 6**	Actuation and control of the origami robot.	78-79
**Supplementary Note 7**	Sensor signal analyses of origami robot.	79-80
**Supplementary Note 8**	Actuation and control of the pneumatic robot.	80
**Supplementary Note 9**	Reasons for selecting origami robot as robot autonomy demonstration.	80-81
**Supplementary Note 10**	Test of system error of the origami robot actuation.	81
**Supplementary Note 11**	Comparisons of the pros and cons between PCAM sensors and our previous work.	81-82
**Supplementary Note 12**	Comparisons between PCAM sensors and recent self-healing sensors.	82-83
**Supplementary Note 13**	Comparisons of the ML methodologies between this work and existing related reports.	83-84
**Supplementary Note 14**	The optimization of k_fold value in this work.	84
**Supporting references**		85-86

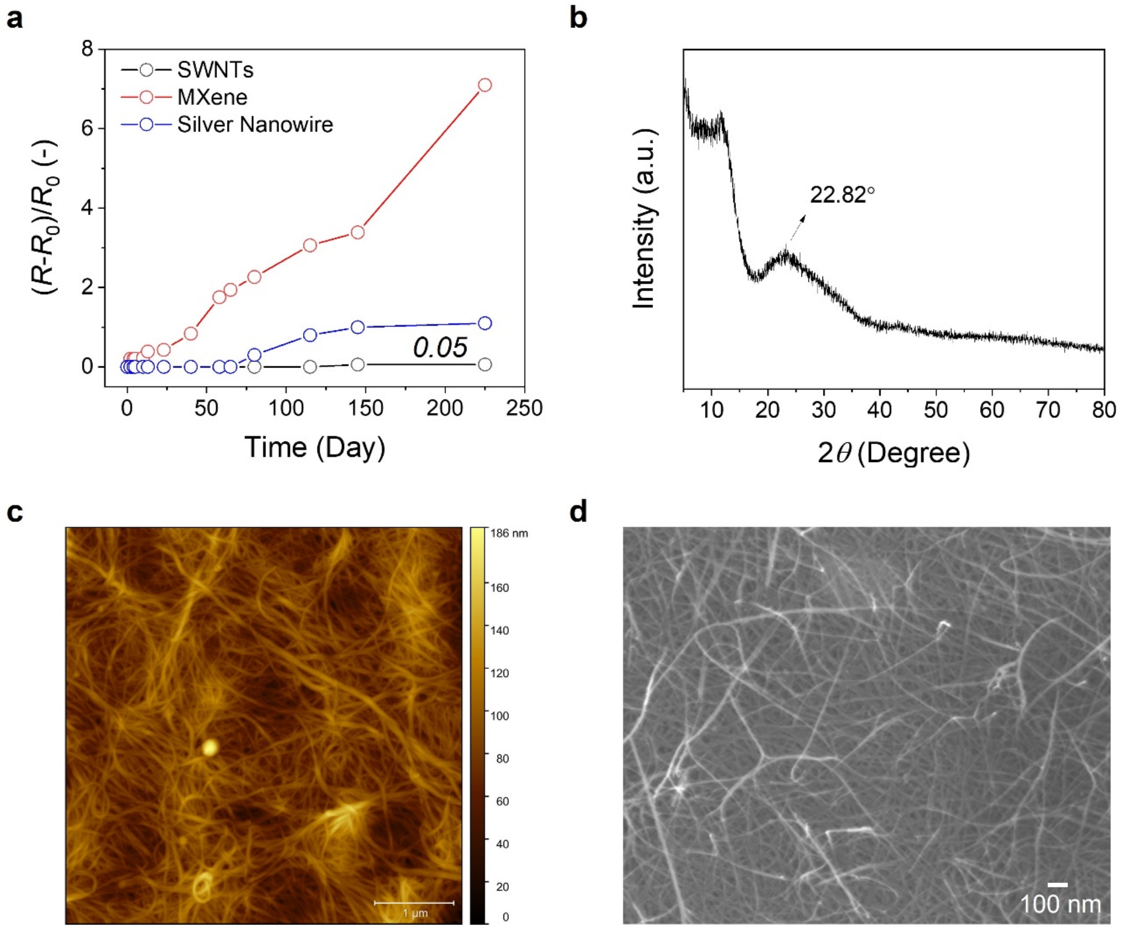

Supplementary Fig. 1. Characterization of SWNT. (**a**) Relative resistance changes of SWNT, MXene, and sliver nanowire films under room temperature. Among those nanomaterials, SWNT exhibit superior conductivity stability, making them the ideal choice for piezoresistive sensor fabrication in this study. (**b**) XRD pattern of SWNT with a representative peak of 22.82°. (**c**) AFM image of a SWNT film. (**d**) SEM image of a SWNT film.

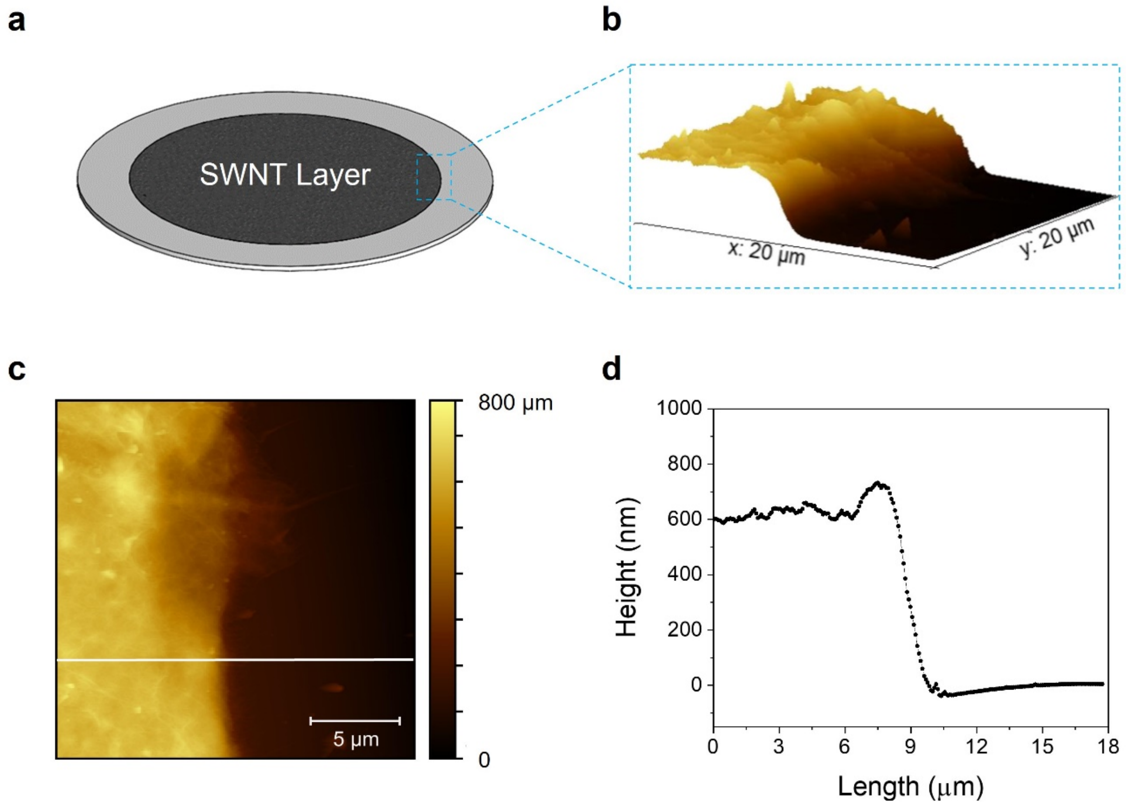

Supplementary Fig. 2. Characterization of the SWNT layer at a 0.5 mg mass loading. (**a**) Schematic illustration of SWNT layer on a PVDF membrane. (**b**) (**c**) AFM images of the SWNT layer within the square position in (**a**). (**d**) Depth profile of the SWNT layer along the white line in (**c**).

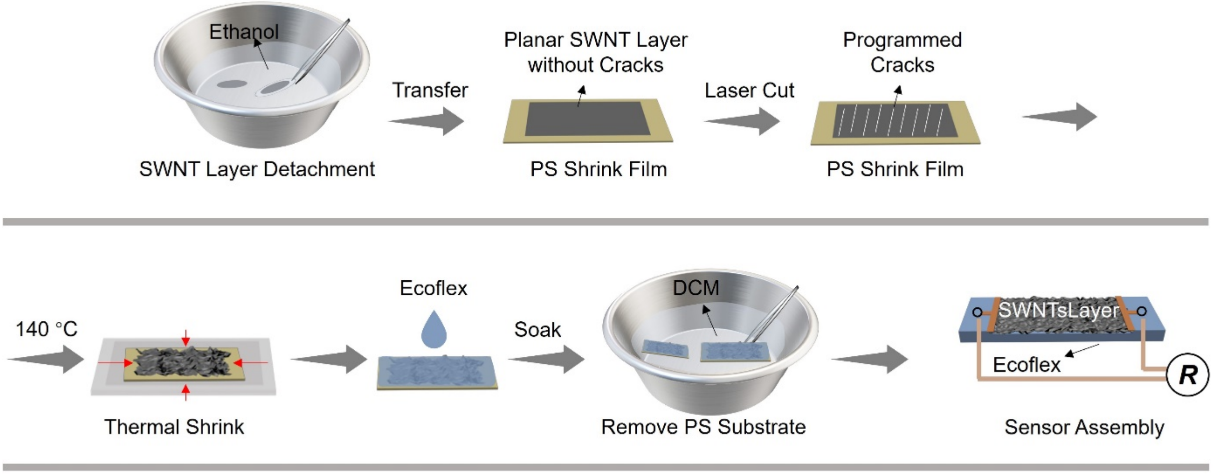

Supplementary Fig. 3. Fabrication process of a PCAM sensor. The as-prepared SWNT dispersion was deposited onto PVDF membranes through vacuum-assisted filtration systems. To remove SDS residues, the filtered SWNT thin films were rinsed with excessive DI water. Afterward, freestanding SWNT layers were detached from the PVDF membranes by immersing them in ethanol. The freestanding SWNT layers were then carefully transferred from ethanol onto the plasma-treated PS shrink films followed by overnight drying. The SWNT-coated PS device was then put into the design space of a laser machine. After the setup of laser beam power (i.e., 0.08 mW), user-defined interdigital patterns that consist of the interlocking comb-shaped crack array were sintered on SWNT-coated PS device, while the PS substrate was kept integral. With the programmed interdigital crack pattern, the SWNT-coated PS device was heated in an oven at 140 °C to induce thermal contraction. By harnessing surface instability during thermal contraction, the SWNT layers were deformed with localized micro-crumples. The shrunk samples were then coated by a 2 mm thick Ecoflex (00-35). Afterward, it was immersed in an ethyl acetate bath for 24 hours to dissolve the PS substrate. Next, the Ecoflex-coated SWNT device was taken out followed by air drying and wiring electrical leads to obtain the corresponding PCAM sensor. Silver paste was applied between SWNT layers and copper wires to ensure good electrical contact.

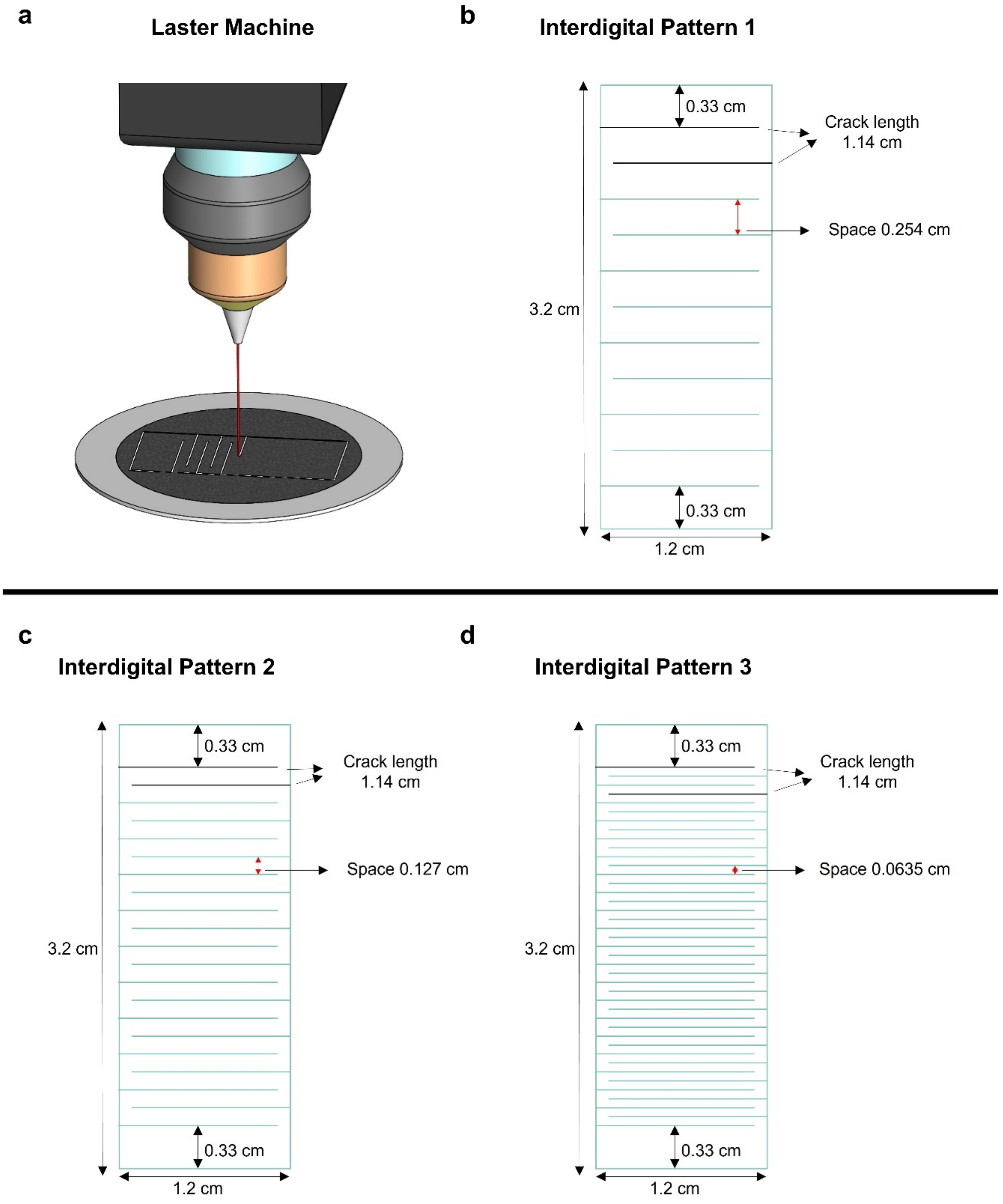

Supplementary Fig. 4. Three interdigital crack patterns consist of interlocking comb-shaped crack arrays. (**a**) Schematic illustration of laser processing. (**b**) Parameters of interdigital pattern 1. (**c**) Parameters of interdigital pattern 2. (**d**) Parameters of interdigital pattern 3.

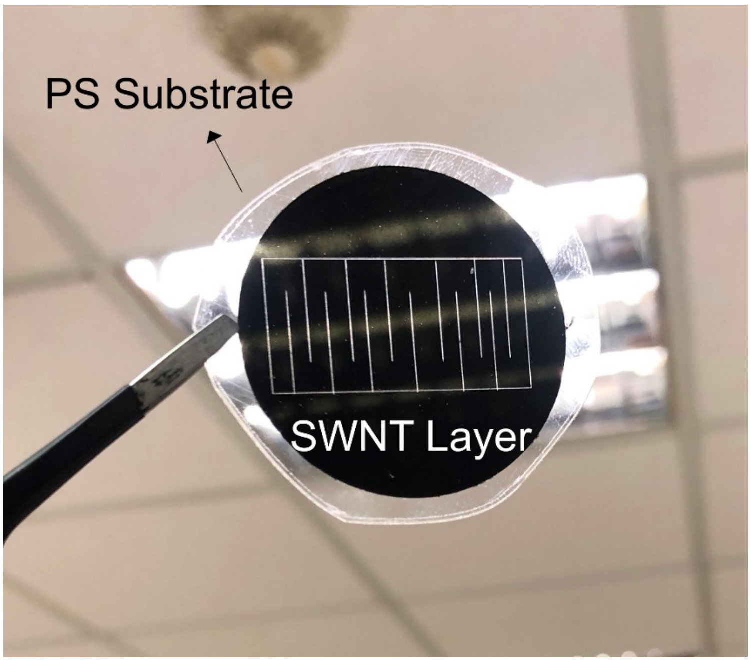

Supplementary Fig. 5. A programmed interdigital crack pattern on the SWNT-coated PS device. After the setup of laser beam power (i.e., 0.08 mW), user-defined interdigital patterns that consist of the interlocking comb-shaped crack array were sintered on SWNT-coated PS device, while the PS substrate was kept integral.

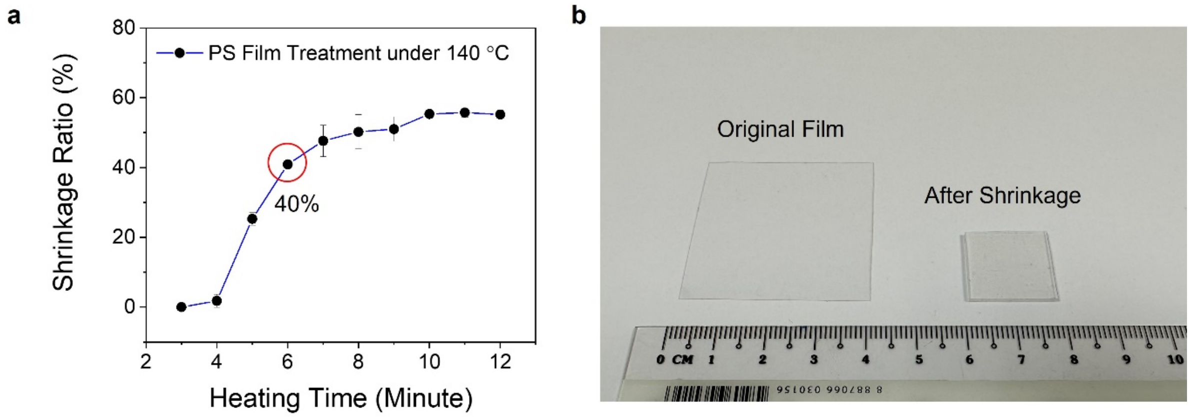

Supplementary Fig. 6. Changes of the shrinkage ratio of the PS shrink film under different heating time. (**a**) Shrinkage ratio profile of a PS film under 140 °C heating. (**b**) Digital photos of a PS film before and after the thermal shrinkage.

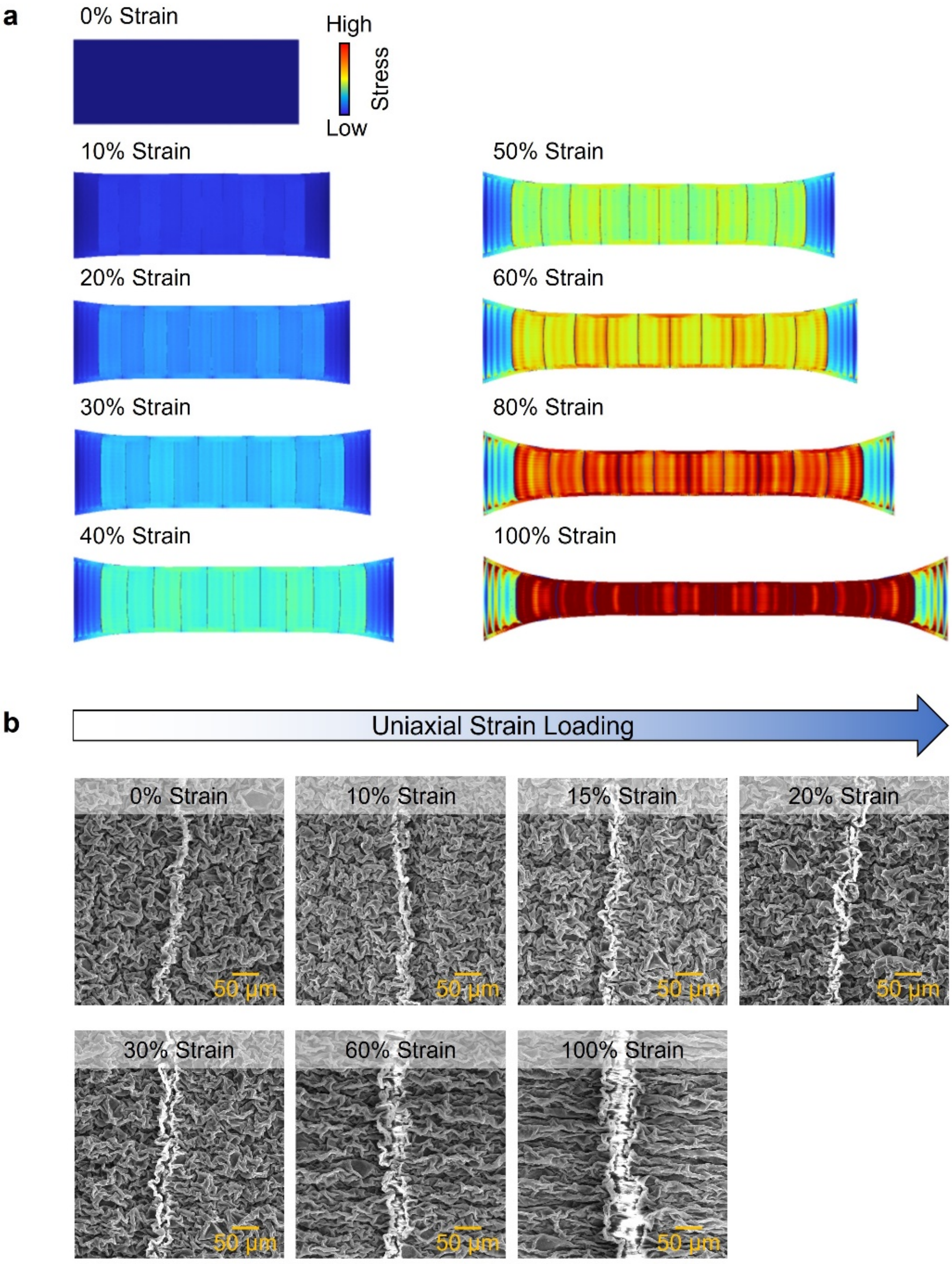

Supplementary Fig. 7. FEA simulation (a) and structure evolution (b) of a PCAM sensor under uniaxial strain loading. (ρ=300 μm mm^{-2}; φ=40%).

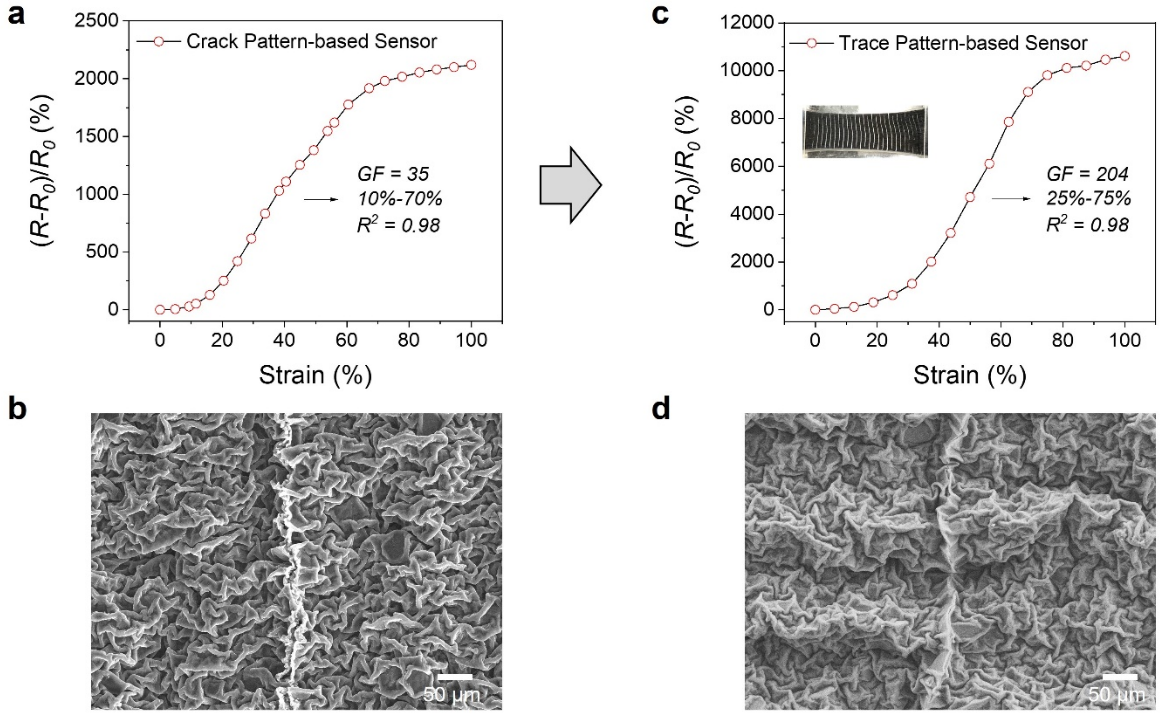

Supplementary Fig. 8. Performance comparison between crack pattern and trace pattern-based sensors. (**a**) Sensing profile of a PCAM sensor with a crack pattern (crack density: 1,200 µm mm^{-2}; φ=40%). (**b**) SEM images of a PCAM sensor with a crack pattern (crack density: 1,200 µm mm^{-2}; φ=40%). (**c**) Sensing profile of a PCAM sensor with a trace pattern (trace density: 1,200 µm mm^{-2}; φ=40%). (**d**) SEM images of a PCAM sensor with a trace pattern (trace density: 1,200 µm mm^{-2}; φ=40%).

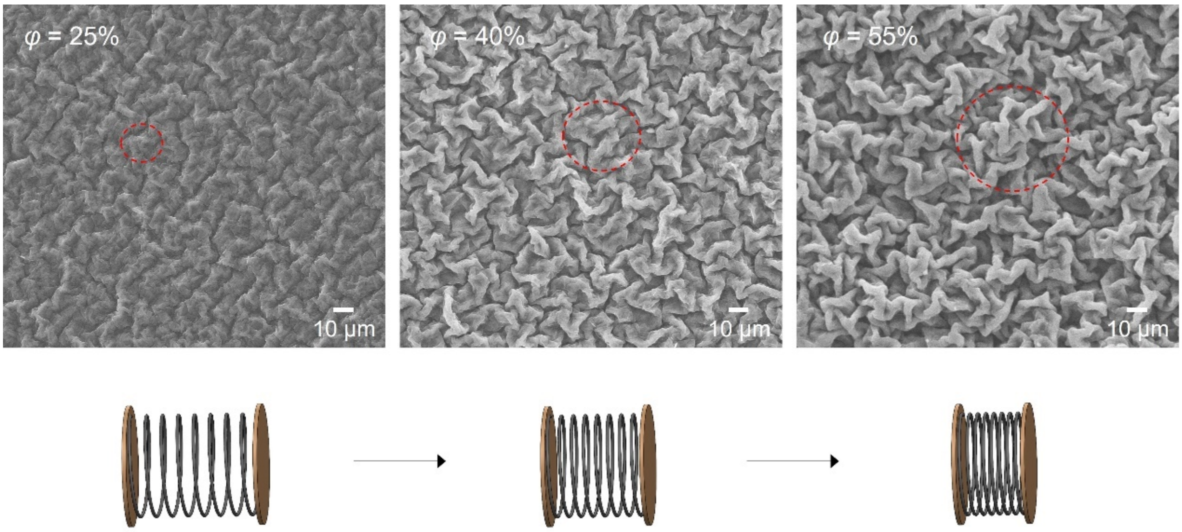

Supplementary Fig. 9. Micro-crumple structure of PCAM sensor with different φ values. With higher φ values, the size of micro-crumples was enlarged, which was corresponding to a higher stored elastic force like a more compressed spring.

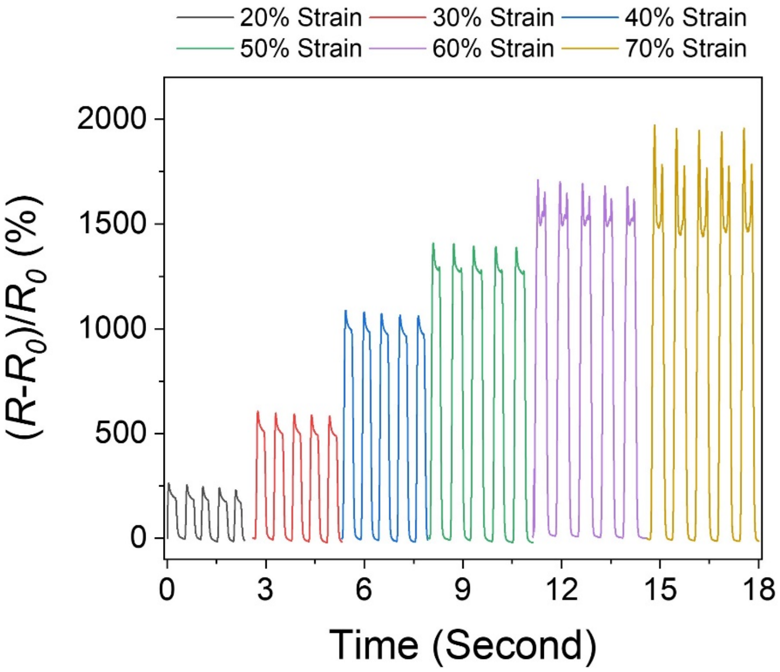

Supplementary Fig. 10. Sensing profiles of a PCAM sensor (*ρ*=1,200 µm mm^{-2}; *φ*=40%) under repeated uniaxial strain loading from 20% to 70%.

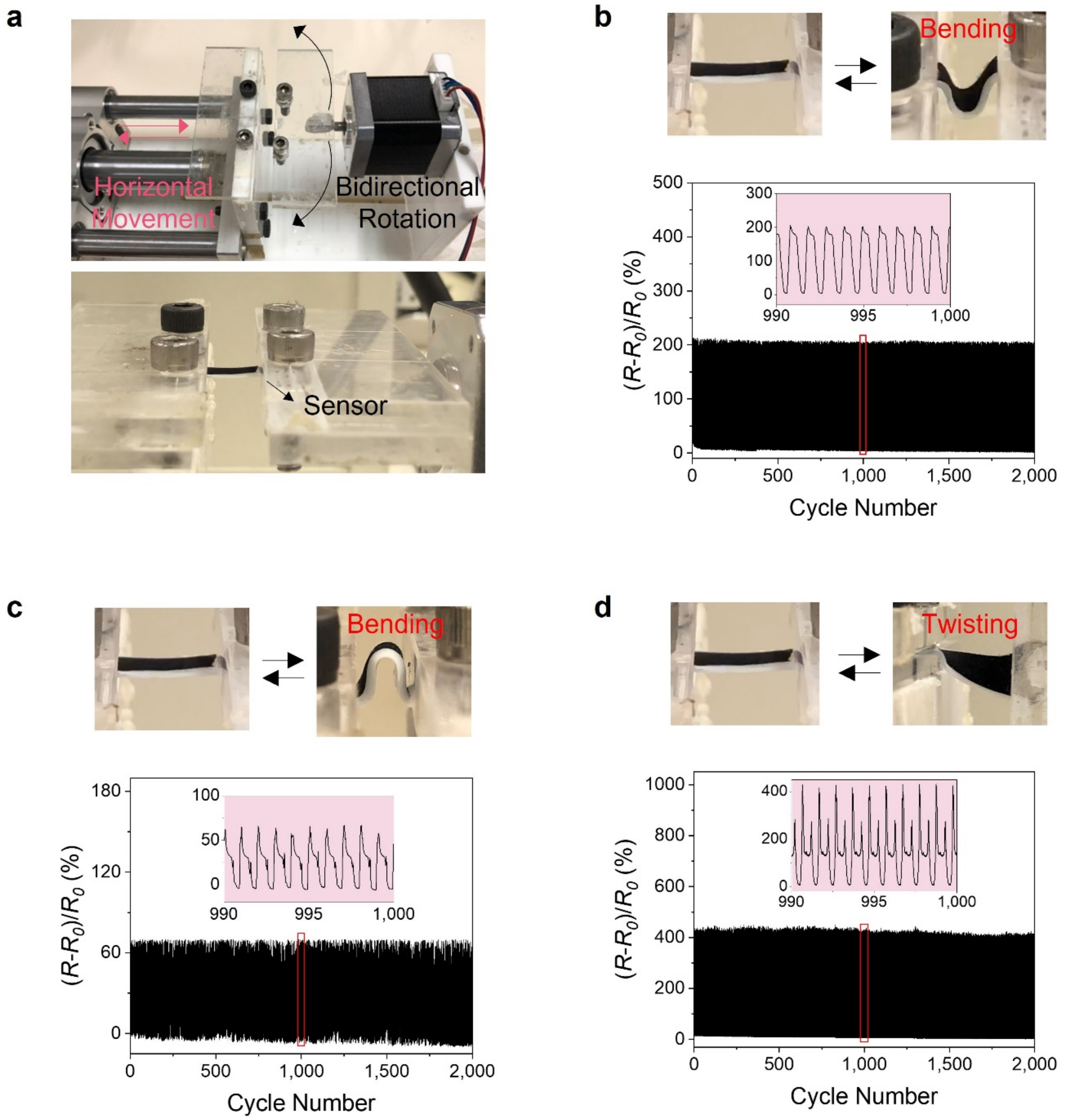

Supplementary Fig. 11. Sensing stability of a PCAM sensor (ρ=1,200 µm mm^{-2}; φ=40%) under bending and twisting. (**a**) Setup of the test machine. (**b**) Sensing profile of a PCAM sensor under the bending mode (bend down, -50% strain). (**c**) Sensing profile of a PCAM sensor under the bending mode (bend up, -65% strain). (**d**) Sensing profile of a PCAM sensor under the twist mode (twisting from 90° to -90°).

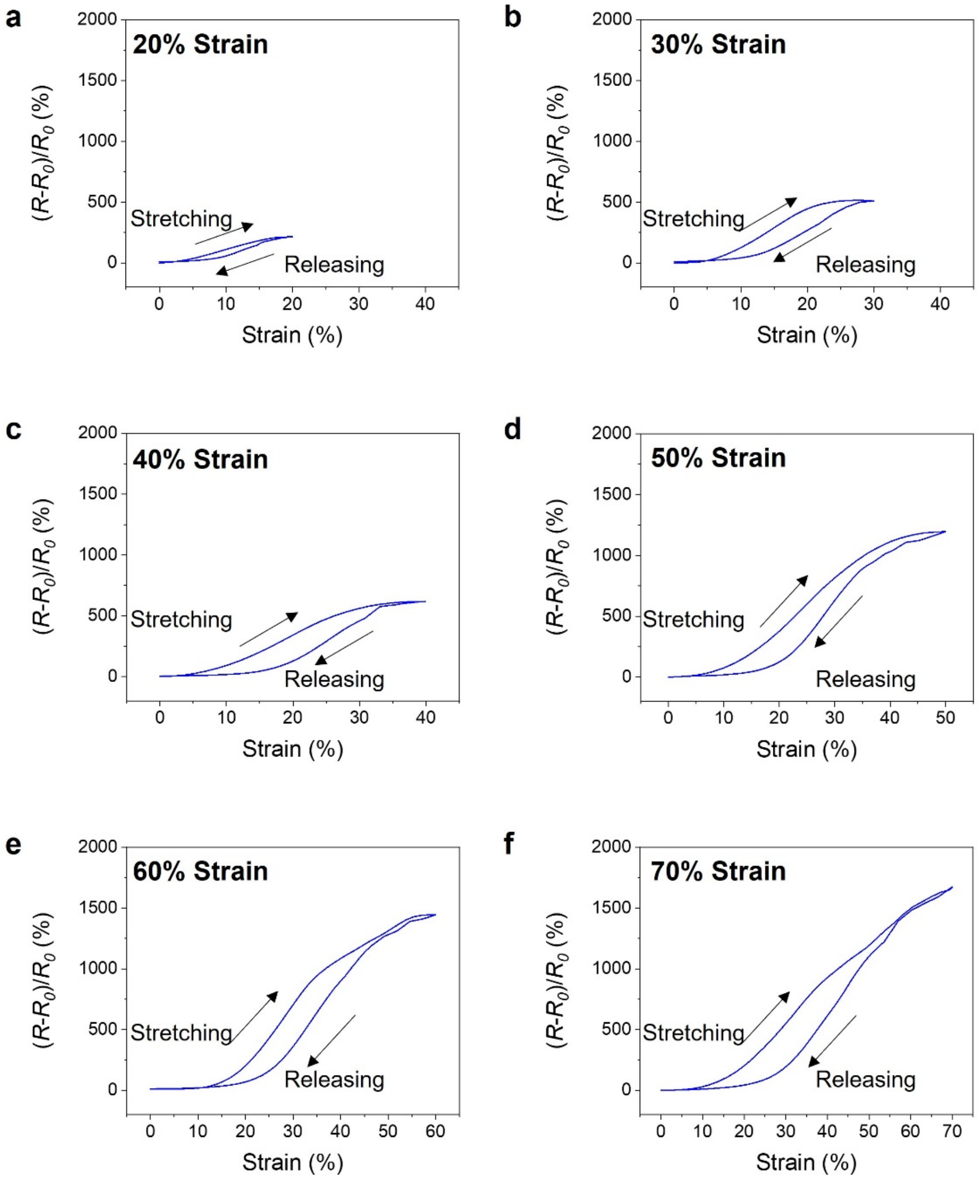

Supplementary Fig. 12. Hysteresis profiles of a PCAM sensor (ρ=1,200 µm mm^{-2}; φ=40%) under uniaxial strain loading from 20% to 70%. (**a**) Hysteresis profile under 20% strain. (**b**) Hysteresis profile under 30% strain. (**c**) Hysteresis profile under 40% strain. (**d**) Hysteresis profile under 50% strain. (**e**) Hysteresis profile under 60% strain. (**f**) Hysteresis profile under 70% strain.

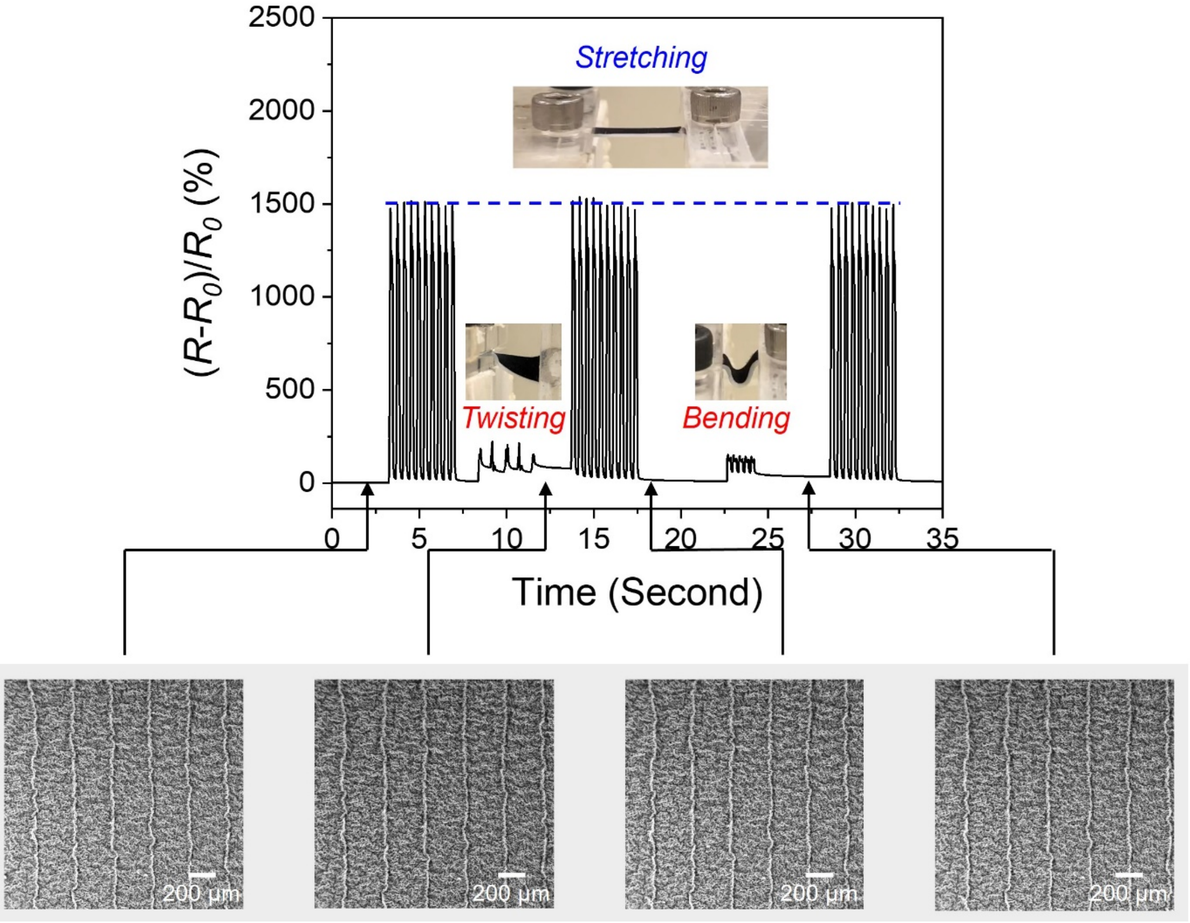

Supplementary Fig. 13. Sensing profile and SEM images of a PCAM sensor (ρ=1,200 µm mm^{-2}; φ=40%) under a stretching, twisting, stretching, bending, stretching mechanical loading. The stretching strain was 50%.

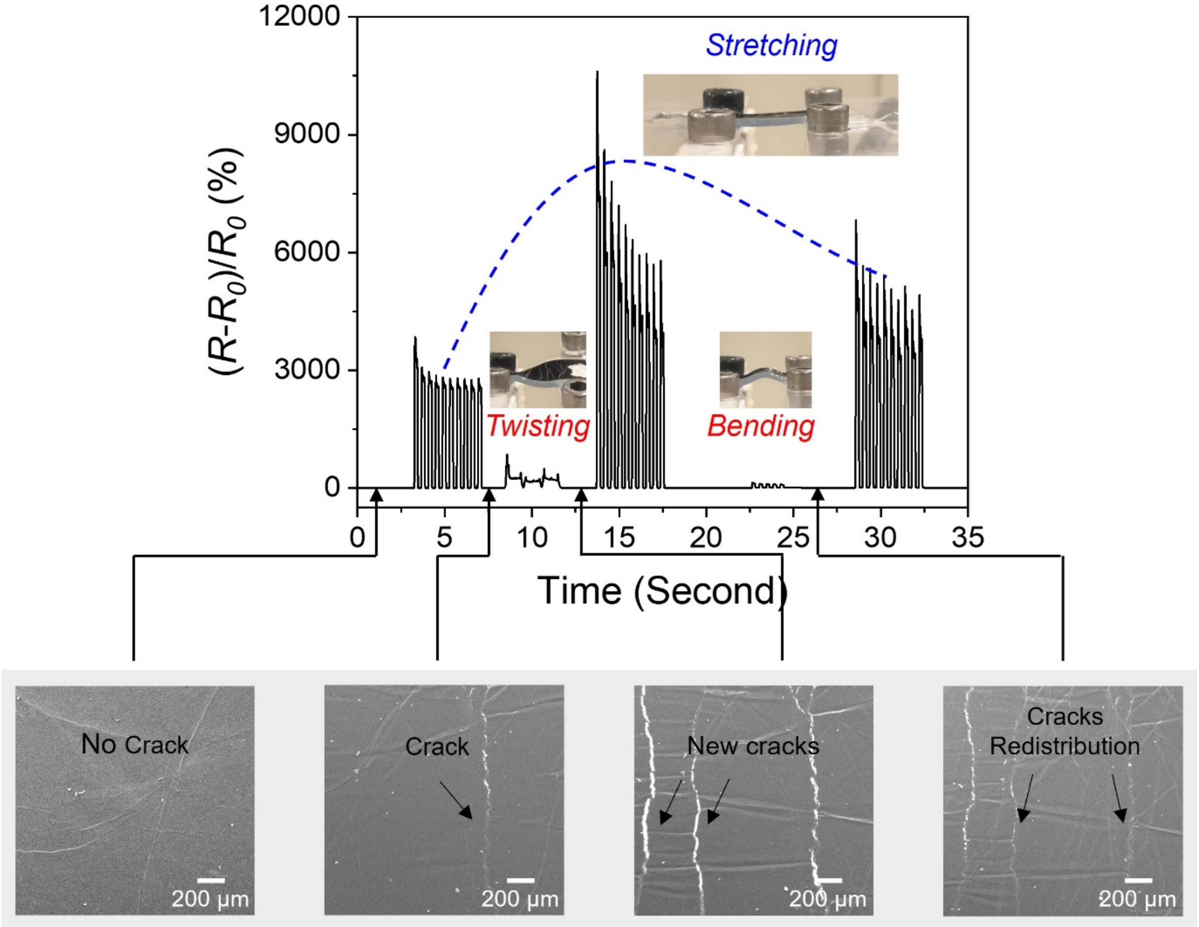

Supplementary Fig. 14. Sensing profile and SEM images of a planar sensor under a stretching, twisting, stretching, bending, stretching mechanical loading. The stretching strain was 10%.

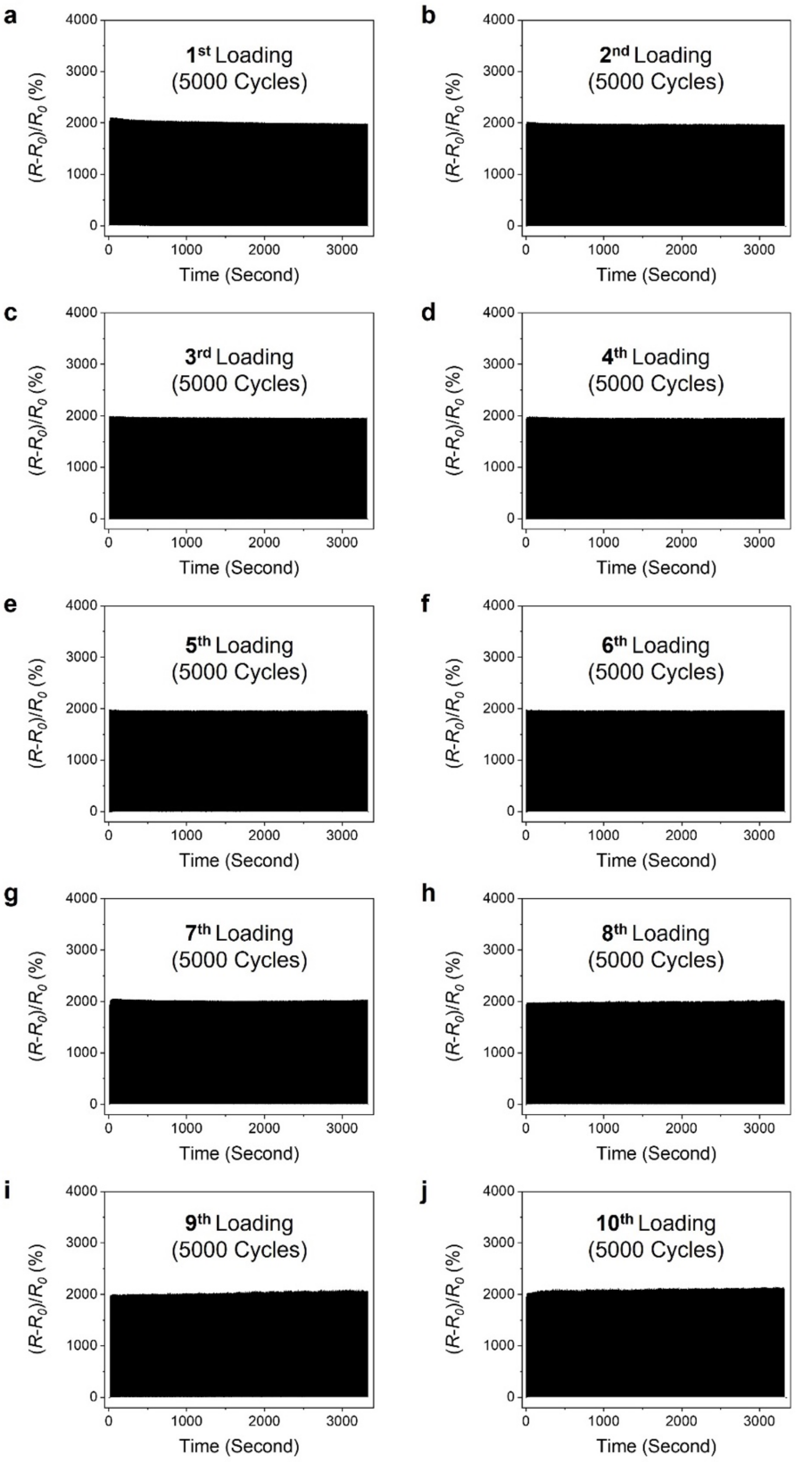

Supplementary Fig. 15. Sensing profiles of a PCAM sensor under intermittent cyclic loading (1-10 cyclic rounds). Each cyclic loading consisted of 5,000 cycles of uniaxial stretching at 85% strains, with a 1-hour time interval between each cycle. (**a**) Sensing profile of 1st cyclic loading. (**b**) Sensing profile of 2nd cyclic loading. (**c**) Sensing profile of 3rd cyclic loading. (**d**) Sensing profile of 4th cyclic loading. (**e**) Sensing profile of 5th cyclic loading. (**f**) Sensing profile of 6th cyclic loading. (**g**) Sensing profile of 7th cyclic loading. (**h**) Sensing profile of 8th cyclic loading. (**i**) Sensing profile of 9th cyclic loading. (**j**) Sensing profile of 10th cyclic loading.

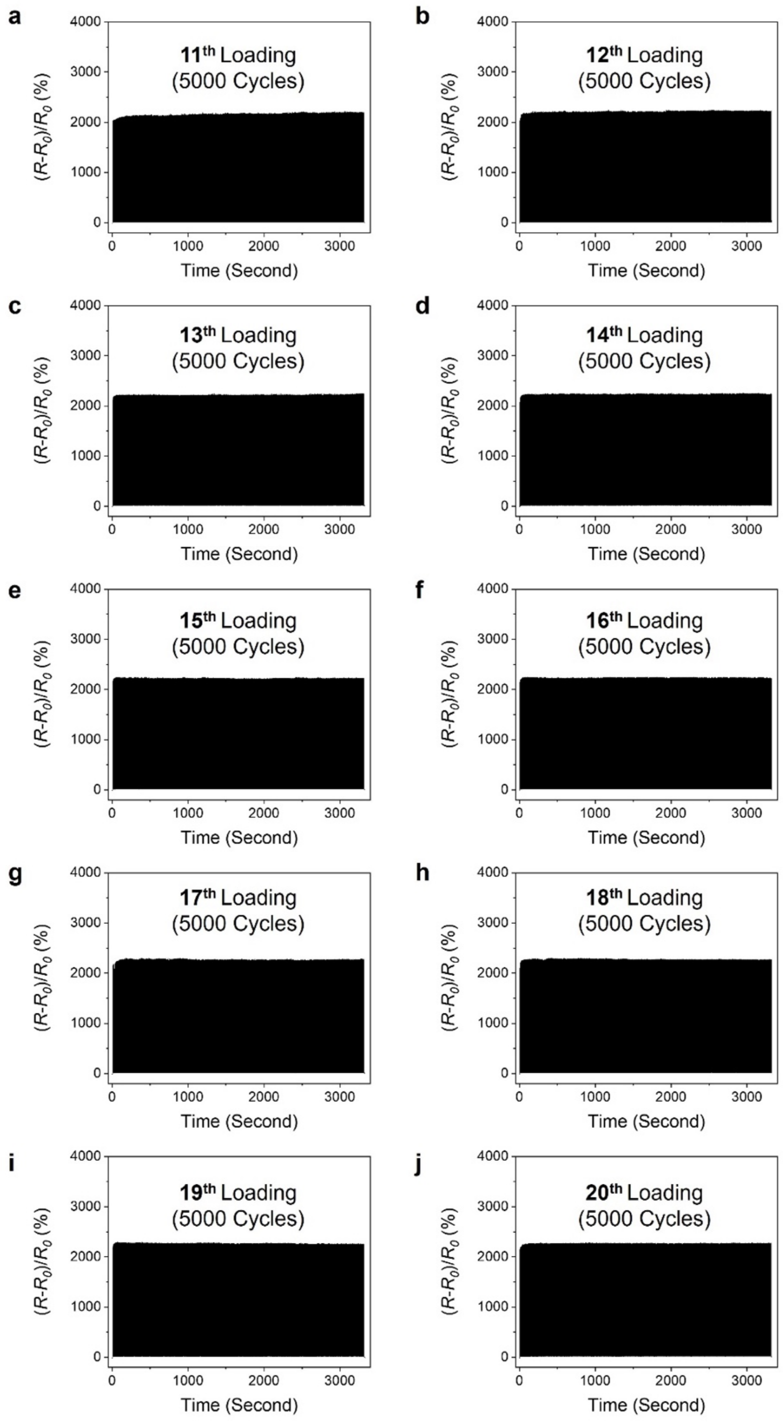

Supplementary Fig. 16. Sensing profiles of a PCAM sensor under intermittent cyclic loading (11-20 cyclic rounds). Each cyclic loading consisted of 5,000 cycles of uniaxial stretching at 85% strains, with a 1-hour time interval between each cycle. (**a**) Sensing profile of 11th cyclic loading. (**b**) Sensing profile of 12th cyclic loading. (**c**) Sensing profile of 13th cyclic loading. (**d**) Sensing profile of 14th cyclic loading. (**e**) Sensing profile of 15th cyclic loading. (**f**) Sensing profile of 16th cyclic loading. (**g**) Sensing profile of 17th cyclic loading. (**h**) Sensing profile of 18th cyclic loading. (**i**) Sensing profile of 19th cyclic loading. (**j**) Sensing profile of 20th cyclic loading.

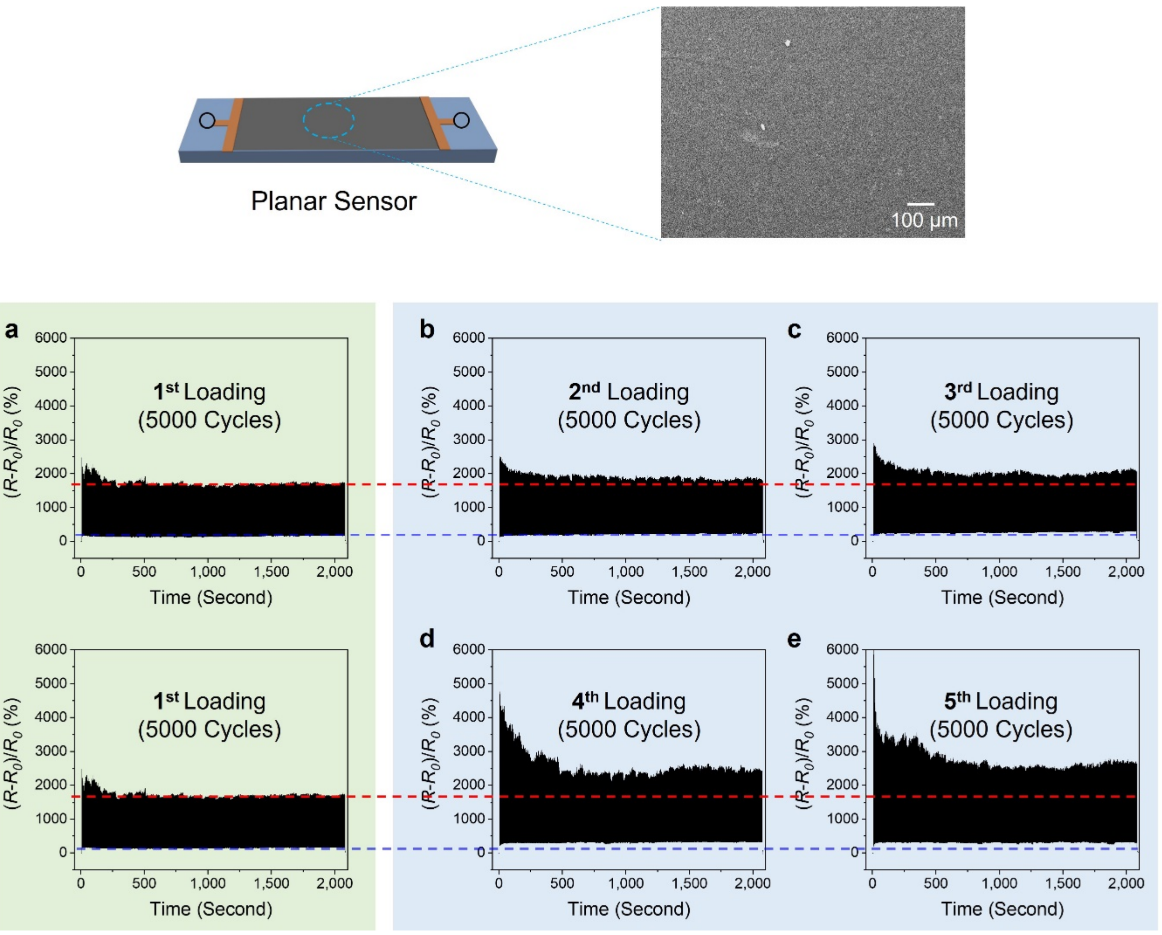

Supplementary Fig. 17. Sensing profiles of a planar sensor under intermittent cyclic loading. Each cyclic loading consisted of 5,000 cycles of uniaxial stretching at 85% strains, with a 1-hour time interval between each cycle. (**a**) Sensing profile of 1st cyclic loading. (**b**) Sensing profile of 2nd cyclic loading. (**c**) Sensing profile of 3rd cyclic loading. (**d**) Sensing profile of 4th cyclic loading. (**e**) Sensing profile of 5th cyclic loading.

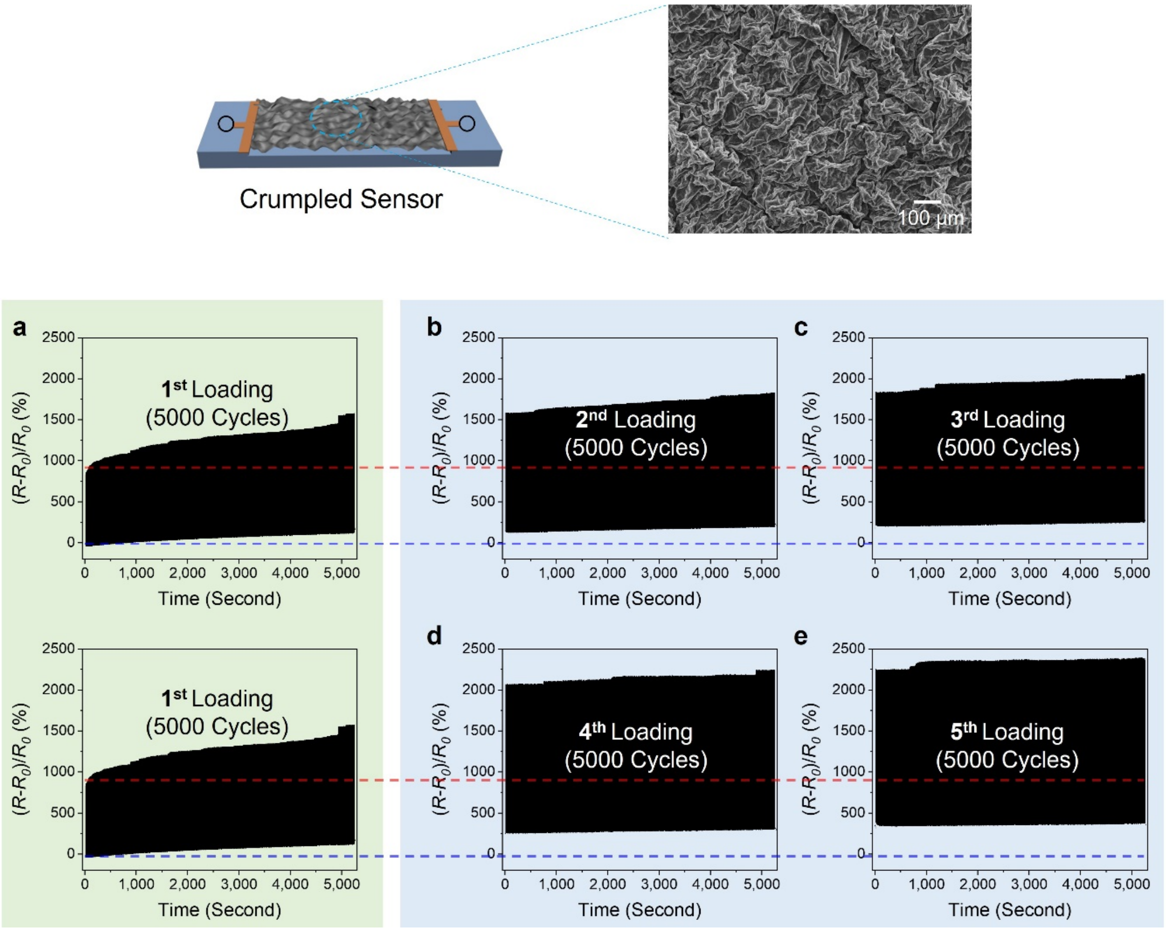

Supplementary Fig. 18. Sensing profiles of a crumpled sensor under intermittent cyclic loading. Each cyclic loading consisted of 5,000 cycles of uniaxial stretching at 85% strains, with a 1-hour time interval between each cycle. (**a**) Sensing profile of 1st cyclic loading. (**b**) Sensing profile of 2nd cyclic loading. (**c**) Sensing profile of 3rd cyclic loading. (**d**) Sensing profile of 4th cyclic loading. (**e**) Sensing profile of 5th cyclic loading.

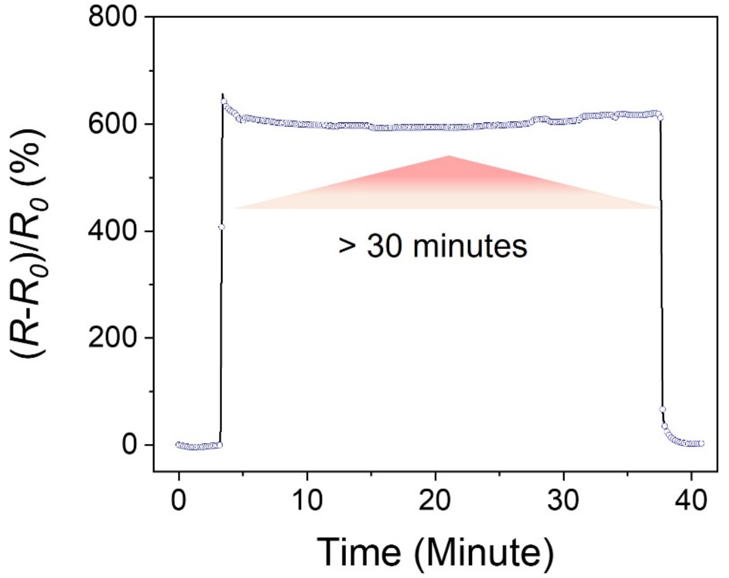

Supplementary Fig. 19. Sensing profile of a PCAM sensor (ρ=1,200 µm mm^{-2}; φ=40%) under a static stretching state (30% strains) over 30 minutes.

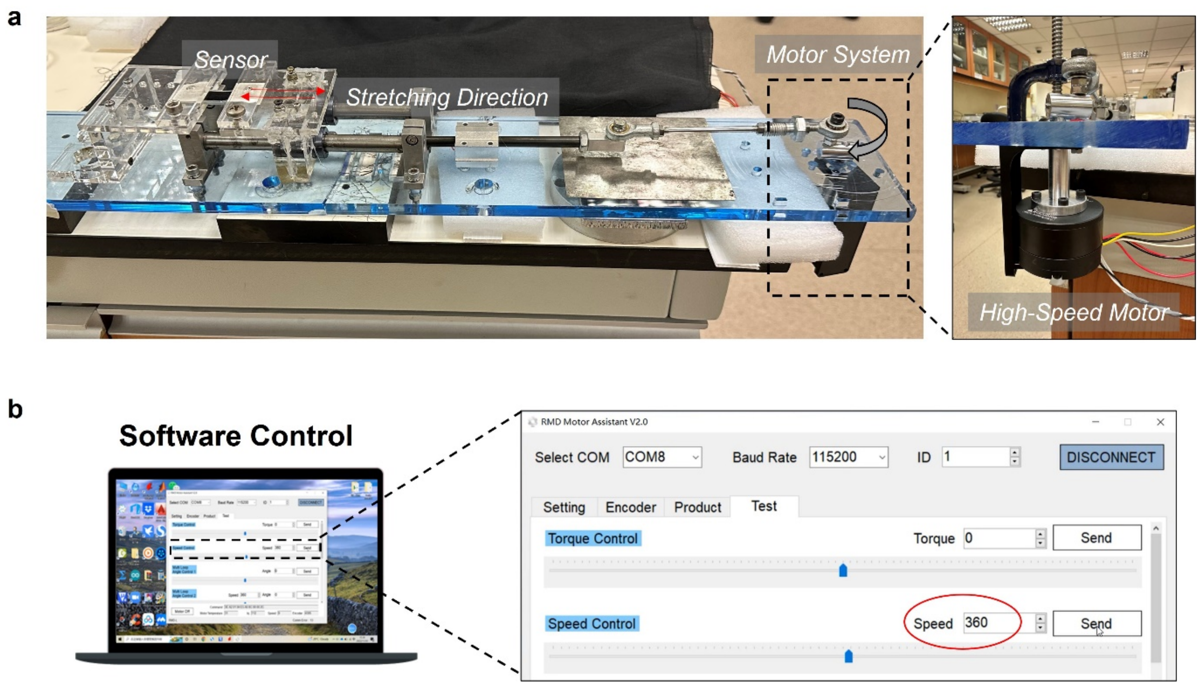

Supplementary Fig. 20. Setups to test sensor stability under dynamic stretching frequencies. (**a**) A home-made machine used to generate cyclic, linear motions in high frequency. (**b**) The stretching frequency of motor could be controlled by a software (RAM Motor Assistant V2.0).

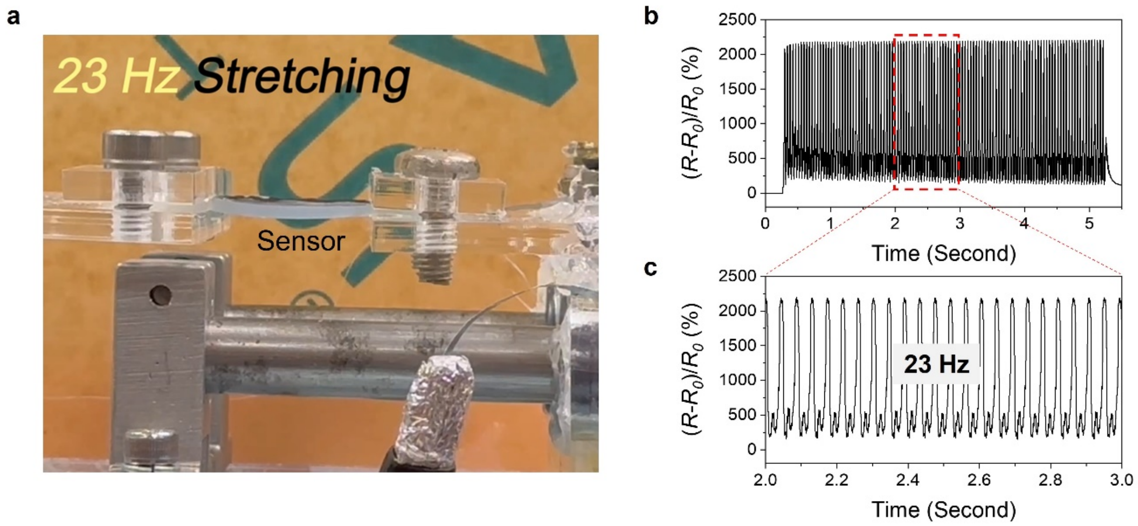

Supplementary Fig. 21. Sensor stability under 23 Hz stretching frequency. (**a**) Machine setup used to generate a 23 Hz high frequency. (**b**) (**c**) Sensing profile of a PCAM sensor (ρ=1,200 µm mm^{-2}; φ=40%) under 23 Hz stretching frequency.

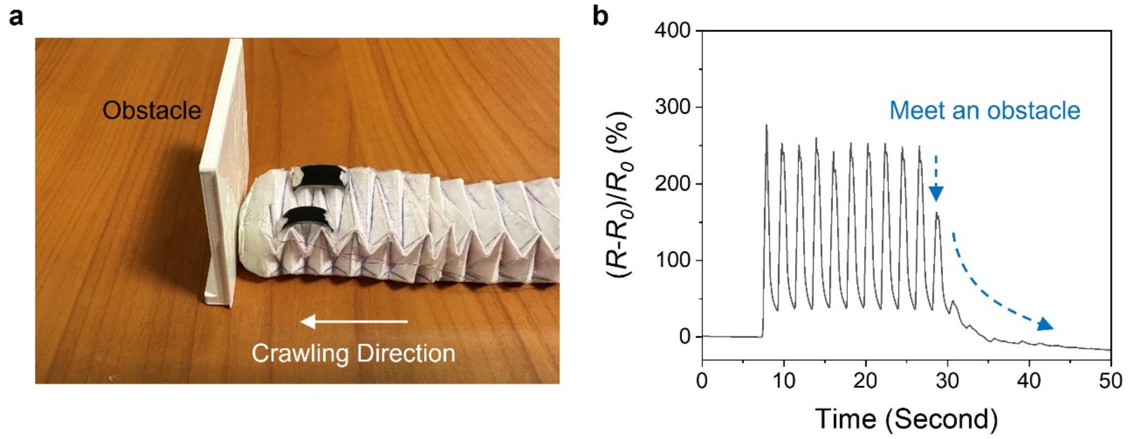

Supplementary Fig. 22. Digital photo (a) and sensing profile (b) of sensor-integrated origami robot when it was meeting an obstacle.

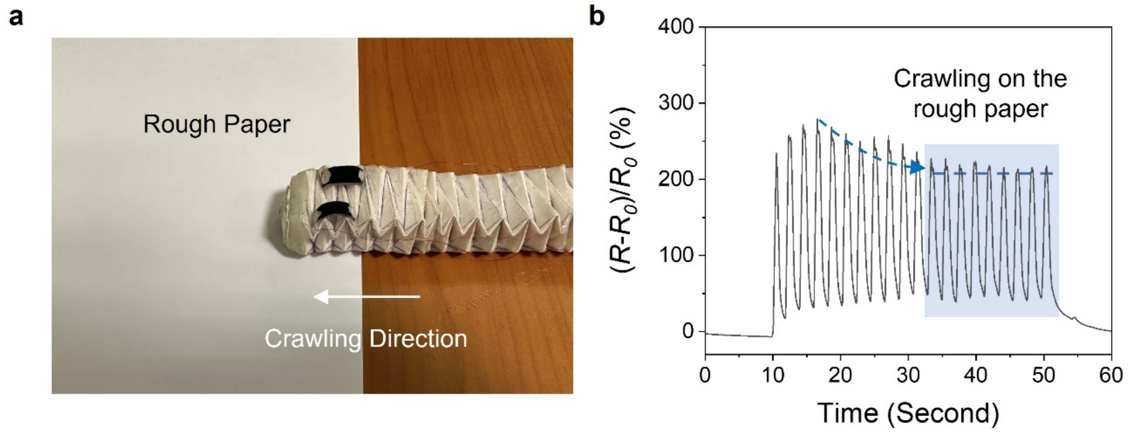

Supplementary Fig. 23. Digital photo (a) and sensing profile (b) of sensor-integrated origami robot when it was crawling from desktop to a rough paper.

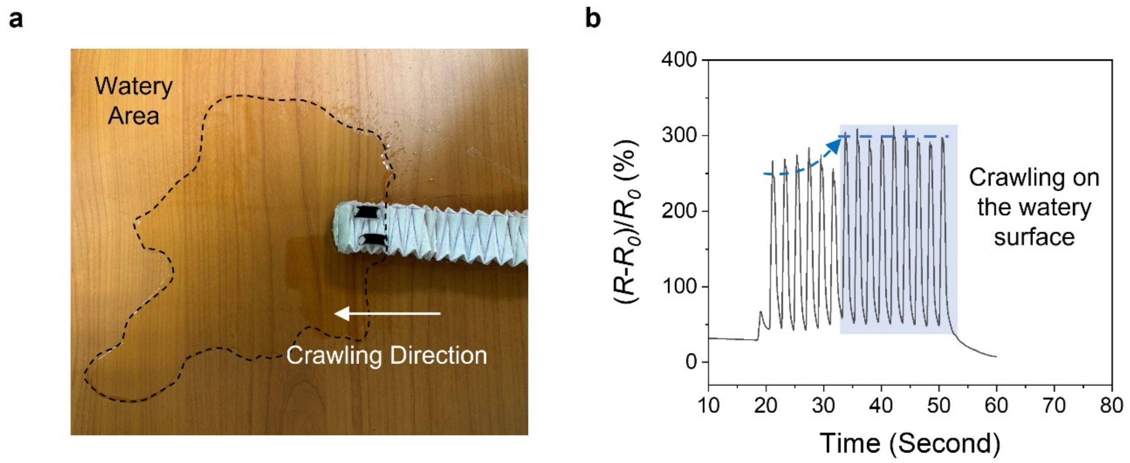

Supplementary Fig. 24. Digital photo (a) and sensing profile (b) of sensor-integrated origami robot when it was crawling from desktop to a watery surface.

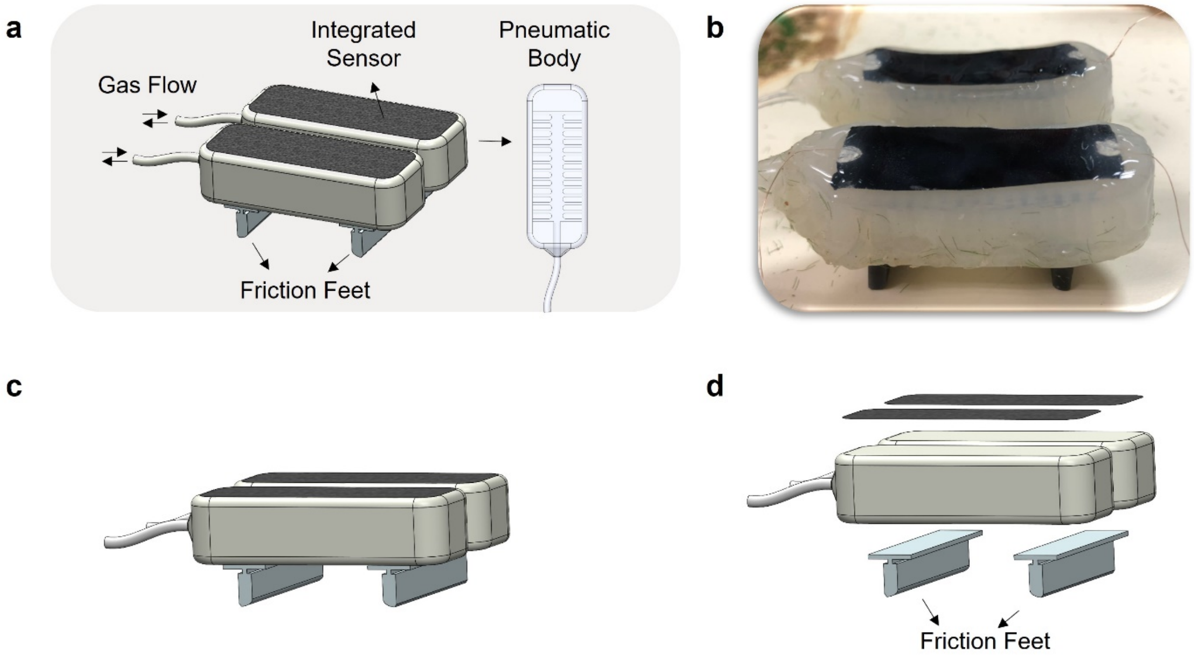

Supplementary Fig. 25. Fabrication and sensor integration of a pneumatic robot. (**a**) structure design of the pneumatic soft robot. (**b**) Digital photo of the as-fabricated pneumatic soft robot. (**c**) Cross-section view of the pneumatic soft robot. (**d**) Exploded-view drawing of the pneumatic soft robot.

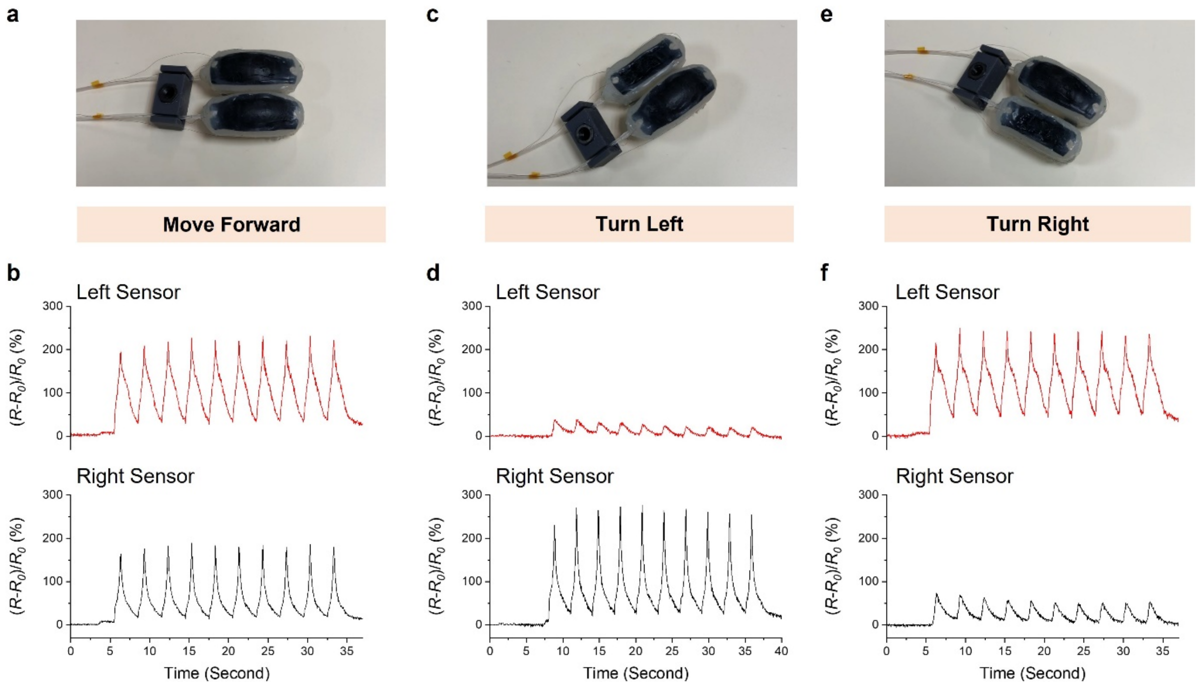

Supplementary Fig. 26. Digital photos and sensing profiles of a PCAM sensor-integrated pneumatic soft robot when it was moving forward (a, b), turning left (c, d), and turning right (e, f). All robot movements were implemented under the control of pneumatic actuation system (see details in **Methods**).

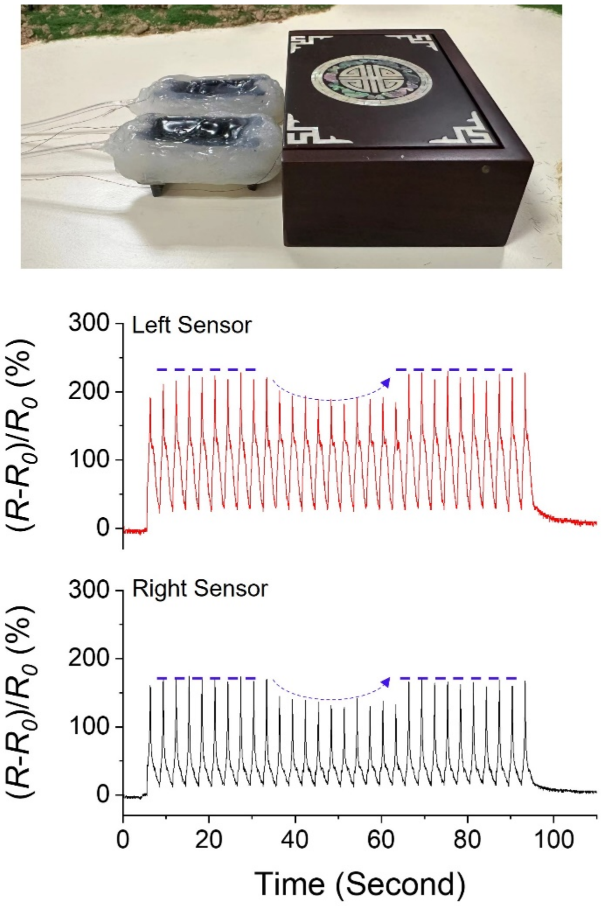

Supplementary Fig. 27. Digital photos and sensing profiles of a PCAM sensor-integrated pneumatic soft robot when it was meeting an obstacle.

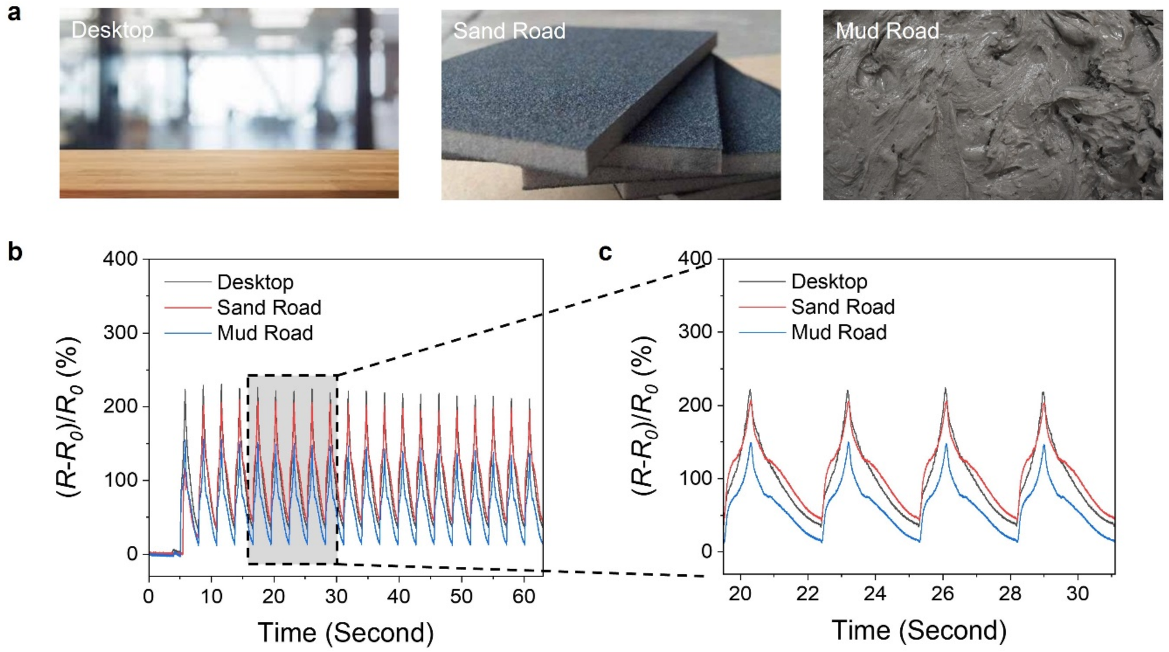

Supplementary Fig. 28. Digital photos and sensing profiles of a PCAM sensor-integrated pneumatic soft robot when it was crawling on different friction surfaces, including desktop, sand road, and mud road. (**a**) Digital photos of different friction surfaces. (**b**) Sensing profiles of a PCAM sensor-integrated robot when it was crawling on different friction surfaces. (**c**) Amplified sensing signals in figure (**b**).

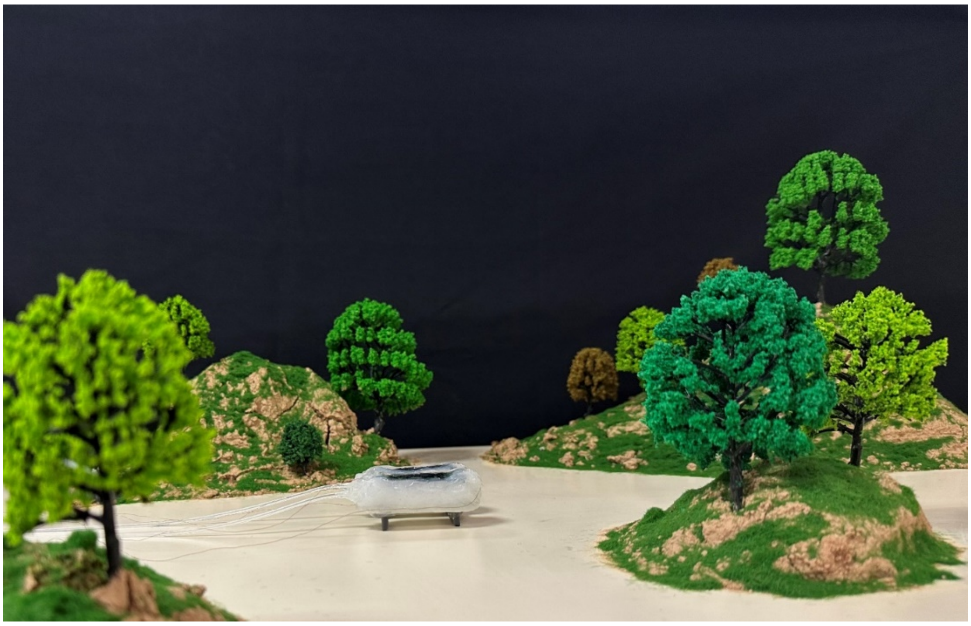

Supplementary Fig. 29. Digital photo of a pneumatic soft robot in an artificial terrain.

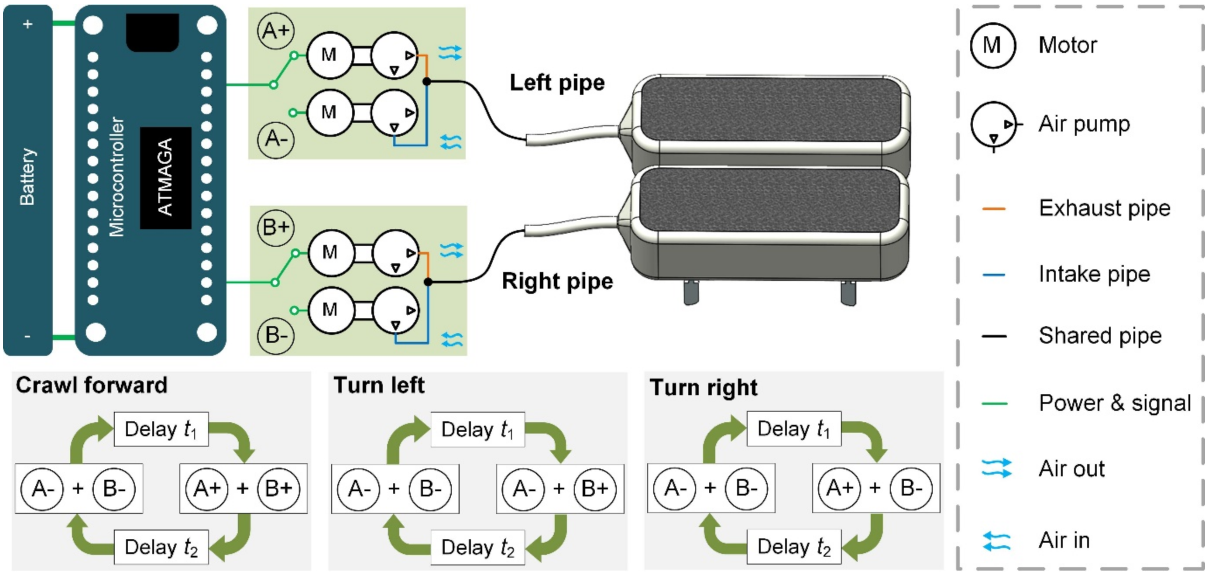

Supplementary Fig. 30. Actuation system design of the pneumatic soft robot. This figure described the actuation and control system of the proposed robot. Robot bodies are driven by two customized pneumatic units (A and B) composed of motors and air pumps. Each unit can exhaust or inhale under the control of the microcontroller. When one unit exhausts (represented by A+, B+) or inhales (represented by A-, B-), the corresponding robot body(s) extends or flexes. The crawling and turning motions are completed by different combinations of the units' inhalation and exhaust. The time delay t_1 and t_2 determines the speed of the motions.

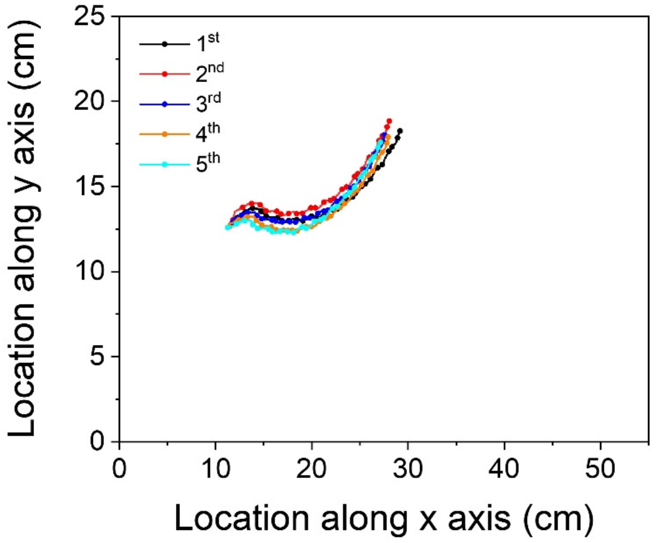

Supplementary Fig. 31. Position tracking of the pneumatic soft under trajectory 1, which was repeated 5 times.

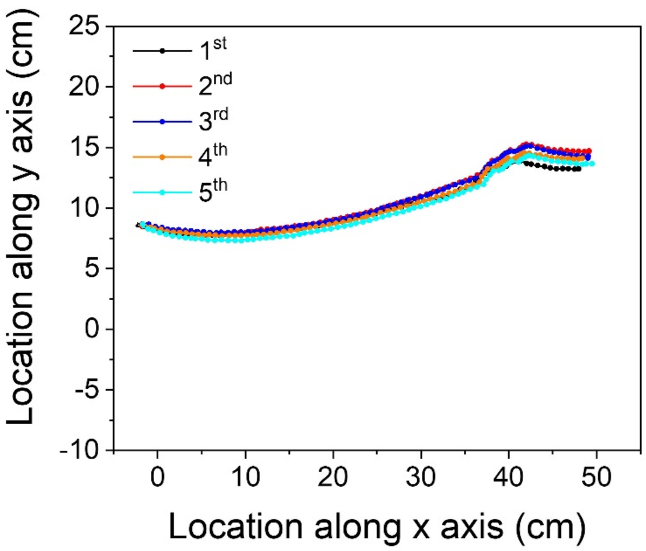

Supplementary Fig. 32. Position tracking of the pneumatic soft under trajectory 2, which was repeated 5 times.

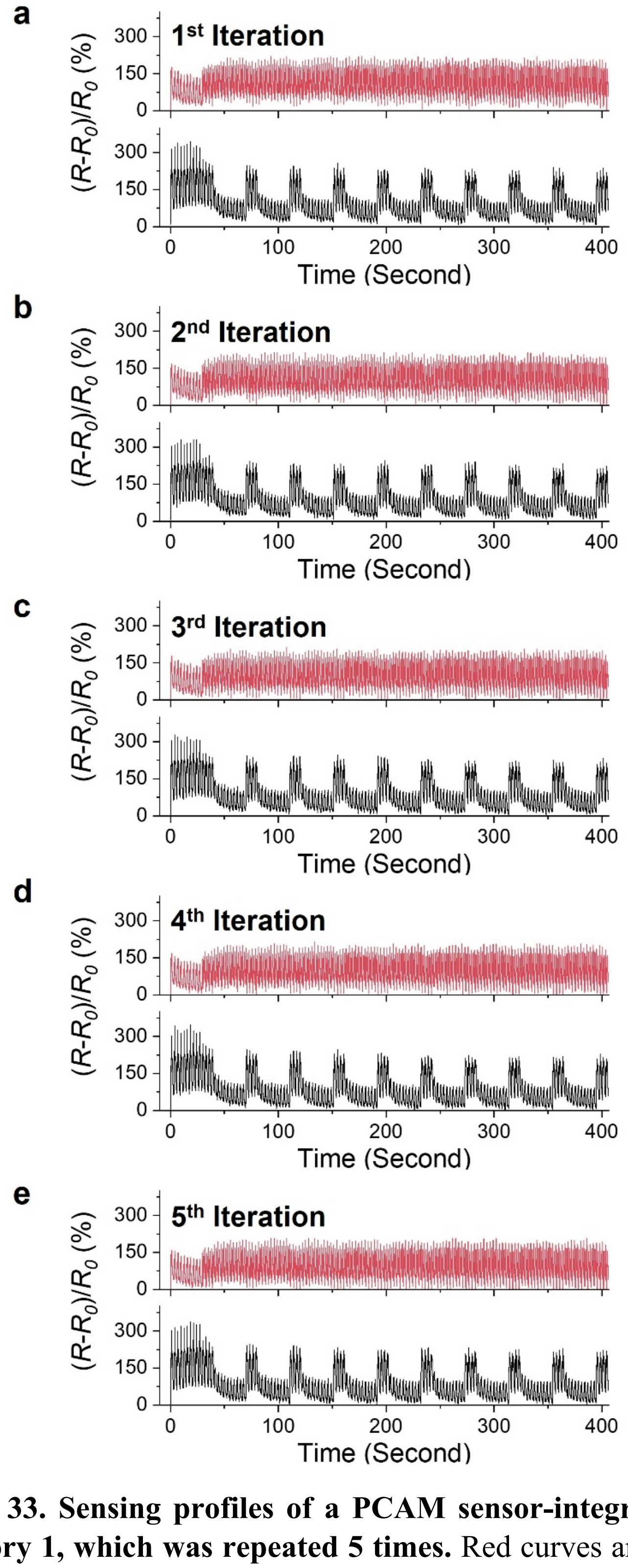

Supplementary Fig. 33. Sensing profiles of a PCAM sensor-integrated pneumatic soft robot under trajectory 1, which was repeated 5 times. Red curves are sensor signals from right robot body and the black curves are sensor signals from left robot body. (**a**) Sensing profile of 1st iteration. (**b**) Sensing profile of 2nd iteration. (**c**) Sensing profile of 3rd iteration. (**d**) Sensing profile of 4th iteration. (**e**) Sensing profile of 5th iteration.

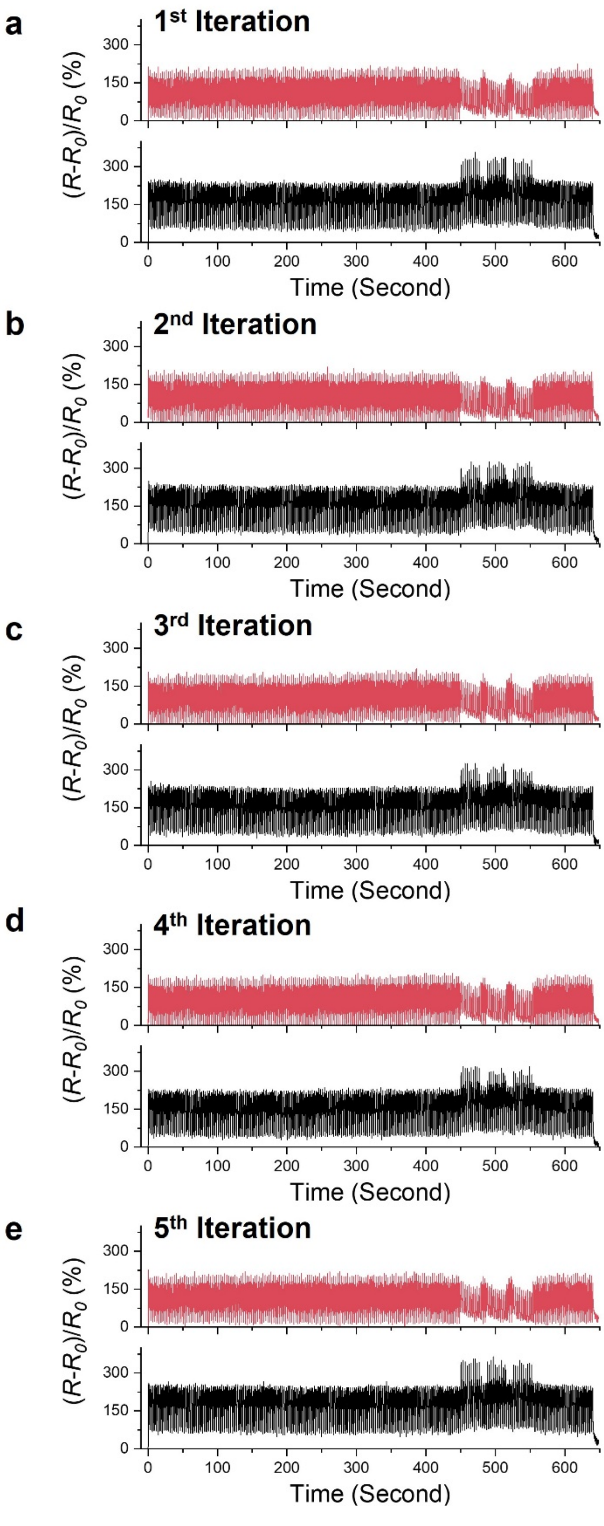

Supplementary Fig. 34. Sensing profiles of a PCAM sensor-integrated pneumatic soft robot under trajectory 2, which was repeated 5 times. Red curves are sensor signals from right robot body and the black curves are sensor signals from left robot body. (**a**) Sensing profile of 1st iteration. (**b**) Sensing profile of 2nd iteration. (**c**) Sensing profile of 3rd iteration. (**d**) Sensing profile of 4th iteration. (**e**) Sensing profile of 5th iteration.

a

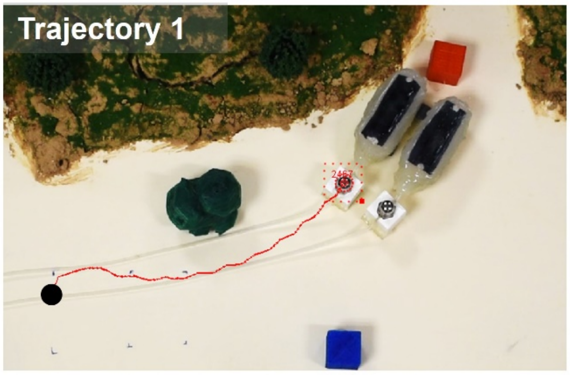

b

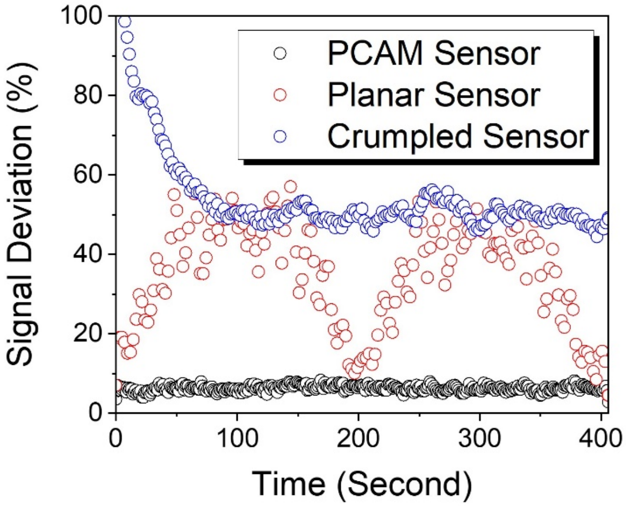

Supplementary Fig. 35. Sensing performance of a PCAM sensor-integrated pneumatic soft robot under trajectory 1, which was repeated 5 times. (**a**) Digital photo of robot trajectory 1. (**b**) Comparison of signal deviations of pneumatic soft robots, which integrated PCAM sensor, planar sensor, and crumpled sensor, respectively. Herein, the signal deviations were calculated based on the sensing profiles between 1st and 5th iterations under trajectory 1. Detailed calculation method was provided in Equation 6 in main manuscript.

a

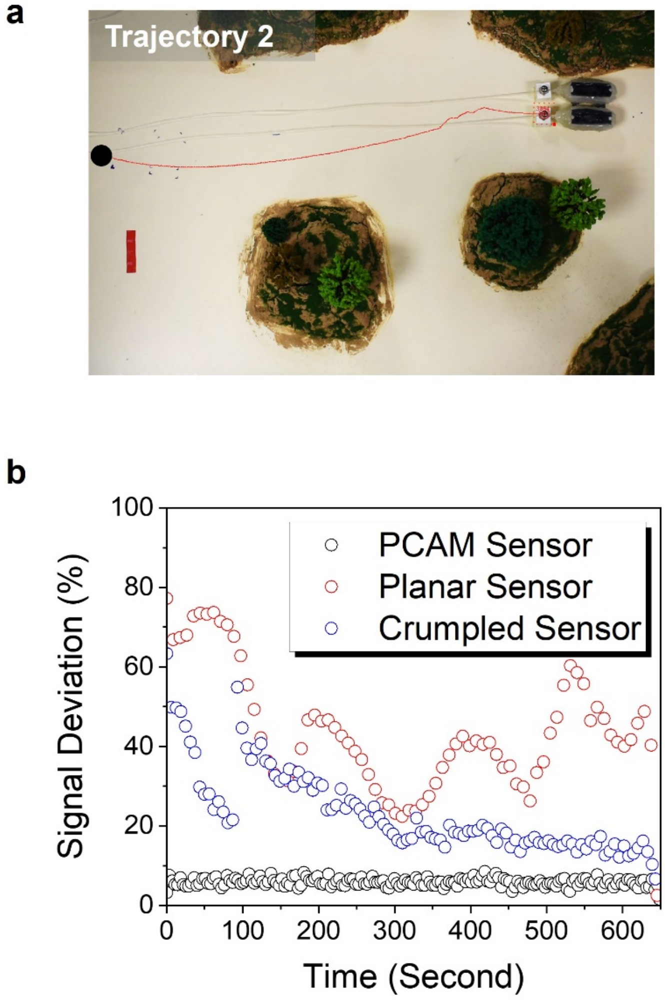

Supplementary Fig. 36. Sensing performance of a PCAM sensor-integrated pneumatic soft robot under trajectory 2, which was repeated 5 times. (**a**) Digital photo of robot trajectory 2. (**b**) Comparison of signal deviations of pneumatic soft robots, which integrated PCAM sensor, planar sensor, and crumpled sensor, respectively. Herein, the signal deviations were calculated based on the sensing profiles between 1st and 5th iterations under trajectory 2. Detailed calculation method was provided in Equation 6 main manuscript.

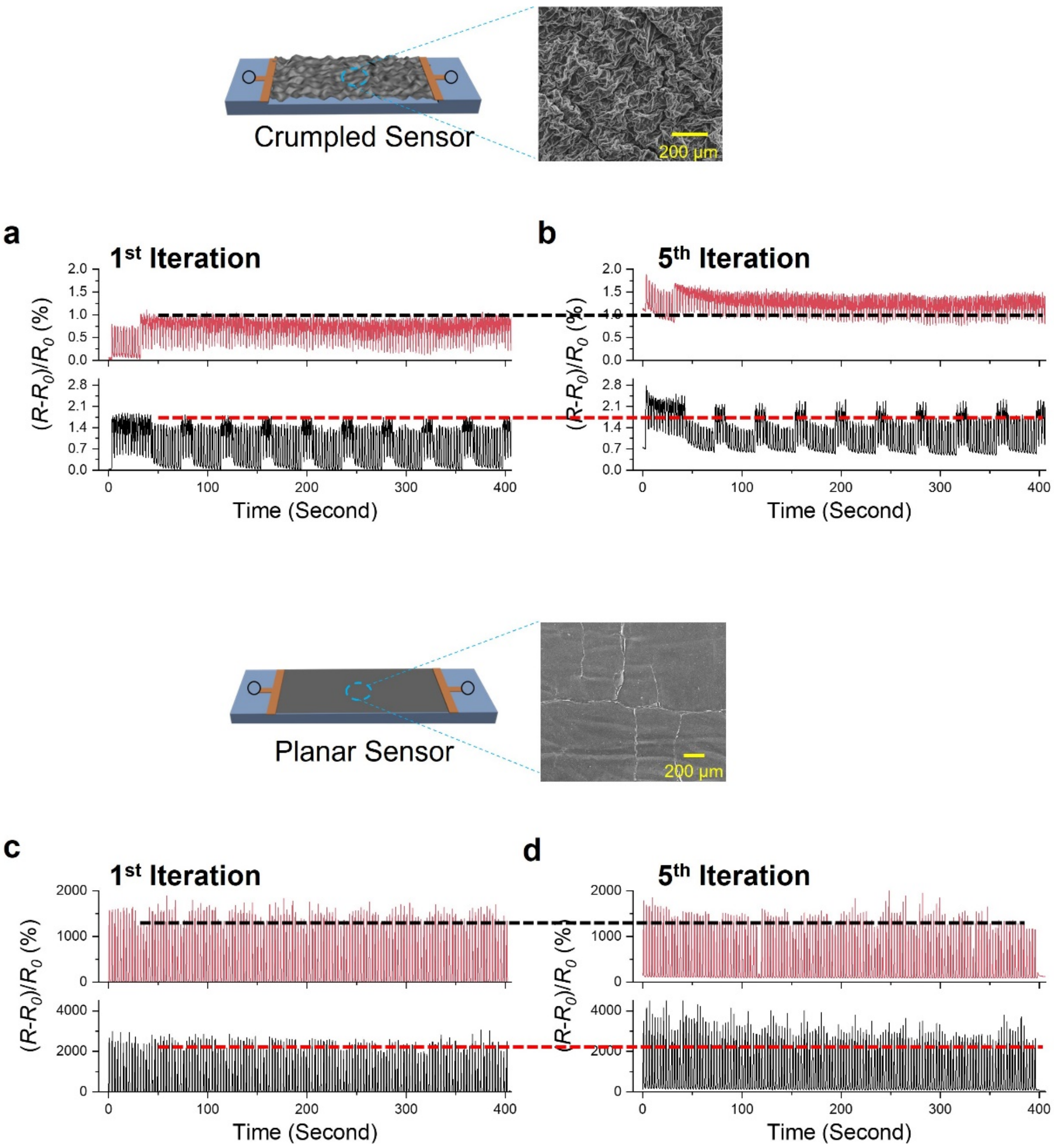

Supplementary Fig. 37. Sensing profiles of a planar and crumpled sensor-integrated pneumatic soft robot under trajectory 1, which was repeated 5 times. (**a**) Sensing profile of a crumpled sensor-integrated pneumatic soft robot in the 1st iteration of trajectory 1. (**b**) Sensing profile of a crumpled sensor-integrated pneumatic soft robot in the 5th iteration of trajectory 1. (**c**) Sensing profile of a planar sensor-integrated pneumatic soft robot in the 1st iteration of trajectory 1. (**d**) Sensing profile of a planar sensor-integrated pneumatic soft robot in the 5th iteration of trajectory 1. Red curves are sensor signals from right robot body and the black curves are sensor signals from left robot body.

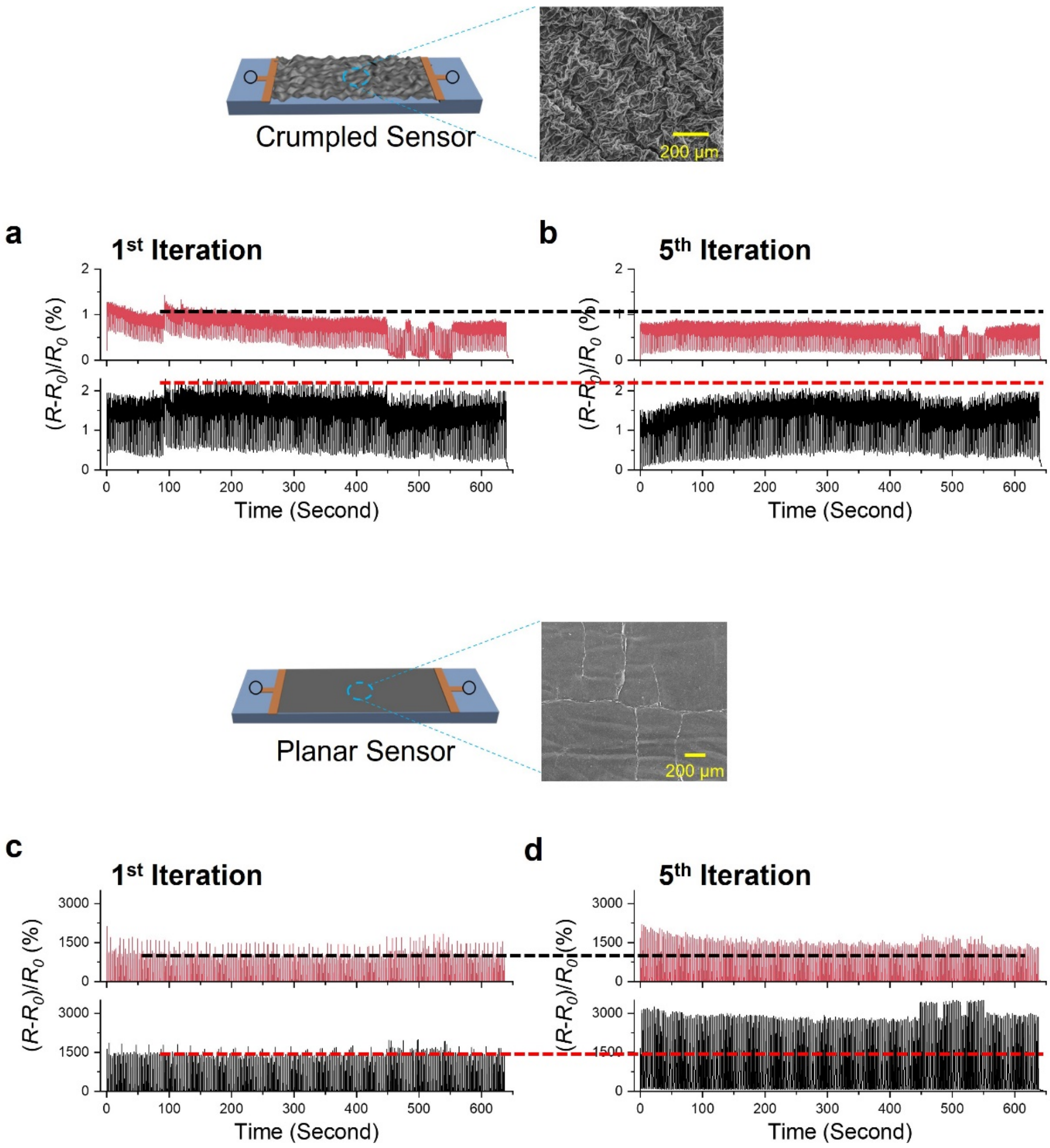

Supplementary Fig. 38. Sensing profiles of a planar and crumpled sensor-integrated pneumatic soft robot under trajectory 2, which was repeated 5 times. (**a**) Sensing profile of a crumpled sensor-integrated pneumatic soft robot in the 1st iteration of trajectory 2. (**b**) Sensing profile of a crumpled sensor-integrated pneumatic soft robot in the 5th iteration of trajectory 2. (**c**) Sensing profile of a planar sensor-integrated pneumatic soft robot in the 1st iteration of trajectory 2. (**d**) Sensing profile of a planar sensor-integrated pneumatic soft robot in the 5th iteration of trajectory 2. Red curves are sensor signals from right robot body and the black curves are sensor signals from left robot body.

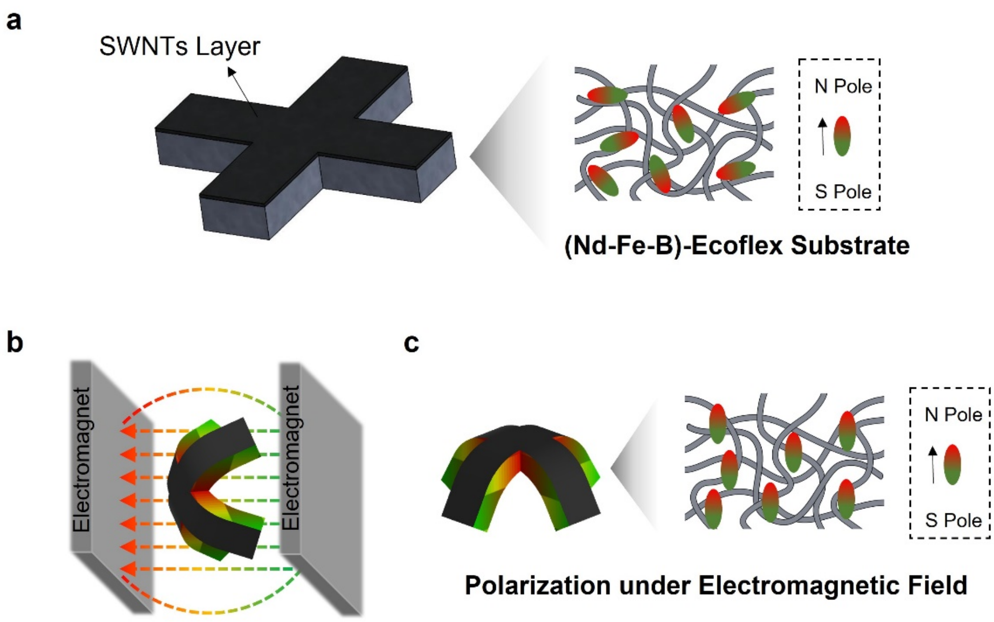

Supplementary Fig. 39. Fabrication of the tetrapod microrobot. (**a**) Structure design of the tetrapod microrobot. (**b**) The tetrapod microrobot was put into the space between two electromagnets with high field uniformity (at 1.2 T; EM4-HVA-S, Lake Shore Cryotronics), which induced the magnetic orientations to the tetrapods (**c**).

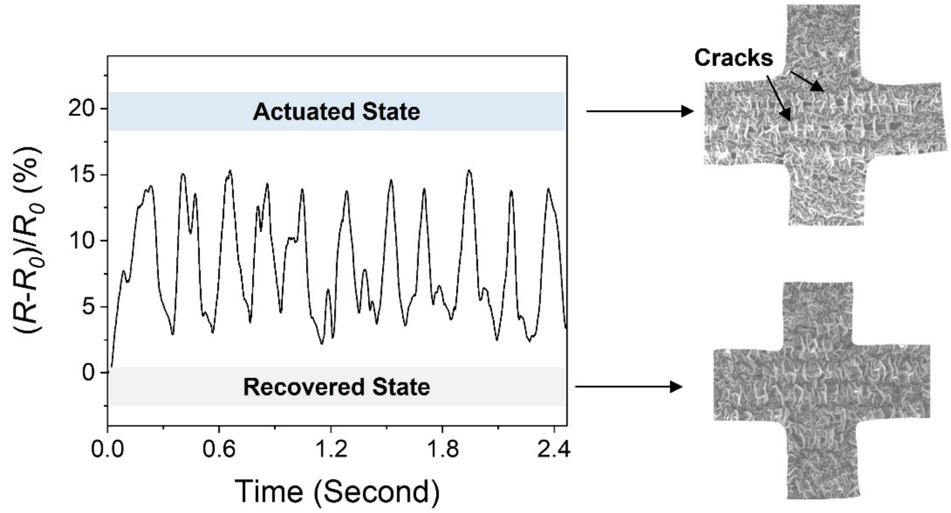

Supplementary Fig. 40. Sensing profile of a tetrapod microrobot under repeated magnet actuation. The SEM images illustrated that the robot surface crack grew under the actuated state, thus leading to higher resistance changes.

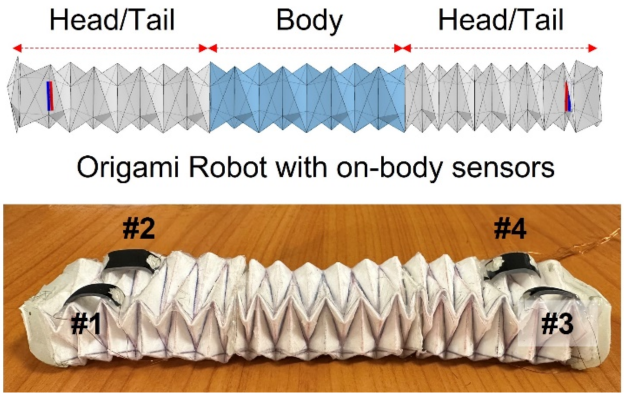

Supplementary Fig. 41. Origami robot with four on-body sensors.

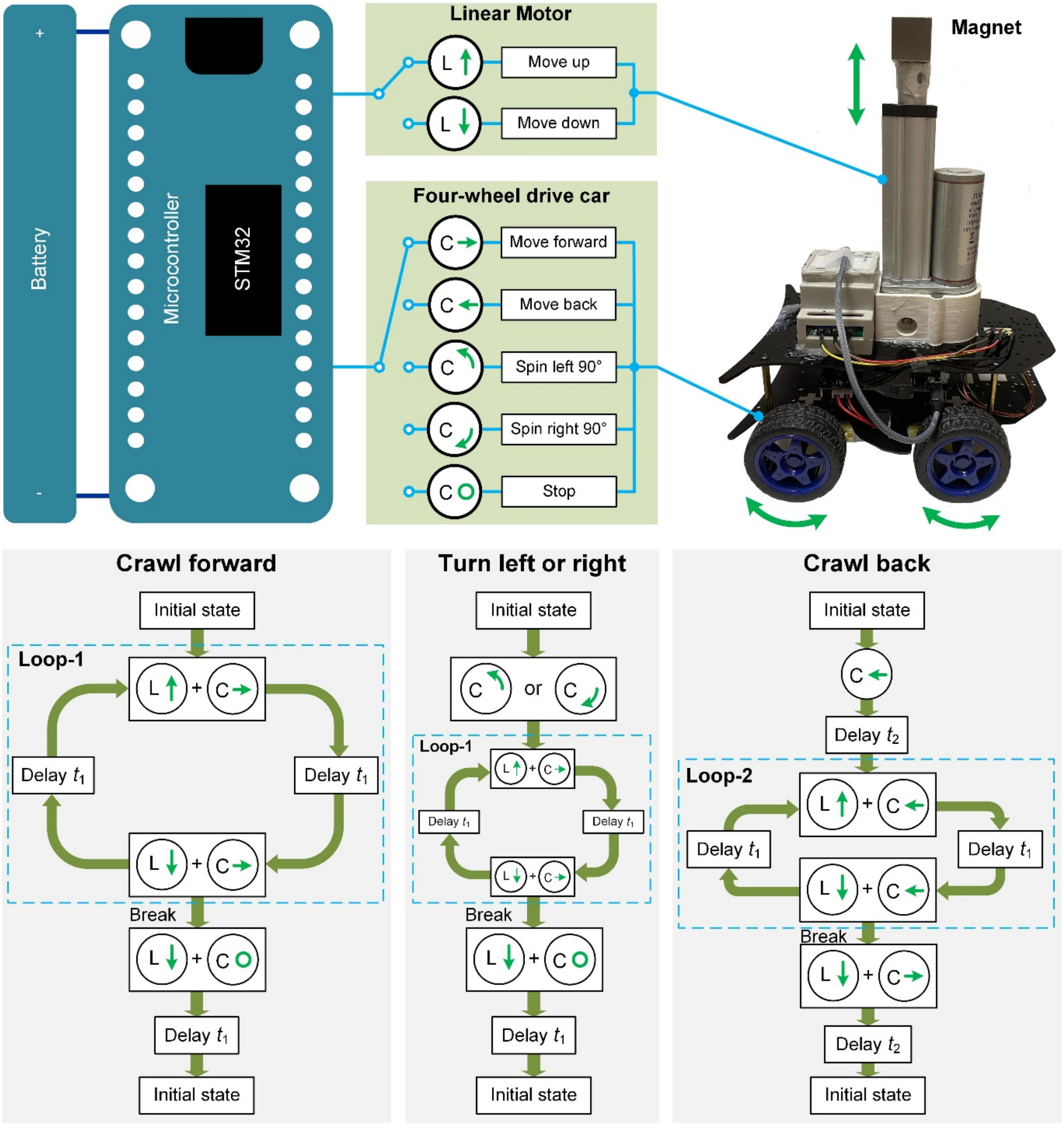

Supplementary Fig. 42. Actuation system design of the origami robot. This figure describes the mobile robot platform for driving the proposed origami robot. The platform consists of a linear motor and a four-wheel drive car. A magnet is placed on top of the linear motor. All motions of the robot start from an initial state when the platform is right below the robot head and the magnet is at the lowest position. The crawl forward motion is achieved by loop-1 in which the linear motor moves the magnet up and down and the car keeps moving forward. When adding a car spinning left or right movement before crawling forward, the robot can turn to left or right. The crawl back motion is done by first moving the magnet to the position of the tail of the robot, then going into loop-2 to move the magnet back while pushing it up and down. The parameter t_1 determines the motion cycle of the robot and t_2 is decided by the robot length.

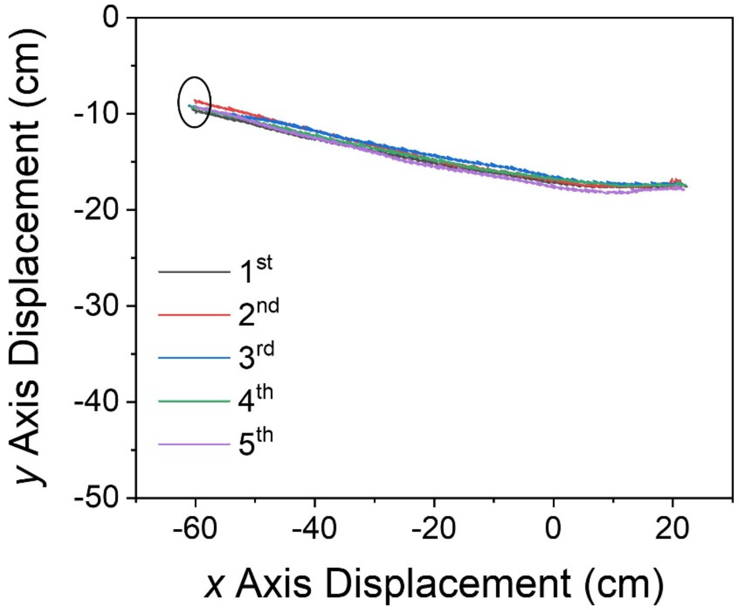

Supplementary Fig. 43. Position tracking of the origami robot under repeated 5 iterations of navigation. After 5 times repeat of 112 cm-long robot navigation, the robot trajectories were highly consistent and the average distance among 5 destinations was only 0.8 cm, indicating <1% system error.

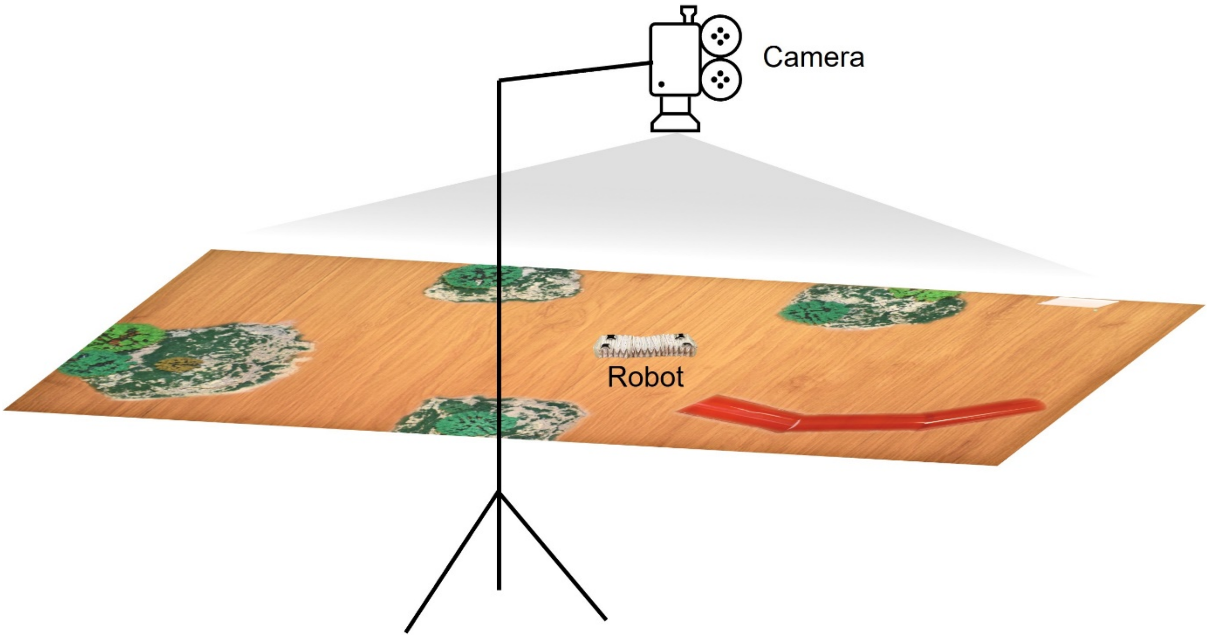

Supplementary Fig. 44. Robot location/trajectory tracking by a camera system.

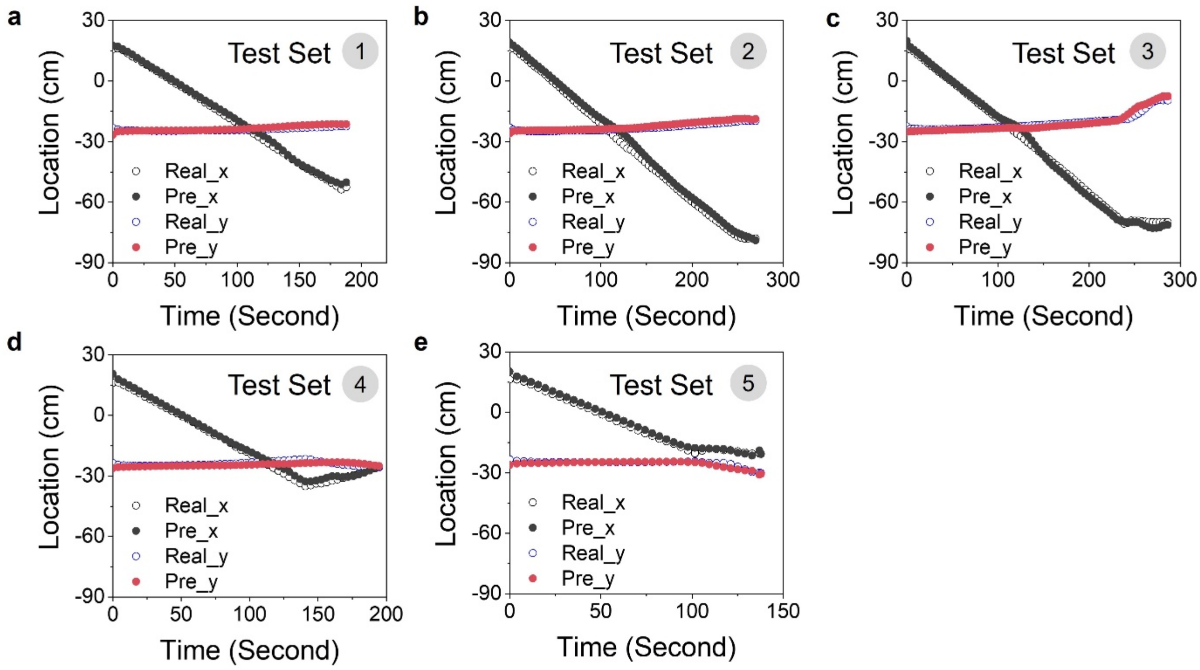

Supplementary Fig. 45. Performance of the trajectory prediction model. Based on the test sets of ①, ②, ③, ④, ⑤, the ANN model showed low RE and AE. (**a**) Prediction performance of the ANN model on 1st test set. (**b**) Prediction performance of the ANN model on 2nd test set. (**c**) Prediction performance of the ANN model on 3rd test set. (**d**) Prediction performance of the ANN model on 4th test set. (**e**) Prediction performance of the ANN model on 5th test set.

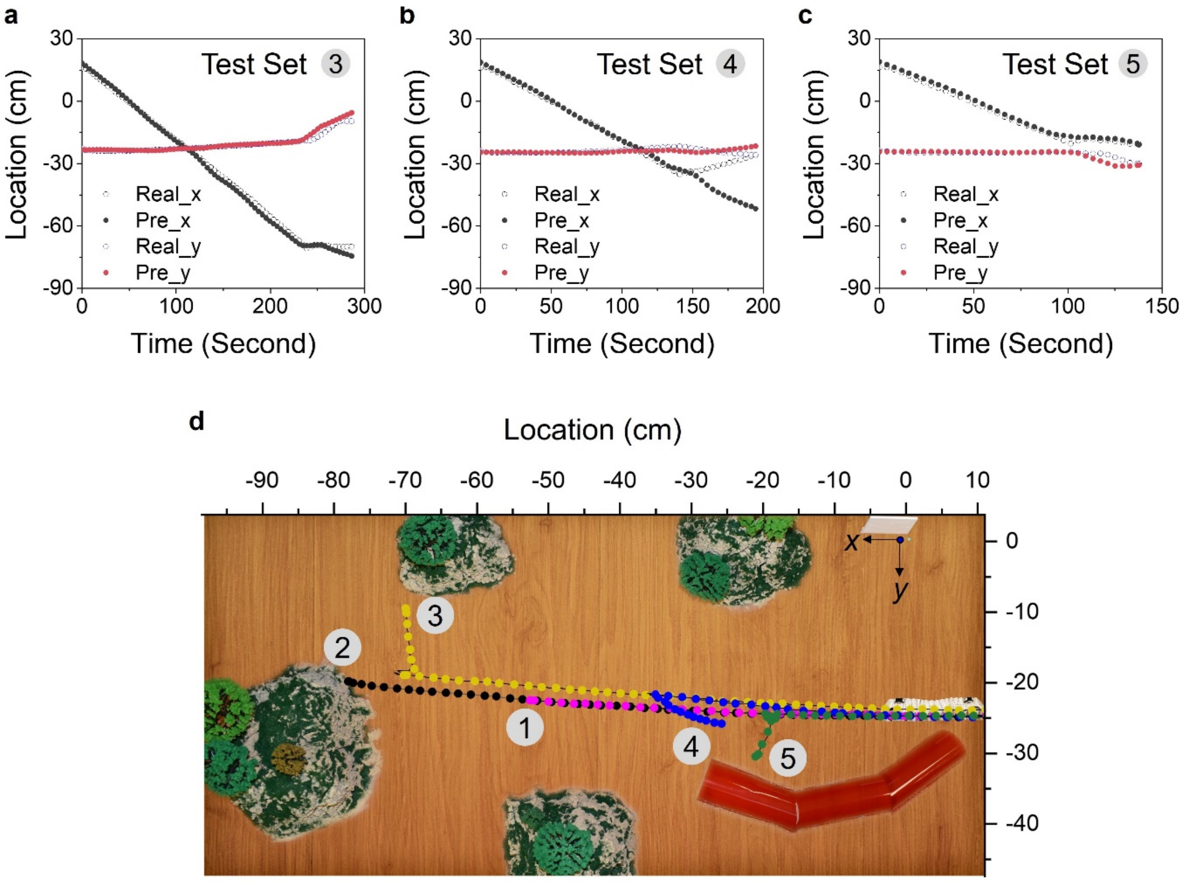

Supplementary Fig. 46. Prediction performance of an extra ANN model by using only robot actuation information as the training data. Based on the test sets of ③, ④, ⑤, the ANN model showed higher average RE and AE of 12.1% and 16.2 cm, respectively. (**a**) Prediction performance of the ANN model on 3rd test set. (**b**) Prediction performance of the ANN model on 4th test set. (**c**) Prediction performance of the ANN model on 5th test set. (**d**) Records of robot trajectories from test sets.

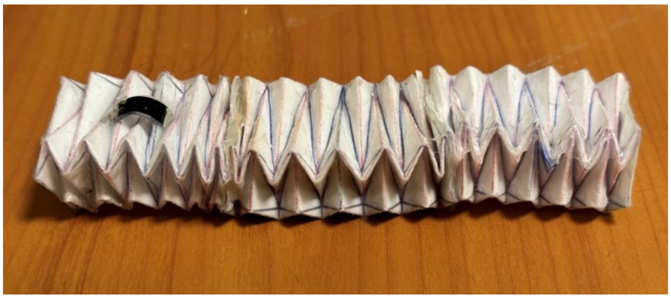

Supplementary Fig. 47. One PCAM sensor was attached at the central of robot head to realize the sensing capability of terrain altitude changes.

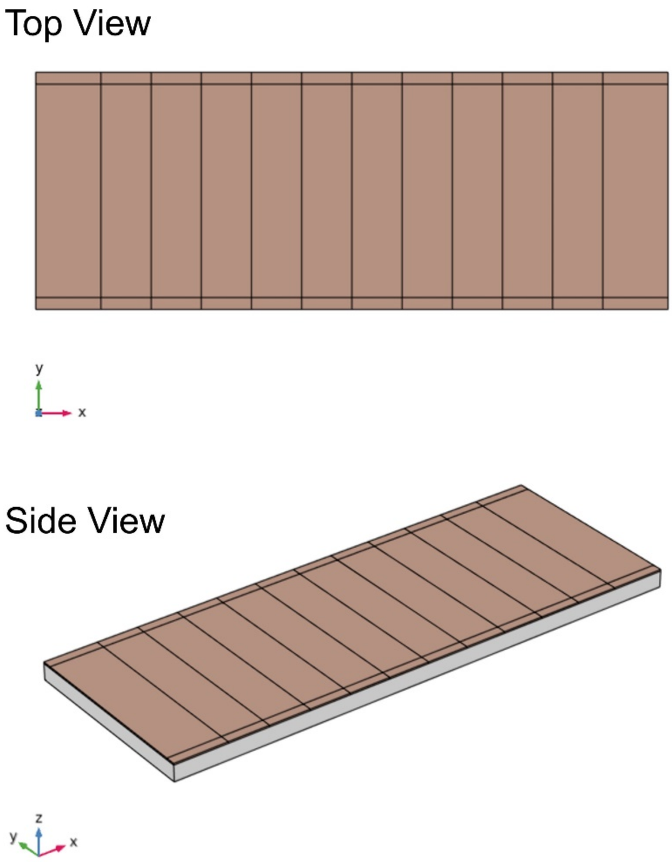

Supplementary Fig. 48. Cartesian coordinates and boundary conditions of FEA simulation. During the FEA simulation, the left boundary of FEA model was fixed, and the rest boundaries were set to be movable along x and y directions yet to with zero displacement along z-direction (the thickness direction).

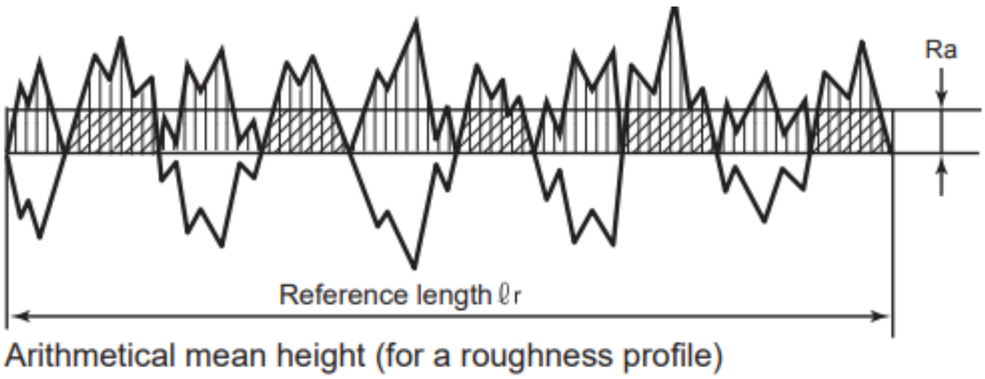

Supplementary Fig. 49. Characterization of arithmetical mean height (R_a).

a

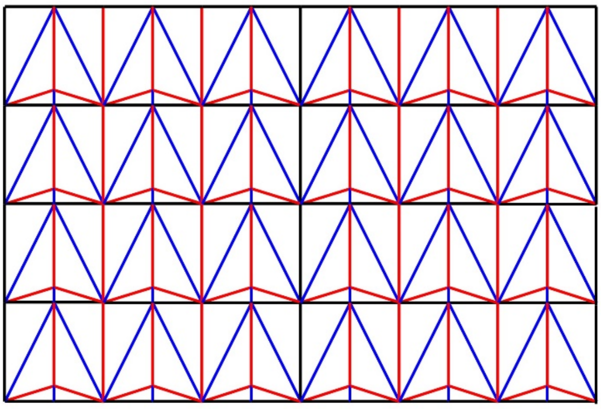

b

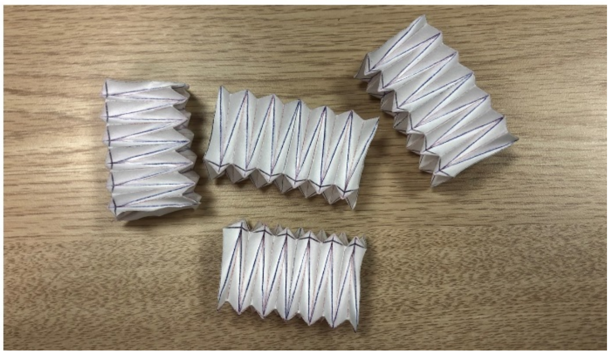

Supplementary Fig. 50. Crease pattern (a) and digital photo (b) of origami units.

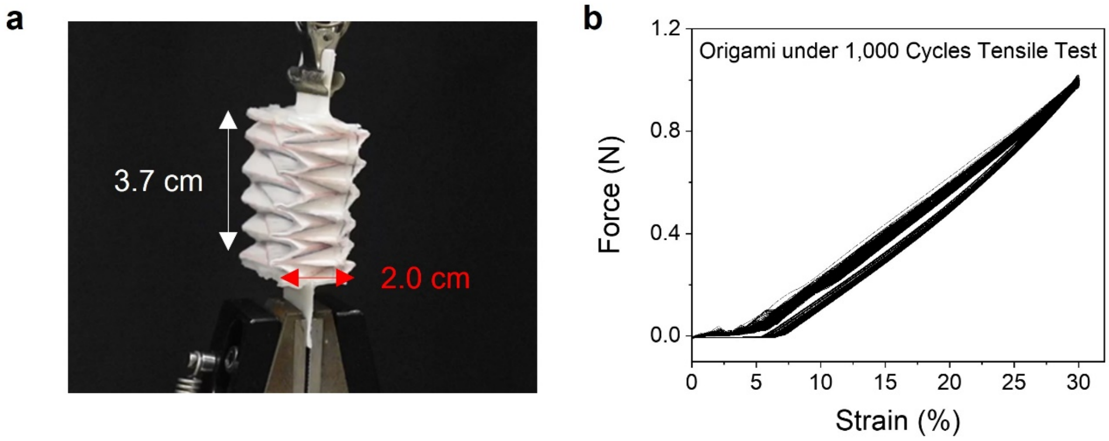

Supplementary Fig. 51. Mechanical stability of the Ecoflex-coated origami unit. (**A**) Dimension of an origami unit. (**B**) Force-strain curve of an Ecoflex-coated origami unit under 1,000 cycles.

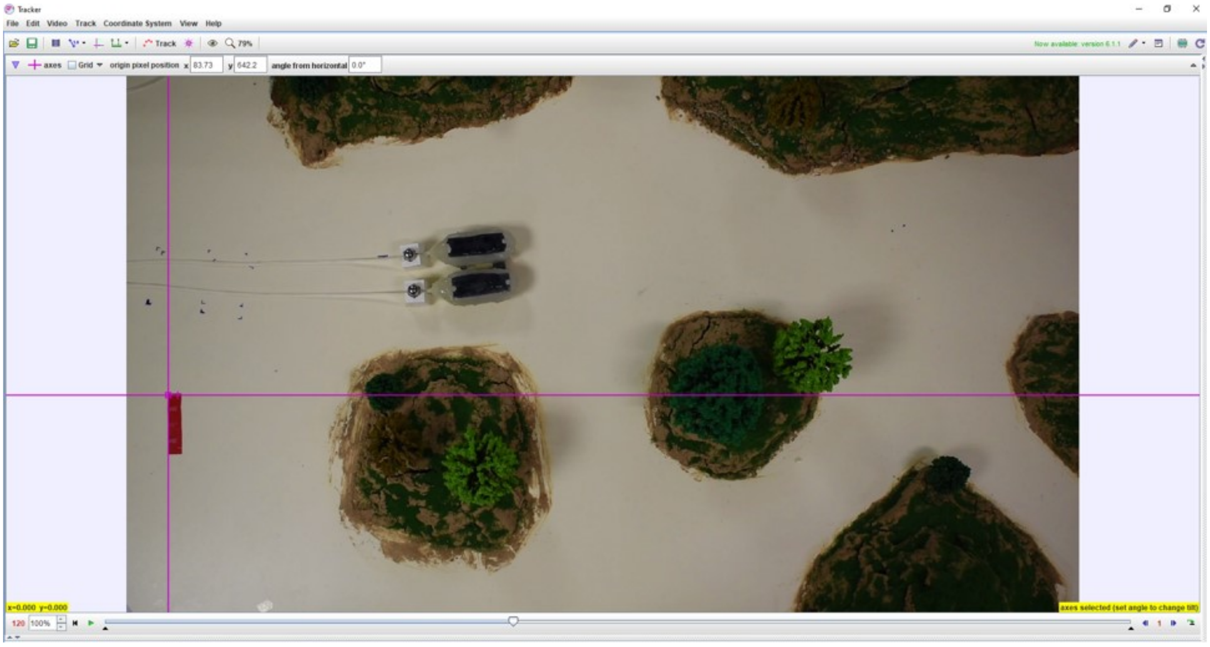

Supplementary Fig. 52. Working window of "Tracker" software.

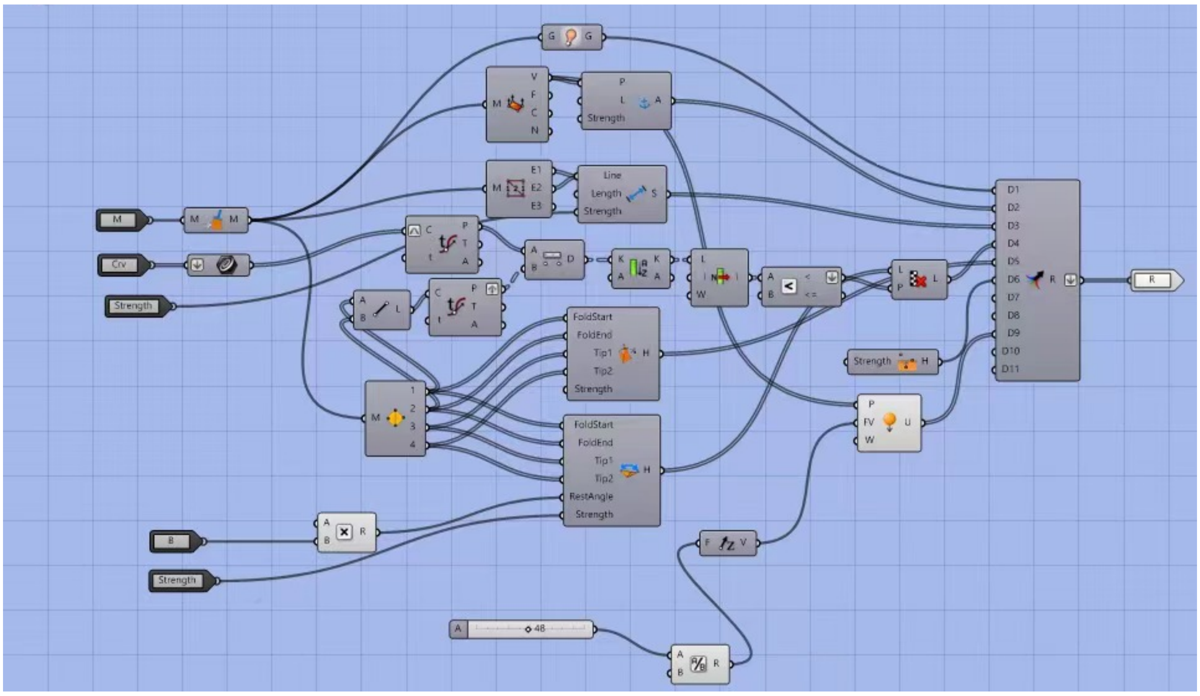

Supplementary Fig. 53. Kangaroo setup to simulate the origami structure's motions and deformations.

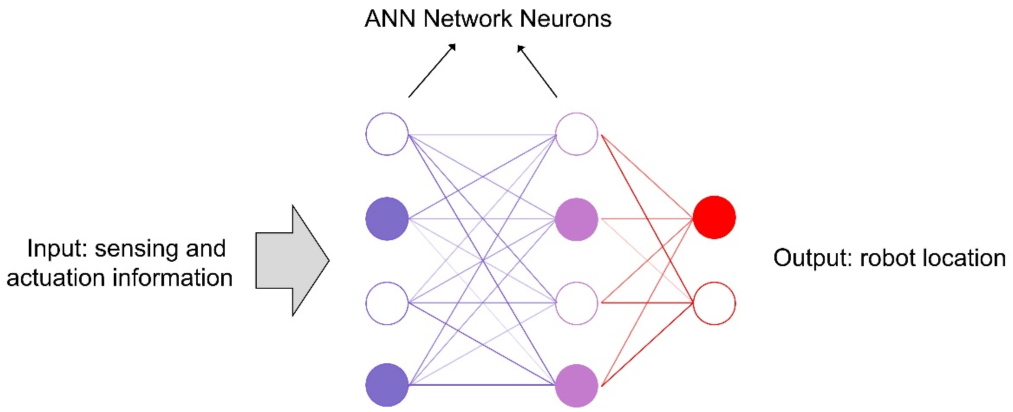

Supplementary Fig. 54. Working mechanism of ANN model.

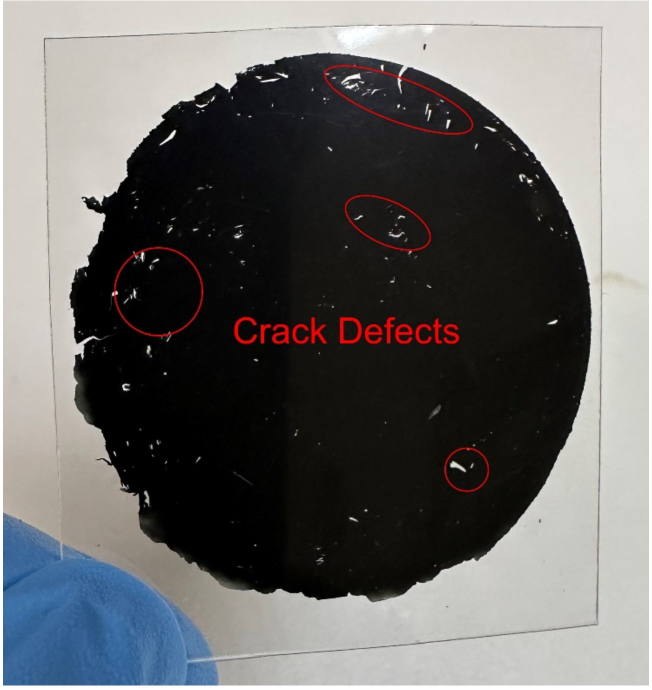

Supplementary Fig. 55. Digital photo of SWNT layer with 0.4 mg mass loading. Lots of crack defects were observed in the SWNT layer, which is not feasible for further sensor fabrication.

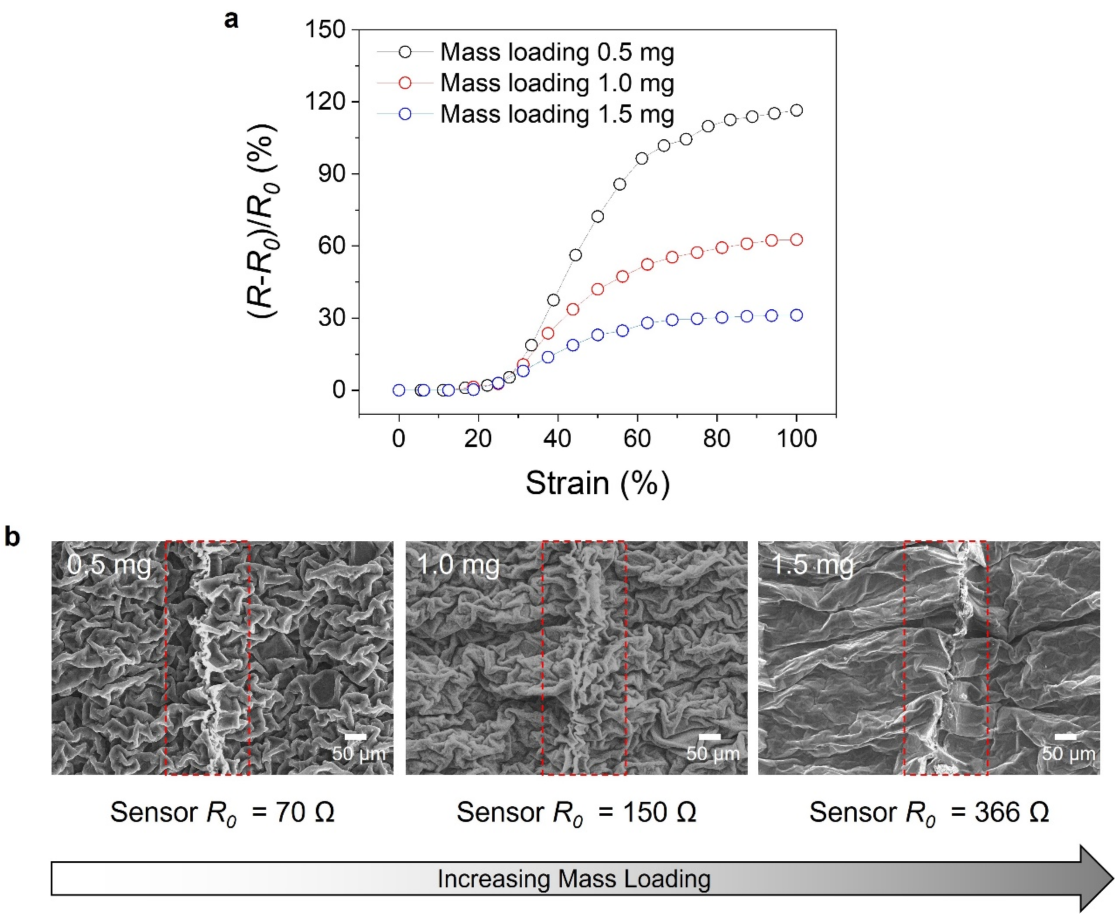

Supplementary Fig. 56. Mass loading optimization of a PCAM sensor. (**a**) Sensing profiles of the PCAM sensors with different mass loading from 0.5 to 1.5 mg. (**b**) SEM images of PCAM sensors with different mass loading from 0.5 to 1.5 mg.

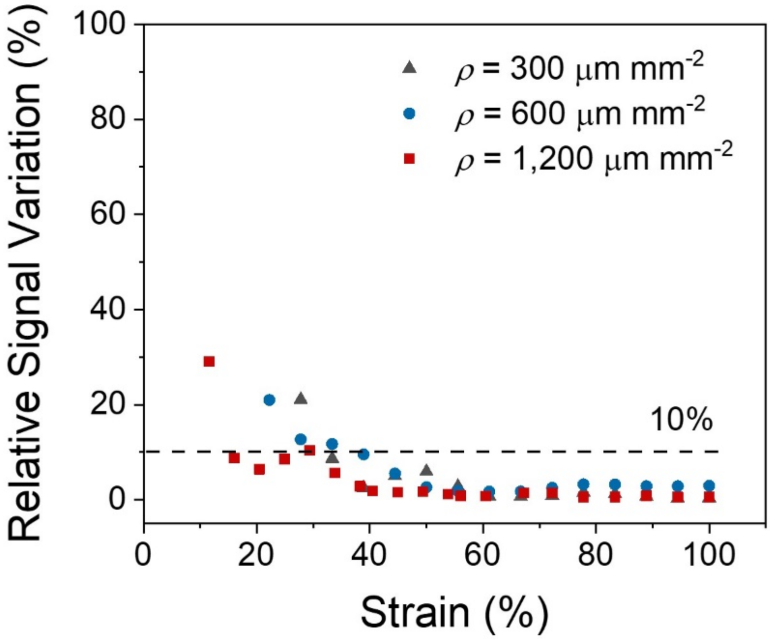

Supplementary Fig. 57. Relative signal variation of PCAM sensors based on three sensor replicas.

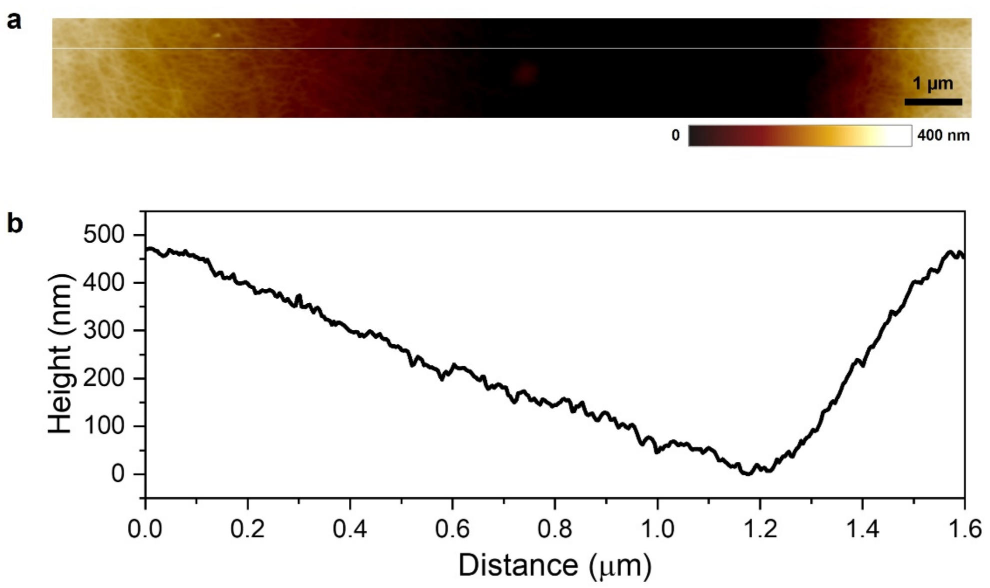

Supplementary Fig. 58. The etching depth of SWNT layer under the laser beam power of 0.06 mW. (**a**) AFM image of the SWNT layer. (**b**) Depth profile of the SWNT layer along the white line in (**a**).

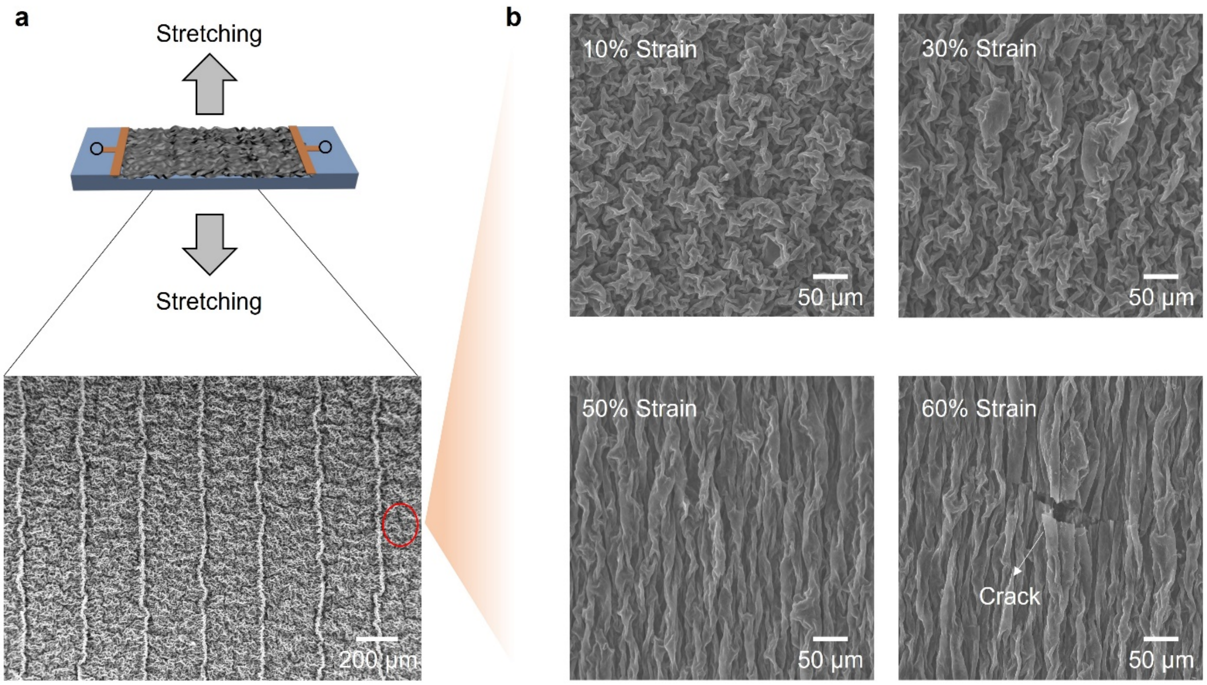

Supplementary Fig. 59. Noise strain tolerance of PCAM sensor. (**a**) During the test, a uniaxial strain that is perpendicular to the long axis of the sensor was applied on the PCAM sensor. (**b**) SEM images showed the structural evolutions of the PCMA sensor under a vertical strain loading from 10% to 60%. No crack emerged before 50% strain.

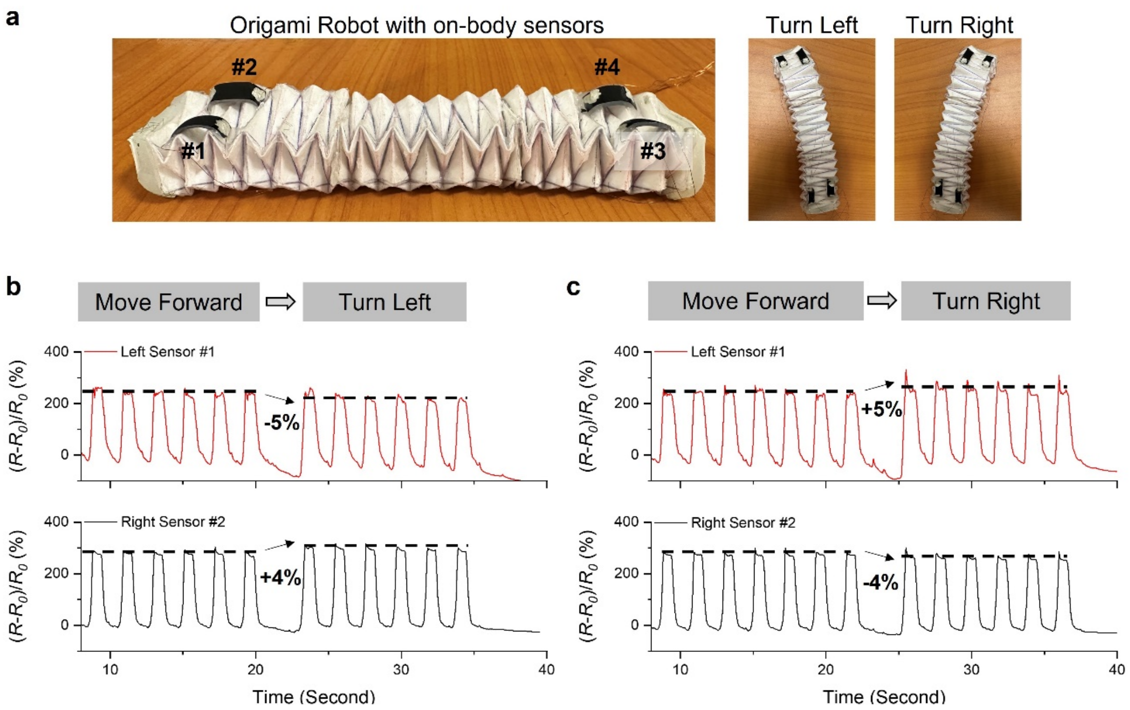

Supplementary Fig. 60. Sensing profiles of sensor-integrated origami robot when it was turning around. (**a**) Digital photo of an origami robot with 4 PCAM sensors at heads and tails. (**b**) Sensing profiles of a sensor-integrated origami robot during the movements: it first moved forward then turned left. (**c**) Sensing profiles of a sensor-integrated origami robot during the movements: it first moved forward then turned right.

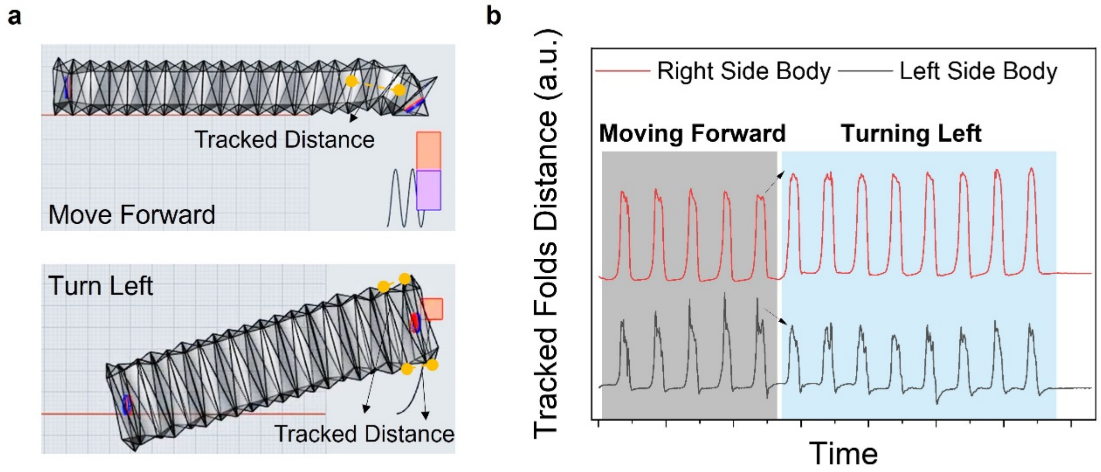

Supplementary Fig. 61. Simulation of origami deformation during turning. (**a**) Simulated movements of origami robot during moving forward and turning left. (**b**) Extracted folds distance changes between the designated two folds at both left and right sensor locations during moving forward and turning left.

a

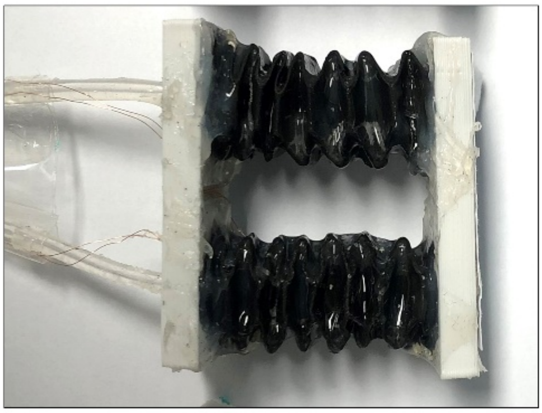

b

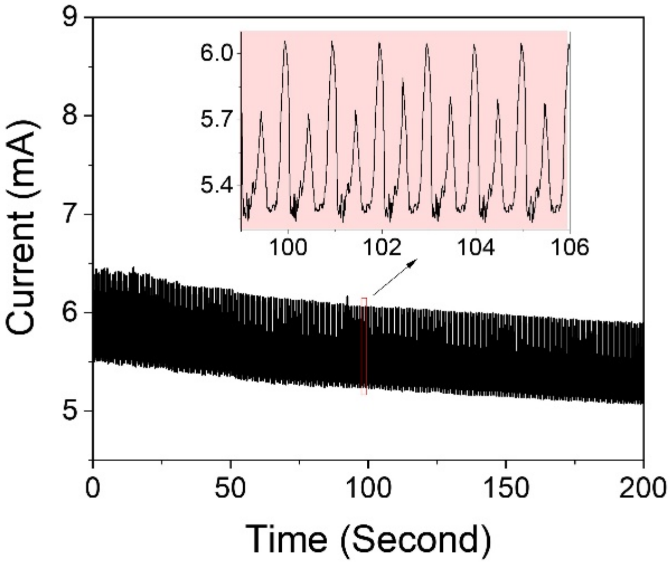

Supplementary Fig. 62. Digital photo and sensing profile of the origami robot from our previous work[3] under a 35-cm trajectory. After calculation, the signal deviation reached 67% after completing one iteration of a 35-cm trajectory (moving forward mode). (**a**) Digital photo of the origami robot. (**b**) Sensing profile of the origami robot under a 35-cm trajectory.

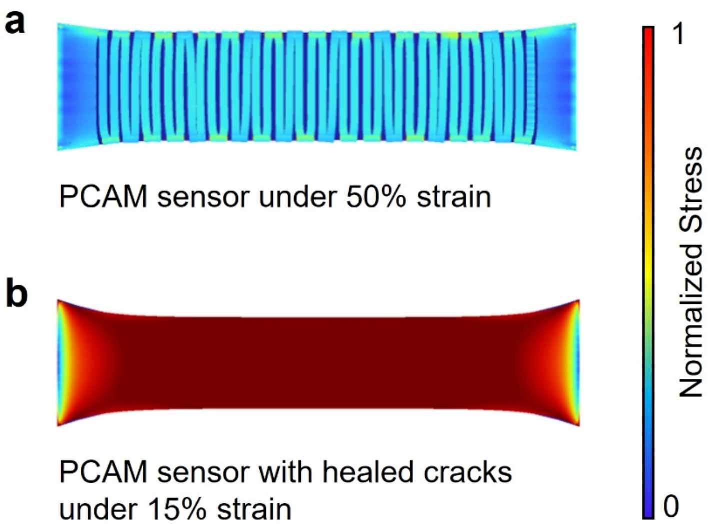

Supplementary Fig. 63. Simulated stress distribution maps of PCAM sensors. (**a**) PCAM sensor under 50% uniaxial strains (φ=40%, ρ=1,200 µm mm^{-2}). (**b**) PCAM sensor with fully healed cracks under 50% uniaxial strains (φ=40%, ρ=1,200 µm mm^{-2}).

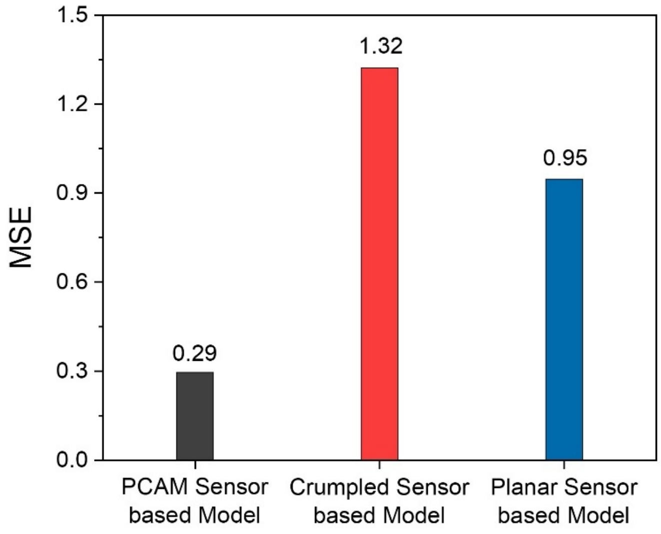

Supplementary Fig. 64. MSE of trajectory prediction model based on the training data from PCAM sensor, crumpled sensor, and planar sensor.

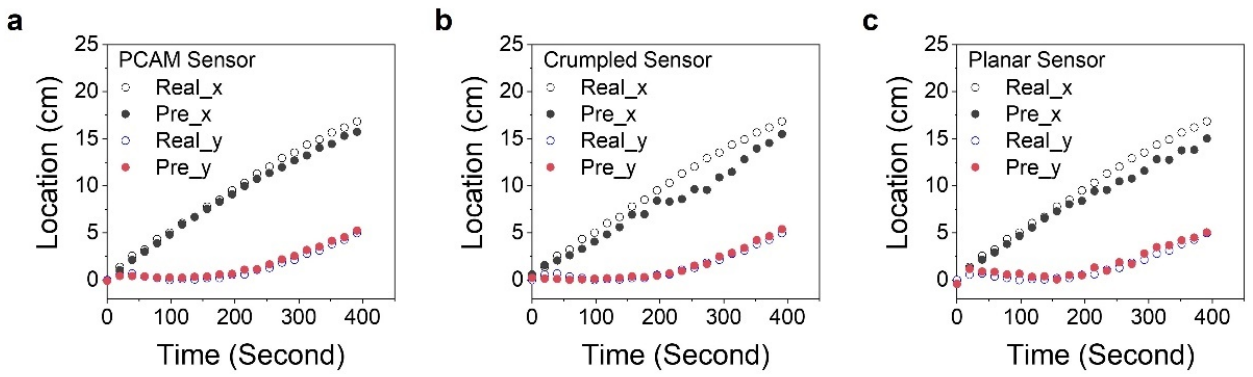

Supplementary Fig. 65. Prediction results of trajectory prediction model trained by data based on PCAM sensor, crumpled sensor, and planar sensor, respectively. (**a**) Prediction results of trajectory prediction model trained by PCAM sensor data. (**b**) Prediction results of trajectory prediction model trained by crumpled sensor data. (**c**) Prediction results of trajectory prediction model trained by planar sensor data.

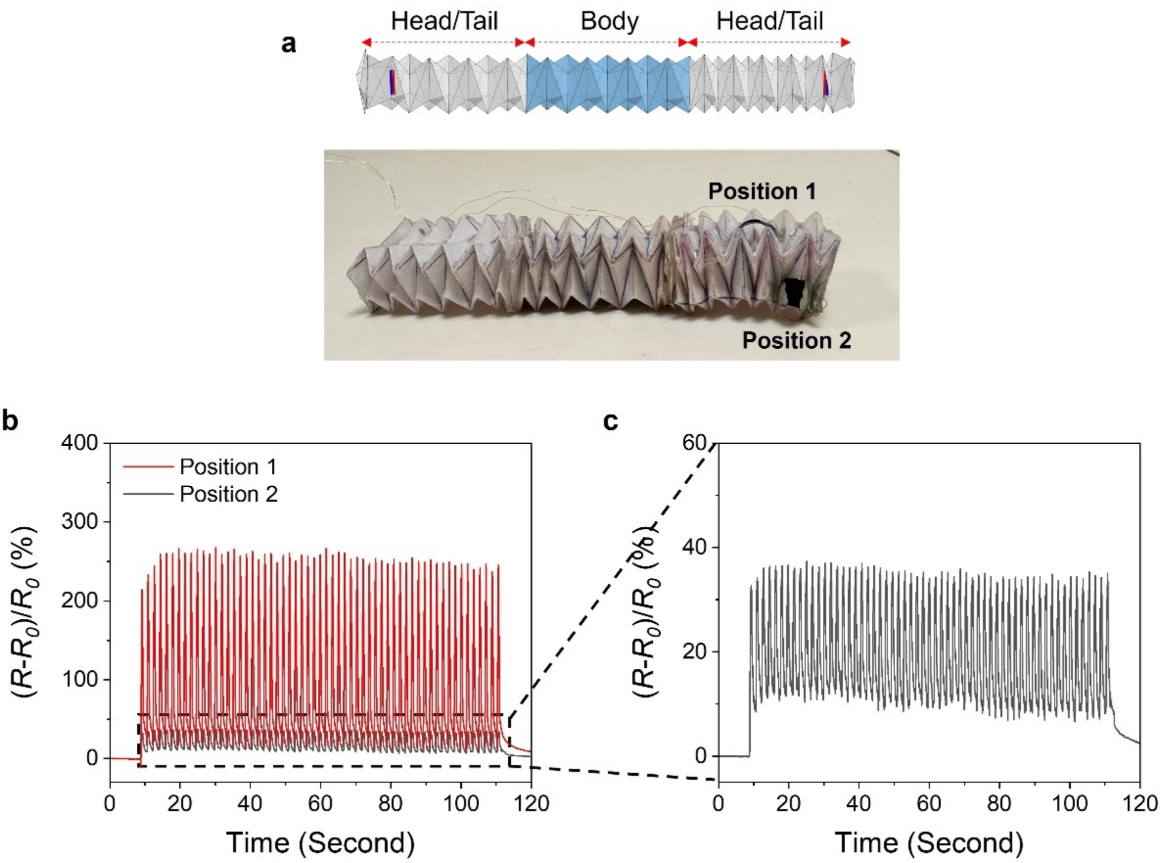

Supplementary Fig. 66. Sensor position optimization on origami robot. When monitoring the robot crawling motion, PCAM sensor at position 1 showed much higher signal response than position 2 thus is selected as the target position for constructing the body sensor network. (**a**) Digital photo of sensor positions on an origami robot. (**b**) Sensing profiles of sensors at positions 1and 2 when the robot was crawling. (**c**) Amplified sensing profile of sensor at position 2 when the robot was crawling.

Supplementary Table 1. Data summary of Fig. 6d. This table summarized the sensing signal amplitudes of the origami robot when it was climbing over six different hills. There is a simple and direct mapping relation between the hill heights and sensor signal amplitudes: a larger sensing signal amplitude refers to a higher hills. Based on the results, different hill heights were easily distinguished: #1 is the 4.5 mm hill, #2 is the 3.0 mm hill, #3 is the 1.5 mm hill, #4 is the 1.5 mm hill, #5 is the 3.0 mm hill, and #6 is the 4.5 mm hill.

Index of Signal Plateau	Sensor Signal Amplitude	Real Hill Height (cm)
1	27.1	4.5 mm
2	21.7	3.0 mm
3	16.8	1.5 mm
4	16.7	1.5 mm
5	21.7	3.0 mm
6	27.6	4.5 mm

Supplementary Table 2. The state-of-the-art soft crawling robot perception by mechanical sensors.

Reference	Robot sensor type	Multimodal locomotion	Basic soft robot perception			Advanced soft robot perception		Across-scale soft robot adaptability
			On-body sensing	Obstacle detection	Surface roughness classification	Trajectory prediction	Surrounding awareness	
H. Cui, et al. *Science*, 2022, 376, 1287. [1]	Piezoceramic sensor	√	√	√	×	×	×	×
Y. Zhao et al., *Sci. Robot.*, 2021, 6, eabd5483. [2]	Piezoresistive sensor	×	√	√	×	×	×	×
H. Yang, et al. *Sci. Robot.*, 2020, 4, eaax7020. [3]	Piezoresistive sensor	√	√	×	×	×	×	×
H. Bai, et al. *Sci. Adv.*, 2022, 8, eabq2104. [4]	Waveguide sensor	√	√	×	×	×	×	×
X. Wang, et al. *Adv. Mater.*, 2020, 32, 2000351. [5]	Piezoresistive sensor	√	√	×	×	×	×	×
K. Li, et al. *Adv. Funct. Mater.*, 2022, 32, 2110534. [6]	Piezoresistive sensor	√	√	×	×	×	×	×
H. Dong, et al. *Soft Robot.*, 2022, 6, 1198. [7]	Piezoresistive sensor	√	√	√	×	×	×	×
Y.-H. Lin, et al. *Adv. Intell. Syst.*, 2021, 2000244. [8]	Piezoresistive sensor	√	√	×	×	×	×	×
R. Goldoni, et al. *ACS Appl. Mater. Interfaces*, 2020, 12, 43388. [9]	Piezoresistive sensor	√	√	√	×	×	×	×
**This work**	Piezoresistive sensor	√	√	√	√	√	√	√

Supplementary Table 3. Details of stickiness factors during FEA modelling tasks.

	**Stickiness parameters between two crack boundaries**	**Stickiness parameters between SWNT and Ecoflex layers**
Penalty factor	1	1
Shear to normal ratio	0.8	0.9
Tensile strength	1.5×10^{3}	5×10^{10}
Shear strength	1.2×10^{3}	3×10^{10}
Tensile energy release rate	1.2 J mm^{-2}	1.2 J mm^{-2}
Shear energy release rate	1.2 J mm^{-2}	1.2 J mm^{-2}

Supplementary Table 4. Details of sensor structure parameters during FEA modelling tasks of different PCAM sensors.

Index	PCAM sensor structure parameter		Input parameter of SWNT layer in FEA model	
	ρ (μm mm^{-2})	φ (%)	Crack number	Young's modulus (MPa)
1	300	40	11	15.5
2	600	40	21	15.5
3	1,200	40	41	15.5
4	1,200	25	41	33.8
5	1,200	40	41	15.5
6	1,200	55	41	5.5

Supplementary Table 5. Comparison among the features of origami robot, pneumatic robot, and microrobot in this work.

	**Origami Robot**	**Pneumatic Robot**	**Microrobot**
**Modelling Tool**	Grasshopper software	Not available	Not available
**Locomotion Mode**	1) Moving forward<br>2) Moving backward<br>3) Turning left<br>4) Turning right<br>5) Climbing over hills	1) Moving forward<br>2) Turning left<br>3) Turning right	1) Moving forward<br>2) Moving backward<br>3) Turning left<br>4) Turning right
**Actuation Mode**	Untethered actuation	Tethered actuation	Untethered actuation

Supplementary Table 6. Comparison among the sensing characteristics of PCAM sensor and our previous work.

**Sensing characteristics**	**Sensor in our previous work[3]**	**PCAM Sensor (this work)**
**Gauge Factor (GF)**	Maximal 1.3	Maximal 204

**Linear Working Window**	Fixed by robot structure. (0-60%)	Tunable from 5%-50%, to 10%-70% and 20%-120%
**Sensing Performance Modelling**	Not available	FEA tool

Supplementary Table 7. Comparison among the GF of PCAM sensor and some recent self-healing strain sensors.

**Reference**	**Self-healing materials**	**Maximal GF**
F. Sun, et al. *Nat. Commun.,* 2023, 14, 130.[10]	Yes	1.0
Y. Wang, et al. *Adv. Funct. Mater.*, 2023, 33, 2301587.[11]	Yes	3.2
H. Fu, et al. *Mater. Horiz.,* 2022,9, 1412–1421.[12]	Yes	2.2
D. Hardman, et al. *NPG Asia Mater.,* 2022, 14, 11.[13]	Yes	1.5
T. Dai, et al. *J. Mater. Chem. C,* 2022,10, 15532–15540.[14]	Yes	1.3
S. Liu, et al. *ACS Appl. Polym. Mater.,* 2020, 2, 3, 1325–1334.[15]	Yes	0.14
G. Cai, et al. *Adv. Sci.,* 2017, 4, 1600190.[16]	Yes	1.5
**This work**	No	204

Supplementary Table 8. Comparison between this work and other recent works that coupled strain sensors and ML algorithm to identify different hand gestures or body pose.

**Reference**	**Task of ML model (prediction accuracy)**	**Number of model training sample**
Y. Luo, et al. *Nat. Electron.* 2021, 4, 193–201.[17]	Human pose classification (99.7%)	72,336
M. Wang, et al. *Nat. Electron.* 2020, 3, 563–570.[18]	Hand gesture classification (100%)	3,000
Z. Zhou, et al. *Nat. Electron.* 2020, 3, 571–578.[19]	Hand gesture classification (98.6%)	660
H. Yang, et al. *Nat. Commun.* 2022 13, 5311.[20]	Human pose classification (100%)	300
**This work**	Soft robot trajectory prediction (96%)	38

Supplementary Table 9. Performance of robotic trajectory prediction model with different k_fold values.

Model Parameter			Train	Validation	Test
**Epoch**	**Bath size**	**k_fold**	**MSE**	**MSE**	**MSE**
10	10	4	10.82	9.14	4.67
10	10	6	8.96	8.45	3.99
10	10	8	8.33	8.13	4.06
10	10	10	9.17	8.13	3.97

Supplementary Note 1. Optimization of SWNT mass loading.

Various conductive low-dimensional nanomaterials, such as silver nanowires[21,22], carbon nanotubes[23,24], and MXene nanosheets[25,26], have been adopted for the fabrication of piezoresistive strain sensors. Among those nanomaterials, the single-walled carbon nanotubes (SWNT) exhibit superior conductivity stability (Supplementary fig. 1), making them the ideal choice for piezoresistive sensor fabrication in this study. Detailed characterizations of SWNT including X-ray diffraction, atomic force microscopy (AFM), and scanning electron microscope (SEM) are provided in Supplementary figs. 1b-1d. The SWNT dispersion in deionized water underwent vacuum-assisted filtration to deposit a thin layer on the polyvinylidene fluoride (PVDF) membrane (diameter 3.8 cm). The as-filtered SWNT layer was then detached from the PVDF membrane in an ethanol bath and transferred onto a polystyrene (PS) shrink film for further sensor manufacture (see Supplementary fig. 3). When the SWNT mass loading was 0.5 mg, the deposited layer thickness was ca. 600 nm which could be fully detached from the PVDF membrane. But, if we further decrease the mass loading to 0.4 mg, SWNT layer was too thin to be completely detached. As shown in Supplementary fig. 55, there were many crack defects within the SWNT layer during detachment, which is not feasible for further sensor fabrication.

On the other hand, we also examined the cases of increasing SWNT mass loading to 1.0 and 1.5 mg. As expected, all SWNT layers were successfully detached from the PVDF membrane. After sensor fabrication, we tested their sensing curves and recorded data profiles in Supplementary fig. 56a, where the sensor with 0.5 mg mass loading showed the highest sensitivity. We conducted *in situ* SEM to explore the mechanism. As shown in Supplementary fig. 56b, with increasing mass loading from 0.5 to 1.5 mg, the resulting sensor showed larger isotropic crumples which caused less-connected crack boundaries and led to higher original resistance from 70 to 366 Ω. According to Equations 1 and 2 in the main manuscript, it is reasonable that higher original resistance (R_0) leads to lower sensitivity (i.e., GF).

Considering the above discussions, in this study, 0.5 mg SWNT mass loading was selected for sensor fabrication.

Supplementary Note 2. Sensor reproducibility with high crack density.

To check the sensor reproducibility, we tested three sensor replicas and added error bars in Fig. 1g for PCAM sensors at higher crack density (ρ=600 and 1,200 μm/mm^{-2}). Indeed, the sensor signal variations increased when ρ value increased from 300 to 1,200 μm/mm^{-2} with higher crack density. We also calculated the relative signal variation based on Equation S1,

$$Relative\ Signal\ Variation = \frac{S_{\delta_\varepsilon}}{\delta_\varepsilon} \quad \text{(S1)}$$

, where δ_ε is the relative resistance change of PCAM sensor at ε strain, and S_{δ_ε} is the standard deviation of δ_ε from three sensor replicas at ε strain. According to Supplementary fig. 57, under different crack density (ρ) values, PCAM sensors showed similar relative signal variations, which were <10% for most working windows. These results indicated a good performance reproducibility of the PCAM sensor.

Supplementary Note 3. PCAM sensor with GF > 200.

High sensor GF > 200 was achieved by controlling the laser etching depth of the SWNT layer. First, by setting the laser beam power as 0.06 mW during the laser processing, the SWNT layer along with the interdigital lines was etched at a limited depth but not completely cut through. As shown in AFM images in Supplementary fig. 58, the laser etching depth was ca. 460 nm, which was 77% of the SWNT layer thickness (ca. 600 nm, see Supplementary fig. 2) and the produced interdigital pattern was named as trace pattern. Under such a condition, after sensor fabrication, we found cracks still grew along the trace pattern and the GF of the resulting sensor was greatly improved from 35 to 204. We conducted *in situ* SEM to explore the mechanism. As shown in Supplementary fig. 8, with limited etching depth, the resulting PCAM sensor showed well-connected crack boundaries and led to much lower original resistance (R_0) from 70 to 14 Ω. According to Equations 1 and 2 in the main manuscript, it is reasonable that low original resistance leads to high GF.

Supplementary Note 4. Discussion to future reduce the sensor hysteresis.

By coating a thin Ecoflex layer on the sensor top surface, a sandwiched structure of sensor could decrease hysteresis and improve wear resistance. However, on the other hand, an additional Ecoflex layer on the top surface also increase the complexity in sensor performance modelling, and the encapsulation of wire electrodes inside the sensor also need a proper design. Therefore, the consideration of performance balance is required.

Supplementary Note 5. Noise strain tolerance of PCAM sensor.

The tolerance of the PCAM sensor to sustain noise strain is evaluated by the maximum applied strain to keep microstructural integrity. In this study, we conducted *in situ* SEM to explore the structural evolution of PCAM sensors. As shown in Supplementary fig. 59a, a uniaxial strain that is perpendicular to the long axis of the sensor (i.e., sensing direction) was applied, and Supplementary fig. 59b recorded the microstructures under different strains. The results indicated that, within 50% strain, there was no damage on the SWNT layer due to the existence of micro-crumples features. However, when there were larger strains (>60%), unexpected surface cracks emerged, and the sensor structure was damaged. Therefore, 50% is regarded as the boundary of failure strain.

Supplementary Note 6. Actuation and control of the origami robot.

In this study, a self-designed mobile platform was developed to control the origami robot automatically. As shown in Supplementary fig. 42, the mobile platform consists of a linear motor and a four-wheel drive car, where a cubic Nd-Fe-B magnet (dimension, 20 mm) is placed on the top of the linear motor. Using such a system, we can realize varying magnet positions in a 3D space by two parts: (1) horizontal direction movement of magnet is achieved by the driving car on the ground, and (2) vertical direction movement of magnet is achieved by the up-and-down control of the linear motor. With both horizontal and vertical movements of the Nd-Fe-B magnet, a varying magnetic field in 3D space was realized by the mobile platform. It

is worth noting that the movement speed of both car and linear motor is well controlled by the pulse width modulation to achieve the desired movement pathway of the magnet. The basic movement of the linear motor includes moving up and down, while the car movement includes moving forward, moving backward, spinning left, spinning right, and stopping.

The robot control logic through the mobile platform is shown in Supplementary fig. 42 and explained by the following:

(1) Crawling forward. The crawling forward motion of the robot starts from the initial state, where the mobile platform is right below the robot head and the magnet is at the lowest position. Then the linear motor pushes the magnet up and down, and the car keeps moving forward. The time delay t_1 determines the motion cycle of the robot.
(2) Crawling backward. If the robot needs to crawl back, the mobile car first moves backward so that the magnet and the tail of the robot align vertically (time delay t_2 is decided by the robotic body length). The loop-2 controls the car moving backward, while the linear motor keeps moving up and down. When the crawling back motion stops, the platform returns to the initial state to start the next robot actuation task.
(3) Turning left/right. When adding a car spinning left or right movement before crawling forward, the robot can make a left/right turn.
(4) Stop. When the robot needs to stop, the control loop (loop-1) breaks and returns to the initial state by moving down the magnet and stopping the car.

Supplementary Note 7. Sensor signal analyses of origami robot.

By comparing the on-body sensing profiles, different actuation states of the origami robot could be distinguished. As shown in Fig. 4a, four PCAM sensors were attached to the robot head and tail at symmetrical positions. According to Supplementary Video 6, the origami robot is able to move left/right by controlling the magnetic field movement trajectory, and Supplementary fig. 60 recorded the sensing profiles. When turning left, as indicated in Supplementary fig. 60b, the sensor attached at the left side of the robot head showed increased sensor signals while the right-side sensor showed reduced sensor signals. Such a sensor signal trend is due to an opposite origami deformation between the left and right sides when making a left turn. This principle is validated by the Grasshopper software simulation (see details in Methods). As shown in Supplementary Video 6, origami body deformations during moving forward and turning left were simulated, and the distance changes between the designated two folds at both left and right sensor locations were extracted. Based on the extracted results in Supplementary fig. 61, when making a left turn, compared to the forward motion, the folds distance change at the left side becomes larger (body expansion) while the right side showed a reduced trend (body compression). Because larger body deformation induced larger sensor signals, the simulation results match the real sensing data well and explain the mechanism: when turning left, the right side of the origami body expands and the left side is compressed, leading to increasing sensor signal at the right side and reduced signals at the left side. Similarly, the right turn motion of the robot could be identified when there were reduced sensor signals from the right-side sensor but increasing signals from the left-side sensor (Supplementary fig. 60c).

On the other hand, the origami robot could distinguish different surfaces based on their roughness, such as the desktop (the arithmetical mean height (R_a) is 2.4 µm, see definition in

Methods), printing paper (R_a = 3.5 μm), watery surface (R_a = 0.6 μm), or meeting an obstacle. Basically, when the robot moves from a smooth surface to a rough surface (e.g., from the desktop to a printing paper or meeting an obstacle), a reducing sensor signal was observed due to less body deformation (Supplementary figs. 22 and 23). On the other hand, when the robot moves from a rough surface to a smooth surface (e.g., from the desktop to a watery surface), an increasing sensor signal was observed due to larger body deformation (Supplementary fig. 24).

Supplementary Note 8. Actuation and control of the pneumatic robot.

In this work, we use customized pneumatic units to control the pneumatic robot. As shown in Supplementary fig. 30, this setup consists of two pneumatic units to control the left and right robot bodies, respectively. Each pneumatic unit has two sets of motors and air pumps, where one set (includes one motor and one air pump) is for exhaust and the other one set for inhalation. The microcontroller controls the exhaust and inhalation of the pneumatic units by deciding which air pump to work. With sufficient air pressure, the pneumatic unit is able to flex and extend the soft robot body. The time interval t_1 and t_2 between the switching of pumps determines the motion speed.

The robot control logic through the pneumatic units is shown in Supplementary fig. 30 and explained by the following:

(1) Crawling forward. Each robot body (left and right) is driven by one pneumatic unit. The crawling straight motion is completed by the synchronous inhalation and exhaust of pneumatic units A and B.
(2) Turning left/right. When one pneumatic unit is always inhaling, and the other is alternately inhaling and exhausting, the left and right bodies of the robot move differentially to turn. For example, turning left motion is realized when pneumatic unit A is always inhaling, while unit B is alternately inhaling and exhausting. Similarly, turning right motion is realized when pneumatic unit B is always inhaling, while unit A is alternately inhaling and exhausting.
(3) Stop. Turning off both pneumatic units A and B to stop the robot.

Supplementary Note 9. Reasons for selecting origami robot as robot autonomy demonstration.

In this work, we fabricated three kinds of soft robots, including origami robot, pneumatic robot, and microrobot, and the origami robot is selected in the demonstration of robot autonomy (i.e., trajectory prediction and topography altitude awareness) mainly due to two reasons including (1) mature robot modelling tool and (2) advantageous actuation behaviors.

Firstly, the Grasshopper software, a user-friendly modelling tool enables simulation of the body deformations of origami robot during movements (see details in Methods section). This capability allows for accurate interpretation of the integrated robot sensor signals, which is helpful for users to prepare the initial training datasets for developing the model of robot autonomy. For example, when constructing the terrain prediction model, based on the simulation results in Fig. 6c, we understand the signals differences in Fig. 6d indeed reflected

the origami body deformations when climbing over six different hills instead of signal fluctuations, which could serve as efficient training data to construct the prediction model. However, similar robot modelling tool for pneumatic robots or microrobots are not available.

Secondly, origami robot showed advantages regarding multi-modal locomotion and untethered actuation, which are key aspects for robot autonomy with higher motion freedom. As summarized in Supplementary Table 5, compared to pneumatic robots and microrobots, the origami robot has large number of locomotion modes as well as untethered actuation features, simultaneously.

Considering the above factors, the origami robot was selected in the robot autonomy application/demonstration.

Supplementary Note 10. Test of system error of the origami robot actuation.

The actuation system error of the origami robot is quantified by the distance deviation of the destination of robot trajectories among multiple repeated iterations. In this work, as shown in Supplementary fig. 43, the origami robot is controlled to achieve 112 cm navigation. Such navigation is repeated 5 times and corresponding robot trajectories are extracted from the recorded videos by Tracker (see Methods). The results in Supplementary fig. 43 indicated that there is a 0.8 cm deviation of the destination among 5 iterations of robot trajectories. Considering the total displacement is 112 cm, the relative system error is confirmed as 0.9%.

Supplementary Note 11. Comparisons of the pros and cons between PCAM sensors and our previous work.

One of our previous works also reported origami robot with sensing capability (named as Ref. robot)[3]. Although the sensing mechanism of the sensor in Ref. robot and PCAM sensor in this work were both based on crack propagation, their materials design concepts are totally different and the sensing characteristics of the PCAM sensor was improved a lot.

For the work in Ref. robot, the sensing materials were porous noble metals templated from ion-loaded cellulose paper, which realized effective multifunction integration of fire retardancy, resistive heating, sensing, and communication. However, from the perspective of sensing performance, it suffered from many disadvantages, including low sensitivity, fixed working window, poor sensing stability, and an inability to model its sensing performance. These issues were attributed to the uncontrolled crack growth and propagation of the templated materials under deformations, which is also a long-standing challenge for crack-based strain sensors[27,28]. In this work, by introducing the programmed cracks array within micro-crumples strategy, the crack array is pre-defined by a laser machine and the crack propagation routes were well regulated. As a result, compared with Ref. robot in Supplementary Table 6, PCAM sensor showed 150 times higher GF and tunable working window from 5%-50% to 20%-120%. Meanwhile, the deterministic crack propagation enabled accurate sensor performance modelling by using the FEA tool. These sensor characteristics are important for soft robot applications:

(1) High sensor sensitivity enables the robot to distinguish small body deformations during navigation tasks like climbing over a small hill in this work.

(2) Tunable sensor working window could satisfy the sensing requirements of diverse soft robots from origami to pneumatic, and across various scales.

(3) The FEA modelling tool allows virtual sensor performance verification without conducting trial-and-error experiments, facilitating the custom production of sensor-integrated soft robots.

In addition, we added supplementary experiments to compare signal stability between the sensor in Ref. robot and PCAM sensor. As shown in added Supplementary Fig. 62, the sensor in Ref. robot showed worse long-term stability under robot actuation. The signal deviation (see definition in equation 6) reached 67% within one iteration of a 35-cm trajectory, while PCAM sensors illustrate <6% signal deviation among 5 iterations of a 60-cm trajectory (see Supplementary Fig. 34). The robustness of sensor signals greatly enhances the perception capabilities of integrated soft robots and plays key roles in machine learning process for predicting the robot trajectory. More stable signals lead to a more accurate trajectory prediction model. This part is detailly discussed in the Supplementary Note 13.

Supplementary Note 12. Comparisons between PCAM sensors and recent self-healing sensors.

For the self-healing strain sensors, the self-healing materials may help to connect cracks together after cycling, yet we are afraid that the introduction of self-healing materials may simultaneously bring some disadvantages of crack-based sensor, including (1) sensor sensitivity, (2) working frequency, and (3) performance modelling.

Firstly, self-healing materials will reduce the sensor sensitivity. Self-healing materials are usually non-conductive polymers. Compared with the pure SWNT material of PCAM sensors, the introduction of self-healing materials into the active SWNT layer will reduce the conductivity of sensing layer and lead to low sensor sensitivity (i.e., gauge factor (GF)). We added Supplementary Table 7 to compare the GF between this work and recent self-healing strain sensors.

Secondly, self-healing materials will limit sensor's working frequency. Most self-healing materials require a long healing time up to minutes or even hours. For a very recent work of seal-healing actuation[29], its healing time is greatly reduced to 1 second. Such a healing time means it can sustain a maximal working frequency of 1 Hz. Given that state-of-the-art soft actuators operate within a range of mutative speeds (0.1-20 Hz) [30-33], it is essential to develop soft strain sensors to exhibit a stable sensing response across high operation frequencies, making them deployable for a broad range of soft robot applications. Our PCAM sensors have achieved this goal.

Thirdly but most importantly, self-healing materials will make the crack propagation behavior unpredictable, which is hardly to achieve sensor performance modelling. Self-healing materials usually experience a chemical bonding process between two interfaces, which is hard to simulate. On the other hand, once the cracks are healed, the stress-distribution map on the sensing layer will change from Supplementary Fig. 63a to 63b, and the route of crack growth

and propagation cannot be regulated by the pre-determined crack array anymore and become uncontrollable. As a result, the sensing response curves cannot be modelled. In this work, we developed FEA tools that could simulate the structure evolution of PCAM sensor, capable of predicting sensor's sensing curves without conducting trial-and-error experiments. This strategy bridges the gap between physical modelling techniques and empirical experimentation, which is the first report to the best of our knowledge.

Supplementary Note 13. Comparisons of the ML methodologies between this work and existing related reports.

In this work, ML is a general mathematical tool to analyze the sensing data and construct the prediction model. Although we did not develop a novel ML framework, our contribution here is the enhancement of learning efficiency from the sensor aspects, including (1) robust sensor signals and (2) high-resolution robotic sensor network.

ML allows for fast data analysis and prediction, yet it usually requires a large dataset for model training. Previously, researchers have developed smart gloves or textiles that coupled strain sensors and ML algorithm to identify different hand gestures or body poses[17 18-20]. However, as shown in Supplementary Table 8, these systems required a large amount of training data over than 300 samples. In this work, our developed PCAM sensor showed robust sensing signals with low signal deviation, which greatly improved learning efficiency of artificial neural network. The trajectory prediction model was constructed with 96% accuracy by using only 38 training samples.

We understand that it may not be entirely fair comparison between the references in Supplementary Table 8, due to their different ML tasks and application scenarios. To better investigate the effects from sensor data quality, we conducted additional ML experiments. As shown in Supplementary Figs. 33, 35, and 37, three types of sensors including PCAM sensor, crumpled sensor, and planar sensor were integrated into soft robot to perform a 25-cm turning trajectory for five times. While the signal deviations of PCAM sensor remained small (ca. 6%), those for the crumpled and planar sensors gradually increased to >50%. By using four iteration data as training set and adopting one iteration data as the testing set to construct the trajectory prediction model (see training and testing data files in GitHub). The model performance is evaluated by mean square error (MSE), where a lower MSE value indicates better model performance. As shown in Supplementary Figs. 64 and 65, the PCAM sensor-based prediction mode showed three times lower MSE and more accurate trajectory prediction on the test set, illustrating the advantage of high learning efficiency from robust sensor data.

On the other hand, high-resolution sensor network provides another approach to improve the learning efficiency, which were realized by minimizing the number of sensors on the key locations of robot body. For current ML applications on soft robots or actuators, the applied targets mainly refer to soft gloves[17-19] or soft grippers[34-37]. To capture their motions, there is no doubt to attach sensors on all gripper or glove fingers. However, for the crawling origami robots in this work, there are >40 possibilities of the sensor locations. Therefore, we need to optimize the sensor number and location on the robot body to collect the most representative key information of robot motion. For example, in Supplementary Fig. 66, sensor at position 1 showed higher sensitivity than position 2 when tracking the robot motions. The simulation

results in Supplementary Fig. 61 indicated that applying two sensors at origami head or tail is efficient for distinguish the turning direction. Therefore, to identify moving forward/backward and turning left/right motions, the minimized number and location of the sensors on robot was optimized as Supplementary Fig. 41. Besides, for the terrain prediction task, based on simulation results in Fig. 6a-6d, instead of four sensors at both robot head and tail, one sensor at head position is enough to distinguish the terrain height changes. These efforts simplified the relationship between sensor signals and robot motion to improve the learning efficiency. Therefore, simple ANN framework and <40 training samples were sufficient to generate the prediction model. To the best of our knowledge, as summarized in Supplementary Table 2, it is the first report to achieve robot autonomy on soft crawling robot (i.e., robotic trajectory prediction and topography altitude awareness).

Supplementary Note 14. The optimization of k_fold value in this work.

To explore the effects of k_fold values in ML process, we did extra experiments and tried different k_fold values when generating the ML model. The model performance is evaluated by MSE, where a lower MSE value indicates better model performance. As summarized in Supplementary Table 9, when using different k_fold values, the MSE of the prediction model on both validation and test sets were very similar, and the model with 10_fold validation showed the lowest MSE and thus was picked as the model parameter.

Supporting References

1 Cui, H. *et al.* Design and printing of proprioceptive three-dimensional architected robotic metamaterials. *Science* **376**, 1287-1293, (2022).

2 Zhao, Y. *et al.* Somatosensory actuator based on stretchable conductive photothermally responsive hydrogel. *Sci. Robot.* **6**, eabd5483, (2021).

3 Yang, H. *et al.* Multifunctional metallic backbones for origami robotics with strain sensing and wireless communication capabilities. *Sci. Robot.* **4**, eaax7020, (2019).

4 Bai, H., Kim, Y. S. & Shepherd, R. F. Autonomous self-healing optical sensors for damage intelligent soft-bodied systems. *Sci. Adv.* **8**, eabq2104, (2022).

5 Wang, X.-Q. *et al.* Somatosensory, light-driven, thin-film robots capable of integrated perception and motility. *Adv. Mater.* **32**, 2000351, (2020).

6 Li, K. *et al.* Thermal camouflaging MXene robotic skin with bio-inspired stimulus sensation and wireless communication. *Adv. Funct. Mater.* **32**, 2110534, (2022).

7 Dong, H., Yang, H., Ding, S., Li, T. & Yu, H. Bioinspired amphibious origami robot with body sensing for multimodal locomotion. *Soft Robot.* **9**, 1198-1209, (2022).

8 Lin, Y.-H. *et al.* Modeling and control of a soft robotic fish with integrated soft sensing. *Adv. Intell. Syst.* **5**, 2000244, (2023).

9 Goldoni, R. *et al.* Stretchable nanocomposite sensors, nanomembrane interconnectors, and wireless electronics toward feedback–loop control of a soft earthworm robot. *ACS Appl. Mater. Interfaces* **12**, 43388-43397, (2020).

10 Sun, F. *et al.* Vascular smooth muscle-inspired architecture enables soft yet tough self-healing materials for durable capacitive strain-sensor. *Nat. Commun.* **14**, 130, (2023).

11 Wang, Y. *et al.* High linearity, low hysteresis $Ti_3C_2T_x$ MXene/AgNW/liquid metal self-healing strain sensor modulated by dynamic disulfide and hydrogen bonds. *Adv. Funct. Mater.* **33**, 2301587, (2023).

12 Fu, H. *et al.* A self-healing, recyclable and conductive gelatin/nanofibrillated cellulose/Fe^{3+} hydrogel based on multi-dynamic interactions for a multifunctional strain sensor. *Mater. Horiz.* **9**, 1412-1421, (2022).

13 Hardman, D., George Thuruthel, T. & Iida, F. Self-healing ionic gelatin/glycerol hydrogels for strain sensing applications. *NPG Asia Mater.* **14**, 11, (2022).

14 Dai, T. *et al.* Self-adhesive, self-healing, conductive organogel strain sensors with extreme temperature tolerance. *J. Mater. Chem. C* **10**, 15532-15540, (2022).

15 Liu, S., Qiu, Y., Yu, W. & Zhang, H. Highly stretchable and self-healing strain sensor based on gellan gum hybrid hydrogel for human motion monitoring. *ACS Appl. Polym. Mater.* **2**, 1325-1334, (2020).

16 Cai, G. *et al.* Extremely stretchable strain sensors based on conductive self-healing dynamic cross-links hydrogels for human-motion detection. *Adv. Sci.* **4**, 1600190, (2017).

17 Luo, Y. *et al.* Learning human–environment interactions using conformal tactile textiles. *Nat. Electron.* **4**, 193-201, (2021).

18 Wang, M. *et al.* Gesture recognition using a bioinspired learning architecture that integrates visual data with somatosensory data from stretchable sensors. *Nat. Electron.* **3**, 563-570, (2020).

19 Zhou, Z. *et al.* Sign-to-speech translation using machine-learning-assisted stretchable sensor arrays. *Nat. Electron.* **3**, 571-578, (2020).

20 Yang, H. *et al.* Topographic design in wearable MXene sensors with in-sensor machine learning for full-body avatar reconstruction. *Nat. Commun.* **13**, 5311, (2022).

21 Yao, S. & Zhu, Y. Wearable multifunctional sensors using printed stretchable conductors made of silver nanowires. *Nanoscale* **6**, 2345-2352, (2014).

22 Zhu, G.-J. *et al.* Highly sensitive and stretchable polyurethane fiber strain sensors with embedded silver nanowires. *ACS Appl. Mater. Interfaces* **11**, 23649-23658, (2019).
23 Yamada, T. *et al.* A stretchable carbon nanotube strain sensor for human-motion detection. *Nat. Nanotechnol.* **6**, 296-301, (2011).
24 Kim, K.-H. *et al.* Enhancement of linearity range of stretchable ultrasensitive metal crack strain sensor via superaligned carbon nanotube-based strain engineering. *Mater. Horiz.* **7**, 2662-2672, (2020).
25 Bu, Y. *et al.* Ultrasensitive strain sensor based on superhydrophobic microcracked conductive $Ti_3C_2T_x$ MXene/paper for human-motion monitoring and e-skin. *Sci. Bull.* **66**, 1849-1857, (2021).
26 Pei, Y. *et al.* $Ti_3C_2T_x$ MXene for sensing applications: Recent progress, design principles, and future perspectives. *ACS Nano* **15**, 3996-4017, (2021).
27 Thuruthel, T. G., Shih, B., Laschi, C. & Tolley, M. T. Soft robot perception using embedded soft sensors and recurrent neural networks. *Sci. Robot.* **4**, eaav1488, (2019).
28 Glazar, J. T. & Shenoy, V. B. Data-driven design of soft sensors. *Nat. Mach. Intell.* **4**, 194-195, (2022).
29 Pena-Francesch, A., Jung, H., Demirel, M. C. & Sitti, M. Biosynthetic self-healing materials for soft machines. *Nat. Mater.* **19**, 1230-1235, (2020).
30 Acome, E. *et al.* Hydraulically amplified self-healing electrostatic actuators with muscle-like performance. *Science* **359**, 61-65, (2018).
31 Shi, Y.-X. *et al.* Soft electrochemical actuators with a two-dimensional conductive metal–organic framework nanowire array. *J. Am. Chem. Soc.* **143**, 4017-4023, (2021).
32 Tang, Y. *et al.* Leveraging elastic instabilities for amplified performance: Spine-inspired high-speed and high-force soft robots. *Sci. Adv.* **6**, eaaz6912, (2020).
33 Wu, Y. *et al.* Insect-scale fast moving and ultrarobust soft robot. *Sci. Robot.* **4**, eaax1594, (2019).
34 Lee, W. W. *et al.* A neuro-inspired artificial peripheral nervous system for scalable electronic skins. *Sci. Robot.* **4**, eaax2198, (2019).
35 Zhao, H., O'Brien, K., Li, S. & Shepherd, R. F. Optoelectronically innervated soft prosthetic hand via stretchable optical waveguides. *Sci. Robot.* **1**, eaai7529, (2016).
36 Yao, H. *et al.* Near–hysteresis-free soft tactile electronic skins for wearables and reliable machine learning. *Proc. Natl. Acad. Sci.* **117**, 25352-25359, (2020).
37 Jin, T. *et al.* Triboelectric nanogenerator sensors for soft robotics aiming at digital twin applications. *Nat. Commun.* **11**, 5381, (2020).
