# Supplementary material for: Computational design of ultra-robust strain sensors for soft robot perception and autonomy
Source: Nat Commun. 2024 Feb 22;15:1636. doi: 10.1038/s41467-024-45786-y (PMC10883982; doi:10.1038/s41467-024-45786-y)
Supplement: Supplementary file 3 — Description of Additional Supplementary Files [file 41467_2024_45786_MOESM3_ESM.pdf]

Description of Additional Supplementary Files

Supplementary Movie 1 : Electrical evolutions of a PCAM sensor under uniaxial strains.

Supplementary Movie 2 : PCAM sensor signal changes under a dynamic mechanical loading sequence.

Supplementary Movie 3 : Planar sensor signal changes under a dynamic mechanical loading sequence.

Supplementary Movie 4 : PCAM sensor performances under dynamic stretching frequencies across 1 to 10 Hz.

Supplementary Movie 5 : PCAM sensor stability under 23 Hz working frequency.

Supplementary Movie 6 : Origami robot with multimodal locomotion.

Supplementary Movie 7 : Robot movements and sensor profiles under 5 times repeat of trajectory 1.

Supplementary Movie 8 : Robot movements and sensor profiles under 5 times repeat of trajectory 2.

Supplementary Movie 9 : Origami robot passes through a small hill.

Supplementary Movie 10 : Comparison between the ANN-determined terrain altitudes and the ground truth.
